# Supplementary material for: Effects of an isoenergetic low Glycaemic Index (GI) diet on liver fat accumulation and gut microbiota composition in patients with non-alcoholic fatty liver disease (NAFLD): a study protocol of an efficacy mechanism evaluation
Source: BMJ Open. 2021 Oct 7;11(10):e045802. doi: 10.1136/bmjopen-2020-045802 (PMC8499287; doi:10.1136/bmjopen-2020-045802)
Supplement: Author's manuscript [file bmjopen-2020-045802.draft_revisions.pdf]

BMJ Open

BMJ Open is committed to open peer review. As part of this commitment we make the peer review history of every article we publish publicly available.

When an article is published we post the peer reviewers' comments and the authors' responses online. We also post the versions of the paper that were used during peer review. These are the versions that the peer review comments apply to.

The versions of the paper that follow are the versions that were submitted during the peer review process. They are not the versions of record or the final published versions. They should not be cited or distributed as the published version of this manuscript.

BMJ Open is an open access journal and the full, final, typeset and author-corrected version of record of the manuscript is available on our site with no access controls, subscription charges or pay-per-view fees (http://bmjopen.bmj.com).

If you have any questions on BMJ Open's open peer review process please email info.bmjopen@bmj.com

Effects of an iso-energetic low glycaemic index (GI) diet on liver fat accumulation and gut microbiota composition in patients with non- alcoholic fatty liver disease (NAFLD): An efficacy mechanism evaluation

Journal:	*BMJ Open*
Manuscript ID	bmjopen-2020-045802
Article Type:	Protocol
Date Submitted by the Author:	14-Oct-2020

Complete List of Authors:	Al-Awadi, Amina; University of Nottingham, Nottingham Digestive Diseases Centre<br>Grove, Jane; University of Nottingham, Nottingham Digestive Diseases Centre<br>Taylor, Moira ; University of Nottingham School of Life Sciences,<br>Valdes, A; University of Nottingham,<br>Vijay, Amrita; University of Nottingham, Nottingham Digestive Diseases Centre<br>Bawden, Stephen; University of Nottingham, Physics and Astronomy<br>Gowland, Penny; University of Nottingham, Department of Physics and Astronomy<br>Aithal, Guruprasad; Nottingham Digestive Diseases Biomedical Research Centre

Keywords:	Hepatology < INTERNAL MEDICINE, NUTRITION & DIETETICS, Hepatobiliary disease < GASTROENTEROLOGY

SCHOLARONE™
Manuscripts

I, the Submitting Author has the right to grant and does grant on behalf of all authors of the Work (as defined in the below author licence), an exclusive licence and/or a non-exclusive licence for contributions from authors who are: i) UK Crown employees; ii) where BMJ has agreed a CC-BY licence shall apply, and/or iii) in accordance with the terms applicable for US Federal Government officers or employees acting as part of their official duties; on a worldwide, perpetual, irrevocable, royalty-free basis to BMJ Publishing Group Ltd ("BMJ") its licensees and where the relevant Journal is co-owned by BMJ to the co-owners of the Journal, to publish the Work in this journal and any other BMJ products and to exploit all rights, as set out in our licence.

The Submitting Author accepts and understands that any supply made under these terms is made by BMJ to the Submitting Author unless you are acting as an employee on behalf of your employer or a postgraduate student of an affiliated institution which is paying any applicable article publishing charge ("APC") for Open Access articles. Where the Submitting Author wishes to make the Work available on an Open Access basis (and intends to pay the relevant APC), the terms of reuse of such Open Access shall be governed by a Creative Commons licence – details of these licences and which Creative Commons licence will apply to this Work are set out in our licence referred to above.

Other than as permitted in any relevant BMJ Author's Self Archiving Policies, I confirm this Work has not been accepted for publication elsewhere, is not being considered for publication elsewhere and does not duplicate material already published. I confirm all authors consent to publication of this Work and authorise the granting of this licence.

For peer review only

Title: Effects of an iso-energetic low glycaemic index (GI) diet on liver fat accumulation and gut microbiota composition in patients with non- alcoholic fatty liver disease (NAFLD): An efficacy mechanism evaluation

Authors: Amina Al-Awadi, Jane Grove, Moira Taylor, Ana Valdes, Amrita Vijay, Stephen Bawden, Penny Gowland, Guruprasad Aithal.

Corresponding Author: Guruprasad P. Aithal, Nottingham Digestive Diseases Centre, School of Medicine, University of Nottingham, Nottingham UK, NG7 2UH.
Email address: mszag3@exmail.nottingham.ac.uk
Tel: +44 (0)115 823 1031

AUTHORS AFFILIATIONS

Amina A. Al-Awadi, Nottingham Digestive Diseases Centre, University of Nottingham. NIHR Nottingham Biomedical Research Centre, Nottingham University Hospitals Trust and the University of Nottingham. Nottingham, United Kingdom.

Jane I. Grove, Nottingham Digestive Diseases Centre, University of Nottingham. NIHR Nottingham Biomedical Research Centre, Nottingham University Hospitals Trust and the University of Nottingham. Nottingham, United Kingdom.

Moira A. Taylor, Department of Nutrition and Dietetics, School of Life Sciences, University of Nottingham, Nottingham, United Kingdom.

Ana M. Valdes, NIHR Nottingham Biomedical Research Centre, Nottingham University Hospitals Trust and the University of Nottingham. Division of Rheumatology Orthopaedics and Dermatology, School of Life Sciences, University of Nottingham. Nottingham, United Kingdom

Amrita Vijay, Nottingham Digestive Diseases Centre, University of Nottingham. Division of Rheumatology Orthopaedics and Dermatology, School of Life Sciences, University of Nottingham. Nottingham, United Kingdom.

Stephen J. Bawden, Sir Peter Mansfield Imaging Centre, School of Medicine, University of Nottingham, Nottingham, United Kingdom.

Penny A. Gowland, Sir Peter Mansfield Imaging Centre, School of Medicine, University of Nottingham, Nottingham, United Kingdom.

Guruprasad P. Aithal Nottingham Digestive Diseases Centre, University of Nottingham. NIHR Nottingham Biomedical Research Centre, Nottingham University Hospitals Trust and the University of Nottingham. Nottingham, United Kingdom.

Key words: Nutrition and Dietetics, Hepatology, Hepatobiliary disease, Fatty liver, glycaemic index.
Word count: 6144

For peer review only

ABSTRACT:

Introduction A low glycaemic index (LGI) diet is a proposed lifestyle intervention in non-alcoholic fatty liver diseases (NAFLD) which is designed to reduce circulating blood glucose levels, hepatic glucose influx, insulin resistance, and *de novo* lipogenesis. A significant reduction in liver fat content through following a one-week LGI diet has been reported in healthy volunteers. Changes in dietary fat and carbohydrates have also been shown to alter gut microbiota composition and lead to hepatic steatosis through the gut-liver axis. There are no available trials examining the effects of a LGI diet on liver fat accumulation in patients with NAFLD; nor has the impact of consuming a LGI diet on gut microbiota composition been studied in this population. The aim of this trial is to investigate the effects of LGI diet consumption on liver fat content and its effects on gut microbiota composition in participants with NAFLD compared with a high glycaemic index (HGI) control diet.

Methods and Analysis A 2x2 crossover randomized mechanistic dietary trial will allocate 16 participants with NAFLD to a 2-week either HGI or LGI diet followed by a 4-week wash-out period and then the LGI or HGI diet, alternative to that followed in the first 2-weeks. Baseline and post-intervention (4 visits) outcome measures will be collected to assess liver fat content (using MRI/S and CAP-Fibroscan™), gut microbiota composition (using 16S RNA analysis) and blood biomarkers including glycaemic, insulinemic, liver, lipid and hematologic profiles, gut hormones levels, and short chain fatty acids (SCFAs).

Ethics and Dissemination Study protocol has been approved by the ethics committees of The University of Nottingham and East Midlands Nottingham-2

For peer review only - http://bmjopen.bmj.com/site/about/guidelines.xhtml

Research Ethics Committee (REC reference 19/EM/0291). Data from this trial will be used as part of a Philosophy Doctorate thesis. Publications will be in peer reviewed journals.

Trial registration number (NCT04415632)

For peer review only

ARTICLE SUMMARY:

Strengths

- This is a carefully controlled human intervention study that will provide evidence for the effects of a LGI diet on liver fat accumulation in NAFLD subjects and may serve as a complementary tool for reducing liver fat accumulation in these patients.
- The study will investigate associations between LGI dietary change and gut microbiota, between the change in microbiota and liver fat levels.
- Trial findings will facilitate the development of a novel strategy for NAFLD monitoring through understanding the mechanism behind the LGI dietary-related effects of specific gut microbiota and liver fat accumulation.
- Uses the controlled attenuation parameter (CAP™), a supplementary feature in transient elastography as a biomarker to evaluate efficacy of intervention.

Limitations:

- The short-term dietary intervention if efficacious need to be confirmed in long-term trials.
- Small sample size to investigate the mechanisms that will need to be confirmed in larger randomised controlled trial.

For peer review only

INTRODUCTION

Non-alcoholic fatty liver disease (NAFLD) is one of the growing global health problems and is the leading cause of chronic liver disease worldwide. The associated public health and economic burden are continuing to increase in parallel with the worsening epidemics of obesity and diabetes[1 2]. Recent reviews indicated that one billion people worldwide are affected by NAFLD; with an estimated prevalence of 25.4%[3]. The highest prevalence rates of NAFLD, ranging from 20% to 30% prevalence, have been reported in Western countries[4 5]. NAFLD is characterized by the fat accumulation in hepatocytes exceeding 5% of liver weight. It encompasses a spectrum of fatty liver diseases of increasing severity including steatosis, steatohepatitis, cirrhosis, and hepatocellular carcinoma; cases are expected to more than double between 2016-2030[6]. Common clinical risk factors that are highly associated with NAFLD include obesity, insulin resistance, type 2 diabetes, and metabolic syndrome[3 5]. Alarmingly high levels of NAFLD prevalence have been shown amongst obese adults with type 2 diabetes mellitus[7-9] as well as obese children[10 11]. NAFLD is recognized as the hepatic manifestation of metabolic syndrome[12-14].

It has been recently described that factors such as hormones secreted from the adipose tissue, dietary habits, gut microbiota composition and genetic factors are all implicated in the pathogenesis and development of NAFLD. Western dietary habits occurring concurrently with a sedentary lifestyle and genetic factors are associated with insulin resistance, adipocyte proliferation and gut microbiota alteration which may be implicated in NAFLD, a disease potentially with complex multifactorial causation[15].

For peer review only

To date, there are no drugs with regulatory approval for treating NAFLD, and lifestyle changes including modification of dietary patterns together with increased physical activity is the initial step to treat this disease[16]. Accordingly, several dietary interventions have been conducted on patients with NAFLD. In a prospective clinical study, a high protein hypo-energetic diet was positively associated with improvements in lipid profile, glucose homeostasis and liver enzymes in 48 stable NAFLD patients when compared with observations when they were consuming their baseline diet. However, their concurrent decrease in carbohydrate intake confounded these outcomes[17]. Evidence from a systematic review and meta-analysis of randomized control trials (RCT) on patients with NAFLD has shown that lifestyle-induced weight loss of ≥ 7% improved histological disease activity, as well as cardiometabolic risk profile, in less than 50% of the study population (n=373 patients)[18]. A recent systematic review has concluded that reductions in saturated and trans fatty acids, total carbohydrates, and animal-based protein intake and increases in the intake of polyunsaturated fatty acids (PUFAs), monounsaturated fatty acids (MUFAs), plant-based proteins, and antioxidants are the most beneficial dietary approaches to manage NAFLD[5]. Despite the abundance of interventional studies on dietary management in NAFLD, there is still a lack of evidence-based specific recommendations, other than that weight loss may benefit some; and few studies have measured liver-related outcomes[4]. Moreover, the dietary effects on liver-related outcomes that are mediated by gut microbiome have not been studied in these patients either[19].

More recently, there has been more focus on considering not only the amount of carbohydrate, but also the type of carbohydrates, in relation to health. The glycaemic

For peer review only

index (GI) of diet; has been proposed as an important predictor of NAFLD progression[5 20]. GI is a system to rank carbohydrates, and carbohydrate containing foods, on a scale from 0 – 100% according to the resulting glycaemic response following their consumption. The extent to which a portion of the food raises blood glucose after 2 hours from ingestion is compared with the value seen after consumption of glucose or white bread containing an equivalent quantity of carbohydrate[21 22]. Because the GI method is relying on a standard portion of carbohydrates to rank the tested carbohydrate food and this may not reflect the usual amounts consumed in a portion, another measure called the Glycaemic Load (GL) has been defined. GL is calculated based on the GI and the portion size of the food eaten (GL = [GI value x carbohydrates grams per serving] /100) and it has its own scale with values <10 being considered low and those > 20 considered high[23]. Whilst the GI of a food or diet reflects the glycaemic response to the food or diet under conditions in which the quantity of carbohydrate is controlled, the GL measures the overall glycaemic response to the food or diet taking into account the quantity of carbohydrate consumed.

Very little is currently known about the effects of GI and GL of the diet on NAFLD. Animal studies have shown that low GI (LGI) food consumption reduced the glycaemic excursions and food intake[24] and had a strong relationship with the parameters of metabolic syndrome and type 2 diabetes mellitus[25]. Whereas prolonged consumption of a high GI (HGI) diet was associated with a marked increase in body fat mass as well as liver fat, and impaired fat oxidation[26]. The diet of NAFLD patients has been characterized as containing HGI foods[5]. The consumption of HGI foods has been correlated with insulin resistance, metabolic dysfunction[5], increased inflammation and liver dysfunction[27]. Limiting sugar intake and HGI foods have preventive and

For peer review only

therapeutic effects on patients with NAFLD[28 29]. In addition, HGI dietary habits have been significantly associated with high-grade liver steatosis in patients with insulin resistance[30]. Further, previous pilot data showed that only one-week of consuming of HGI diet caused significant increase in hepatic stores of fat when compared with isocaloric LGI diet consumption[31]. Parkers and Kim (2019), have recently reviewed three studies investigating the effects of LGI and GL diets on hepatic fat mass in a total of 269 participants with NAFLD, including adults and children. They concluded that both low GI and GL diets showed significant reductions in hepatic fat mass when compared with baseline[23]. However, these effects were not attributed to the influence of LGI diet consumption alone and were confounded with other lifestyle interventions such as low-fat diet, low caloric foods, and moderate-intensity aerobic exercise[23]. Further work is needed to establish the importance of GI and GL in NAFLD, using well controlled dietary interventions.

In addition to the glycaemic response to carbohydrates being important in NAFLD there is evidence from animal and human studies that diet is the most important modifying factor in the development of human gut microbiome from infancy to old age[32-34]. Dietary alterations can rapidly change the human gut microbiota composition in as little as 24 hours[35-37]. This dietary-induced microbial alteration- dysbiosis- is due to different bacterial species utilization of different substrates[37 38]. For example, epidemiological studies have found that *Bacteroides* enterotype was positively associated with, in relative terms, long-term consumption of a high protein and animal fat diet, whereas the *Prevotella* enterotype was associated with a high carbohydrate diet[38-41].

For peer review only

Certain dietary factors which modify gut microbiota have been associated with a number of diseases including obesity[42] metabolic syndrome[32 43 44], type 2 diabetes[45], immunological dysfunctions[46], coronary vascular disease and inflammatory bowel diseases[33]. Such 'dysbiosis' in diet-induced NAFLD has been shown to stimulate hepatic fat disposition and promote NAFLD progression to non-alcoholic steatohepatitis (NASH)[47 48]. This was explained by the involvement of gut microbiota in gut permeability modulation, low-grade inflammation and immune balance, dietary choline metabolism modulation, bile acid metabolism regulation and endogenous ethanol production[47 48]. Through the gut-liver axis, gut microbiota modulated by diet can increase the energy extraction from the breakdown of non-absorbable polysaccharides with the production of monosaccharides and short chain fatty acids (SCFAs). Both monosaccharides and SCFAs are substrates for hepatic *de novo* lipogeneses and carbohydrates biosynthesis[49]. This demonstrates one of the mechanisms by which diet can modulate gut microbiota as a means of NAFLD prevention or alleviation[4 11].

There is thus some evidence that dietary induced insulin resistance and dysbiosis, lead to liver fat accumulation. We, therefore, aim to describe here the study protocol that is designed to assess the effects of a specific dietary manipulation, namely LGI diet consumption on liver fat content and its effects on gut microbiota composition in participants with NAFLD compared with a HGI diet as a control diet. The diets will be adjusted to each participant to meet their energy requirements and the macronutrient composition of the diets will be carefully controlled hence ensuring that differences in GL are due to differences in GI.

For peer review only

OBJECTIVES

Primary Objective

1. To investigate the effects of LGI versus HGI diet on liver fat accumulation in participants with NAFLD following a two-week intervention period whilst controlling for the energy consumption and amount of carbohydrate intake.

Secondary Objectives

1. To investigate the effects of LGI versus HGI diet on gut microbiota composition and its correlations with liver outcomes in participants with NAFLD following a two-week intervention period.
2. To investigate the changes in blood metabolic biomarkers (glycaemic, insulinemic, liver, lipid, hematologic profiles, gut hormones, and plasma SCFAs*)* between LGI and HGI diet groups following a two-week intervention in participants with NAFLD.
3. To evaluate CAP™ as a tool to test the efficacy of a dietary intervention by comparing its performance characteristics with that of Magnetic Resonance Spectroscopy (MRS) as a gold standard.
4. To validate the European Prospective Investigation of Cancer food frequency questionnaire (EPIC-FFQ) in Nottingham NAFLD patients against food diary.

For peer review only

METHODS AND ANALYSIS

Trial Design

This is a randomized 2 x 2 cross-over mechanistic trial including two arms, each of two weeks duration comparing a LGI diet and a control HGI diet. The study design in illustrated in Figure 1.

Public and patient involvement

Ahead of this current study application, the NIHR Nottingham Biomedical Research Centre (BRC) liver and gastrointestinal disorders patients and public involvement (PPI) department have been consulted at meetings including NAFLD patients in 2017 and 2018. They expressed strong support for the research objectives and the recent meeting the group considered the one-week intervention feasible and acceptable to participants.

As the current study is longer in duration (two-week intervention for each diet group), we collaborated with PPI department from the NIHR Nottingham BRC, where we met patients who are interested in study participation. The purpose of the PPI meeting was to help us seek and develop a sound, ethical, and participant friendly research design. After presenting the research objectives and flowchart design, the PPI team were asked to give their feedback and suggestions on the study protocol and the best way to conduct this research. Their feedback helped enhance the research quality from the participant's perspective in many ways. First, it improved the way we could approach and increase the participants' recruitment rate through information delivery methods and what participants need to know before participation. Second, it helped in understanding in what way we follow up the participants and enhance their compliance

For peer review only

and commitment to the diet throughout the study periods. Finally, they advised on increasing the information clarity of study advert and documentations.

Ethics and dissemination

Ethical approval was first obtained from the University of Nottingham, Medical School Research Ethics Committee. Then additional ethical approval was obtained from East Midlands Nottingham 2 Research Ethics Committee (The Old Chapel, Royal Standard Place, Nottingham). The trial is registered within ClinicalTrials.gov (NCT04415632). Data from this trial are intended to be presented at local and international conferences including those attended by clinicians and dietitians and will also be used as part of a Philosophy Doctorate thesis. Publications will be in peer reviewed journals.

Participants and Recruitment

We aim to recruit 16 participants with NAFLD to complete both phases of 2x2 cross-over trial. The initial screening criteria for inclusion is a CAP™ score of >288dB/m; this value correlates with Magnetic Resonance Imaging (MRI)- Proton Density Fat Fraction (PDFF) estimation of > 5% fat of liver weight which indicates that they have a fatty liver[50].

NAFLD patients will be recruited either from the existing database of "The Scarred Liver Project" in the UK who have given consent to be contacted to participate in future research (approved by East Midlands-Leicester Research ethics committee ref: B/EM/0123) or from liver clinics at the Nottingham University Hospitals NHS Trust who had a Fibroscan with CAP or participants from public (advert in local and social media). The eligibility criteria are summarized in Table 1. All participants will provide a written informed consent before they enter the trial.

For peer review only

Table 1: Eligibility Criteria
Inclusion criteria<br>• Adult males and females aged from 18 to 65 years (balanced number).<br>• Detected NAFLD by CAP-FibroScan™ >288dB/m or by MRI-PDFF) > 5% fat of liver weight.<br>• Body mass index ≥ 25 kg/m^2<br>• Abdominal obesity (Waist circumference > 102 cm for males and > 88 cm for females)<br>• Have current moderate to high GI diet intake of ≥ 60 (Assessed from a completed 7-day food diary).<br>• Able to give informed consent.<br>• Able to undergo MRI/S and CAP-FibroScan™.
Exclusion criteria<br>• Current smokers and excessive alcohol drinkers (> 14 units/week).<br>• Perimenopausal (irregular periods) women.<br>• Participants with other liver abnormalities.<br>• Participants with history of gastrointestinal surgeries, depression, eating disorders or difficulties.<br>• Participants using pharmacologic agents for obesity or NAFLD.<br>• Participants with type 1 diabetes.<br>• Participants with type 2 diabetes on second line medications (eg GLP-1 analogues).<br>• Participation in any other trial in the last 3 months.<br>• Participants on any special diets (e.g. vegetarians).<br>• Intolerance to foods included in the diet plan.

Screening process:

Participants will be invited to attend an initial screening visit (Figure 1). At this visit, participants will be consented and asked to complete a pre-screening questionnaire (including taking height, weight, and blood pressure measurements) to establish whether they meet the study criteria. Participants will be asked to fill out a 7-day food records and post it after one week of their initial screening visit. This is to avoid giving patients with NAFLD, who might be at risk of developing diabetes, a much higher HGI diet than they habitually consume. Using 7-day food records, we will characterise the baseline habitual GI diet intake of all potential volunteers at screening period. Participants also will be required to complete a food frequency questionnaire (FFQ)

For peer review only

for validation against the 7-day food records. We will use EPIC-FFQ that was validated for use in Norfolk cohort[51]. After completion, participant's eligibility will be reassessed based on analysis of diet information using Nutritics software (Nutritics Ltd, Dublin, Ireland, 2018). Those with a habitual LGI diet intake (GI < 60) will be excluded from the study. Only patients who have a moderate to HGI diet intake (GI ≥ 60) will be invited to attend the next screening preparation visit.

Then at next screening visit (visit 2), the participants will be asked to fill out International Physical Activity Questionnaire (IPAQ)[52]. A trained dietitian will instruct participants on how to measure study food provided using measuring jug and spoons and how to fill in study diet compliance sheets. They will be instructed on stool collection and pre-intervention visits requirements. Participants will be asked to avoid eating probiotics and/or prebiotics supplements or drinks for one week before the intervention period (standardization week). All instructions will be provided to each participant in a form of Home Guide Booklet.

Each eligible participant will be assigned randomly to undergo either HGI diet (HGI group) or LGI group first and then crossover to either HGI/LGI diets.

Randomisation and Blinding

Enrolled participants who meet the eligibility criteria will be randomized to one of two groups which will dictate the order in which they receive the LGI or HGI diet. They will then receive the second diet depending upon which diet they have first received. The random allocation sequence (AB or BA) will be generated by one of the trial co-ordinators; who will have no contact with the participants, using block randomization

For peer review only

by impartial biostatistics. The online randomisation package (http://randomization.com) will be used to allocate the sequences of the diet groups to the participants in equal numbers. Once the random number is generated, both trial investigators and participants are aware of the diet sequence (i.e. whether the participant is going to start with LGI diet; A or HGI diet; B). The diets then will be delivered to the participant according to the sequence. The trial outcome measures will be determined by blinded researcher using codes on samples that will be sent for 16S ribosomal ribonucleic acid (rRNA), blood marker and MRI/CAP-FibroScan™ analyses.

Study protocol:

Following the screening phase and standardization week, each participant will experience two, two-week intervention phases, with a four-week washout period between interventions. Before and after each intervention period they will attend the Imaging Centre (Sir Peter Mansfield Imaging Centre) for MRI scan measurements and then will attend the Biomedical Research Centre for Fibroscan, and blood and stool samples collection. During each intervention phase each participant will be required to consume all the food that has been provided, fill in dietary compliance sheets and complete visual analogue scales (VAS) relating to subjective appetite ratings.

For peer review only

Design of the dietary interventions.

Energy expenditure will be estimated for each participant using the individual's basal metabolic rate (BMR) calculated from Henry-modified Schofield formula[53] and multiplied by an activity factor based on the IPAQ score physical activity levels (PALs). A menu, consisting of meals for 3 days personalised for energy requirement, will be devised (these meals are repeated over 2 weeks) by adjusting a template according to their estimated energy requirement in order to minimise weight change during the intervention phase. The diet consists of normal food closely resembling that described by Bawden *et al*, (2017) study[31]. A diet template of 2000 kcal/day with energy proportions distributed as 55% from carbohydrates, 15% from protein and 30% from fat[54] are designed and food quantities will be modified to supply the energy requirements for each participant. Three-day cycle menu plans are designed for each diet (LGI/ HGI). Table 2 shows example template menus for both HGI (a) and LGI (b) diets.

For peer review only

Table 2 Illustration of HGI (a) and LGI (b) diet templates for use in the study.

HGI Diet Sample (a)

	Weight (g)	Energy (Kcal)	Carbohydrates (g)	Protein (g)	Fat (g)	Fibre (g)	GI	GL
Breakfast								
Sultana Bran Cereal	90	268	58	6.6	1.3	10.1	90	52.2
Skimmed Milk	180	63	8.3	6.3	0.54	0	48	4
Roasted Peanuts	15	90	2.3	3.7	7.4	1.3	14	0.32
Glucose	10	40	10	0	0	0	100	10
Water	170	0	0	0	0	0	0	0
Meal Total		461	78	16.6	9.2	11.4	85.3	66.5
Lunch								
Margarine	25	102	0.13	0.05	11.3	0	0	0
Fruit loaf	144	395	76	11.4	5.2	4.6	90	68.4
Roasted turkey slice	42	47	0.67	10	0.5	0.12	0	0
Cheddar cheese slice	25	104	0.03	6.4	8.7	0	0	0
Strawberry yogurt	125	88	16.3	5.6	0	0.38	85	13.855
Meal Total		735	93	33.4	25.7	5.1	88.44	82.255
Dinner								
Cottage Pie (ready meal)	400	441	44	18	21.6	4.8	66	29.04
Low fat Strawberry yogurt	125	88	16.3	5.6	0	0.38	85	13.855
Water	170	0	0	0	0	0	0	0
Glucose	10	40	10	0	0	0	100	10
Mars bar	58	237	37	2.4	8.9	0.87	65	24.05
Meal Total		806	107	26	30.5	6	72.69	76.954
Menu Total		2002 Kcal	278g (55.5%)	76g (15.2%)	65g (29.3%)	22g	82.1	225.706

LGI Diet Sample (b)

	Weight (g)	Energy (Kcal)	Carbohydrates (g)	Protein (g)	Fat (g)	Fibre (g)	GI	GL
Breakfast								
All Bran Cereal	90	239	42	11.2	3.1	22.1	43	18.06
Semi-Skimmed Milk	160	76	7.2	5.6	2.7	0	25	1.8
Orange juice	250	96	22	1.5	0.25	0	53	11.66
Meal Total		410	71	18.2	6	22.1	44.39	31.52
Lunch								
Pumpernickel bread	180	408	74	15.7	5.6	11.7	41	30.34
Black cherry yogurt	150	209	24.2	4.8	10.4	0.75	17	4.114
Soft cheese	51	129	1.5	2.7	12.4	0	34	0.51
Meal Total		745	99	23.2	28.4	12.5	35.32	34.96
Dinner								
Lasagne (ready meal)	290	413	42	21.5	17.7	4.9	36	15.12
Apple	174	92	20	1	0.87	2.1	38	7.6
Semi-skimmed milk	150	71	6.8	5.3	2.6	0	25	1.7
Fruit &nuts mix	60	267	24.6	8.4	15	3.9	15	3.69
Meal Total		843	93	36.2	36.1	10.9	30.22	28.11
Menu Total		1999 Kcal	264g (52.7%)	78g (15.5%)	71g (31.8%)	46g	36.64	94.594

The GI values of commercial food items are generated from that reported by Henry *et al*. (2005)[55], as well as Nutritics software databases (Nutritics Ltd, Dublin, Ireland, 2018). The dietary GI of the menu is calculated from GI proportional contribution to total carbohydrate using the equation: $GI = \sum(C_{food}/C_{total}) \times GI_{food}$; where C_{food} is the amount of carbohydrate contained in each digested food (g), C_{total} is the total carbohydrate in the meal and GI_{food} is the reported GI of the food[31]. The GL of the diet is calculated based on the dietary GI and the amount of carbohydrate provided (the same in both diets) using the following equation: $GL = (GI_{food} \times C_{food}) / 100$; where GI_{food} is the GI of each digested food and C_{food} is the amount of carbohydrate in the food[31]. Food composition analysis and evaluation were performed using Nutritics software (Nutritics Ltd, Dublin, Ireland, 2018).

Participants will be provided with the food at the start of each two-week diet period and will be clearly instructed on safe food storage and reheating. They will be required to complete dietary compliance sheet on a daily basis. Instructions on dietary compliance will be given at each study period and the dietary compliance records will be reviewed regularly during the intervention to assess their compliance. For the first one week prior to the intervention (one-week standardization), and during the four-week washout period, participants will be asked to avoid eating probiotics, yogurt and cultured milk drink; if any, to standardize their gut microbiota and avoid any carry-over effects as shown in previous studies[56]. Meal-timing will be standardised among all participants as much as possible during the intervention as well as during the standardisation period.

For peer review only

Data Collection and Analyses of outcome measures:

The timing and frequency of study measurements are depicted in Figure 1. Participants will be asked to come fasted (a minimum of 8 hours) for each laboratory visit (pre and post each intervention period) and the following measurements will be made:

1. *Anthropometric measurements:* Participant height (cm), weight (kg), waist circumference (cm), body mass index (kg/m^2) and body composition with bladder voided using Bio-electrical Impedance analysis (BIA - ACC appliance model BIA00A; BioTekna, Marcon VE, Italy).

2. *Liver fat assessment:* Hepatic fat fraction and comparison lipid composition will be measured using a Philips 3T Acheiva MRI scanner with 32 channel receive XL torso coil and advanced lipid composition analysis on a Philips 7T Acheiva scanner with 8 channel multi transmit body coil. MRS will be acquired from a 20x20x20mm voxel within the lower right lobe of the liver using Stimulated Echo Acquisition Mode (STEAM) localization to assess hepatic fat fraction and composition. Spectra will be acquired over four echo times (TE 20, 30, 40, 80) to correct for T_2 relaxation over 5 breath-holds. Water will be used as a reference peak, and water suppression used to analyse signal from lipids. Spectra will be post processed (phase corrected, line-broadened, frequency aligned and averaged) as described previously[57], and then fitted according to known lipid signal positions[58]. T_2 corrected PDFF will be calculated as PDFF = FAT / (FAT+WATER) and converted to true mass fat fraction using known tissue values[59]. Lipid composition will be estimated using individual

For peer review only

peak amplitudes[60].Subcutaneous and visceral fat volumes will be determined using gradient echo Magnetic Resonance (MR) imaging[61].

Liver fat levels will also be assessed with the CAP™ scored using Fibroscan™, which has previously been evaluated against MRS[62] as well as liver biopsy[63] in cross sectional cohorts. Although MRI-PDFF more accurately classifies steatosis[64], portability, accessibility and costs favour CAP™ associated with transient elastography. However, CAP™ has not been evaluated as a marker of efficacy of intervention. For the purpose of validation, participants will undergo a CAP-Fibroscan™ at each intervention visit to quantify their liver fat levels.

4. *Hepatic ATP flux:* Rate of mitochondrial ATP turnover in the liver will be assessed using a Philips 3T MRI scanner with a Philips Phosphorous (31P) single loop surface coil. 31P MRS will be acquired from the liver using a 60mm thick slice to avoid contamination from abdominal muscle. A progressive saturation scan protocol (5 spectra acquired with varying length pre-saturation pulses) will be used to determine apparent T1[65]. Two 31P MR spectra will then be acquired with full saturation of gamma ATP (-2.5ppm) and mirrored downfield of the inorganic phosphate peak (~14ppm) respectively. An in-house fitting program will be used for post-processing spectra and measuring the saturation transfer to the inorganic phosphate peak and used to determine hepatic ATP flux rate constant. ATP concentration will also be determined from spectra and used to calculate final ATP flux rate values[66].

5. *Metabolic Biomarkers:* Blood samples (not more than 80 mL at each visit) will be taken from fasted participants (12-hours fast) for analyses of metabolic analytes

For peer review only

such as: glycaemic, insulinemic, liver, lipid, hematologic profiles, gut hormones (ghrelin, leptin, GLP-1; glucagon-like peptide-1, glucagon) and plasma SCFAs. All biochemical parameters concentrations will be assessed by a central laboratory that uses standard methods. Standard operating procedures (SOPs) will be followed for all laboratory assays to ensure the high quality and reliability of our generated data. Routine blood tests will be analysed by the accredited laboratories. Samples will be stored in -80˚C freezers until the study ends, under yearly service contract and monitoring systems. Analyses will be done according to assay manufacturer advice and completed by technicians and research staff who are formally trained or outsourced to collaborators or external suppliers where necessary. The insulin resistance will be estimated using homeostatic model assessment of insulin resistance (HOMA-IR). HOMA-IR is calculated according to the equation [HOMA-IR = (FPI× FPG)/22.5]; where FPI is fasting plasma insulin, and FPG is fasting plasma glucose[50].

6. *Gut Microbiota Composition*: Each participant will be asked to collect stool sample in a container and bring it to study intervention visit on ice. Deoxyribonucleic acid (DNA) will be first extracted from stool samples for gut microbiota analysis. Then, the gut microbiota composition will be assessed using 16S sequencing of rRNA. DNA will be diluted to 20ng/μl for 16S rRNA amplification and sequencing. Water negative controls will be included from extraction, through polymerase chain reaction (PCR) to sequencing and select samples were sequenced in duplicate for quality control. The V4 region of the 16S rRNA gene will be amplified using universal primers 355F (CCAGACTCCTACGGGAGGCAGC) and 806R (GGACTACHVGGGTWTCTAAT). Amplified DNA will be sequenced on the MiSeq platform (Illumina). Read filtering and

For peer review only

clustering will carried out using the MYcrobiota pipeline56. Briefly, chimeric sequences will be filtered using the VSEARCH algorithm within Mothur, and reads will be clustered into operational taxonomic units (OTUs) using closed-reference clustering against the SILVA database v132 based on a 97% similarity. Diversity metrics (Shannon index, observed OTUs and Unweighted UniFrac) will be calculated by rarefying the OTU table down to 7000 sequences per sample 50 times and taking the average. These analyses will be carried out in QIIME 2 (v2018.11)[67 68].

7. *Subjective Sensation Assessments:* VAS scores will be used to assess the participant's subjective appetite (hunger and fullness) during the intervention. Participants will be provided with a booklet to record their subjective appetite before and after each meal during the intervention at selected days.

Sample Size Calculation and Justification:

The sample size is calculated using nQuery based on previous completed study by Bawden *et al*, (2017)[31]. Based on their pilot data, baseline liver fat fractions of both diet groups were 2.4 ± 1-1.2%, and after 1 week were HGI (High GI) = 4.7 ± 2 % and LGI = 1.6 ± 0.7%. Using a two-group t-test (Crossover ANOVA) between the post-diet values with a 0.05 two-sided significance level, this produces a sample size of n=8 in each diet sequence (a total sample size of 16). This sample size has an 80% power to detect a difference in means of 3.1 (the difference between a Treatment 1, μ_1, of 4.7 and a Treatment 2, μ_2, of 1.6) with the assumption that the Crossover ANOVA sqrt (MSE) is 2.828 (the Standard deviation of differences, σ, is 4). Assuming

For peer review only

that 40% of people would drop out from cross-over study design, it is expected that at least 24 participants would need to be recruited.

The effect from the pilot study by Bawden, *et al*, (2017) pilot study (n=7) was an average 1.6% ± 0.7% decrease in liver fat fraction after following a one-week LGI diet ($p < 0.05$). Given that baseline liver fat fraction was 2.4%, in order to see such change with 80% power ($p < 0.05$) we have doubled the participant numbers to n=16 using nQuery software to detect statistically significant differences[31]. Our group has recently completed a study (ClinicalTrials.gov Identifier: NCT03844165) in which 84 participants with NAFLD followed either a LGI food enriched diet or their normal diet. Patients who changed their diet were found to have a significant decrease in liver fat after 16 weeks (from 20.4% to 18.8%) and had a statistically significant decrease in fasting glucose levels after the diet compared to the control group patients following their normal diet. This provides strong support to our hypothesis and demonstrates that a simple dietary change can have measurable impact on NAFLD symptoms in a short period. In another previous randomized crossover trial, metabolic changes were seen within two-weeks in a dietary intervention with 10 participants[69]. Based on these different approaches, we conclude that a sample size of 16 at the end of the study is sufficient. Assuming a 33% dropout rate we estimate that it will be necessary to enrol n=24 NAFLD participants to achieve n=16 completing the two diet-interventions. This dropout rate has been reported in other dietary interventional studies[70].

Statistical Analysis Plan

Baseline characteristics of participants, outcome measures at baseline (liver fat content, blood biomarkers and gut microbial composition) at each period time-point

For peer review only

will be summarized by diet group using descriptive statistics. The differences between the two interventions (LGI and HGI diet) will be tested by matched-pair Student's t-test. Two-way repeated measures ANOVA test will be used to evaluate the effects of diets and time sequences on liver fat content, as well as on gut microbial composition and blood biomarkers and/or interactions with liver fat content, gut microbial composition and blood biomarkers. Data will be presented as mean± standard deviation and the significance of difference will be set at $p < 0.05$. Any changes in the planned statistical methods will be documented in the trial report. Data input, cleaning and analysis will be conducted using Stata software package. All analyses will be conducted on University of Nottingham computers/laptops that are regularly backed up to University of Nottingham servers.

Microbiome analyses: OTUs with a relative abundance of <0.1% in every sample will be removed, and relative OTU abundances will be inverse normal transformed before further analysis. Associations between response to LGI diet and OTU abundance at genus level will be predicted using a general linear model adjusted for age, gender, and BMI. OTUs will be assumed to be significantly associated with response to the dietary intervention with a value <0.05 after adjusting for false discovery rate (FDR).

DISCUSSION AND RESEARCH IMPLICATIONS:

Building on previous findings from Morgan *et al*[71] and Bawden *et al*,[31], this study will investigate the effects of LGI v HGI diets in participants diagnosed with NAFLD, over a two-week period. Whilst previous studies showed a significant impact of LGI on liver fat reduction and other metabolic biomarkers, questions remain on the effect in

For peer review only

individuals with NAFLD and the longer-term impact. Due to the carefully controlled nature of the intervention, the sample size can be reduced, and the research used to probe more specifically into the direct effects of varying the type of carbohydrate intake. Many studies have shown that energy reduction has a profound effect on liver fat and metabolic health[72], but these diets have been found to be unsustainable in a community setting[18]. Proof of the direct impact of varying a specific characteristic of one macronutrient without reducing the overall intake of the macronutrient consumed, or the overall energy consumed would provide a strong basis for further investigations on the sustainability of such diets.

This study takes advantage of advanced MR protocols which provide a powerful method for non-invasively assessing the effects of lifestyle interventions on metabolic health alongside other physiological measures. Biopsy measurements are uncomfortable for the patient carrying a number of significant risks and would be ethically inappropriate in otherwise healthy individuals. In addition, biopsy only provides a small sample from one region of the liver. MRI and MRS have been well validated[59 73] and allows for repeated tissue specific measurements of fat fraction with minimal risk to study volunteers, thus enabling robust longitudinal studies on the impact of nutrition on metabolic health and liver disease. Additionally, recent research has highlighted the potential significance of lipid composition in metabolic disorders and novel methodologies for measuring this using MRI and MRS have been developed[60]. These advanced techniques will be used to assess not only lipid accumulation but also the effect on saturated, unsaturated, and polyunsaturated fat stores in the liver. Subcutaneous and visceral fat volumes will also be obtained

For peer review only

alongside hepatic fat fraction during the scan session giving a more complete picture of abdominal lipid deposition.

^{31}P MRS measurements of hepatic ATP flux provide a novel and exploratory assessment of the impact of lifestyle interventions on metabolism. Previous studies have shown the correlation of metabolic disorders such as NASH with impaired ATP turnover in the liver[74]. Using advanced in-vivo saturation transfer MR protocols, this study will monitor these effects during an intervention and provide the first in-human study to explore mechanistic effects of diet on liver lipid accumulation. This will give a foundation for future research projects and help to advance the effort against the growing global pandemic of NAFLD and related disorders.

Outcomes from this study will provide evidence on the effects of a LGI diet consumption on measures of NAFLD and will serve as a complementary tool for reducing hepatic fat accumulation in NAFLD patients. It will also be the first to provide nutritional-related information about the effects of LGI diet on gut microbiota composition in this medical condition. Accordingly, trial findings will open the way for academic beneficiaries (including dietitians, hepatologists, diabetologists) to develop a novel strategy for NAFLD monitoring through understanding the mechanism behind the LGI dietary-related effects of specific gut microbiota and hepatic fat accumulation.

In addition, the validation findings for the use of CAP-Fibroscan™ as a new tool for assessing hepatic fat levels in dietary interventions has the potential to enhance diagnostic and research capacity. Furthermore, the validation of Norfolk-EPIC FFQ in Nottingham population will permit its use in larger population-based studies.

For peer review only

If this study successfully reproduces the reduction in hepatic fat levels through LGI diet reported in healthy volunteers[31] in a NAFLD patient cohort, this will be a key step towards developing a new option for dietary intervention. The inclusion of LGI foods as a recommendation for NAFLD patients may have substantial beneficial impacts on society as well. It will first enhance the quality of life, health and well-being of NAFLD patients by reducing the burden of NAFLD progression to advanced liver diseases and more likely to be practical and thriving in the long term as it is the first non-calorie restriction approach. Second, it will contribute towards evidence-based policy making in GI food labelling and therapeutic dietary recommendations and will contribute to increase public awareness of dietary issues. The potential medical costs for a patient with NAFLD per year is between €354 to €1163 in Europe[3]. Thus, preventing NAFLD progression will reduce the burden on health care systems and clinical services as NAFLD patients have high-risk liver-related morbidities and metabolic comorbidities. Also, it may urge the food industries and services to adopt labelling GI values in nutrition facts labels of food products and to reformulate high GI foods to make them lower.

Data from this trial are intended to be presented at local and international conferences attended by the scientific community and relevant health care professionals (e.g., clinicians and dietitians). Within a five-year timeframe the data will be displayed in the trial websites for trial participants. Data will also be used as part of a Philosophy Doctorate thesis. Publications will be in peer reviewed journals. No participant will be identified in any of these publications.

For peer review only

AUTHORS CONTRIBUTIONS

Amina Al-Awadi, Guruprasad Aithal, Jane Grove, Moira Taylor, Ana Valdes, and Stephen Bawden conceived the study and designed the protocol. Moira Taylor and Amina Al-Awadi contributed to diet planning. Stephen Bawden and Penny Gowland involved in the discussion of initial study design and subsequent outline and contributed to the planning of MRI/S data collection and analysis. Amina Al-Awadi drafted the manuscript and all authors revised the manuscript critically.

FUNDING This study is funded by University of Nottingham studentship to Amina Al-Awadi and National Institute for Health Research (NIHR) Nottingham Digestive Diseases Biomedical Research Unit and NIHR Nottingham Biomedical Research Centre [BRC-1215-20003] at the Nottingham University Hospitals NHS Trust and University of Nottingham.

COMPETING INTERESTS None declared

Ethics Approval East Midlands Nottingham 2 Research Ethics Committee (The Old Chapel, Royal Standard Place, Nottingham), REC reference 19/EM/0291.

Acknowledgements:

We thank Lu Ban for her assistance in the calculation of study sample size and acquisition of data. We also thank Andy Wragg and the NIHR Nottingham Biomedical Research Centre (BRC) Digestive Diseases Patient Advisory Group for their support in developing and design of the study. We are also grateful for support from liver and gastrointestinal disorder research team and

For peer review only

for advice from Liz Simpson, Prarthana Thiagarajan, and Sally Cordon. The views expressed are those of the authors and not necessarily those of the NHS, the NIHR or the Department of Health.

Legends: Figure 1: Schematic diagram for crossover trial of LGI diet versus HGI diet effects on liver fat content in participants with NAFLD.

For peer review only

REFERENCES

1. Del Chierico F, Gnani D, Vernocchi P, et al. Meta-omic platforms to assist in the understanding of NAFLD gut microbiota alterations: tools and applications. *Int J Mol Sci* 2014;15(1):684-711. doi: 10.3390/ijms15010684
2. Machado MV, Cortez-Pinto H. Diet, Microbiota, Obesity, and NAFLD: A Dangerous Quartet. *Int J Mol Sci* 2016;17(4):481-81. doi: 10.3390/ijms17040481
3. Younossi ZM, Henry L, Bush H, et al. Clinical and Economic Burden of Nonalcoholic Fatty Liver Disease and Nonalcoholic Steatohepatitis. *Clinics in Liver Disease* 2018;22(1):1-10. doi: https://doi.org/10.1016/j.cld.2017.08.001
4. Conlon BA, Beasley JM, Aebersold K, et al. Nutritional management of insulin resistance in nonalcoholic fatty liver disease (NAFLD). *Nutrients* 2013;5(10):4093-114. doi: 10.3390/nu5104093 [published Online First: 2013/10/25]
5. Perumpail BJ, Cholankeril R, Yoo ER, et al. An Overview of Dietary Interventions and Strategies to Optimize the Management of Non-Alcoholic Fatty Liver Disease. *Diseases* 2017;5(4) doi: 10.3390/diseases5040023 [published Online First: 2017/10/27]
6. Estes C, Anstee QM, Arias-Loste MT, et al. Modeling NAFLD disease burden in China, France, Germany, Italy, Japan, Spain, United Kingdom, and United States for the period 2016-2030. *J Hepatol* 2018;69(4):896-904. doi: 10.1016/j.jhep.2018.05.036 [published Online First: 2018/06/11]
7. Akbar DH, Kawther AH. Nonalcoholic fatty liver disease in Saudi type 2 diabetic subjects attending a medical outpatient clinic: prevalence and general characteristics. *Diabetes care* 2003;26 12:3351-2.
8. Rinella ME. Nonalcoholic fatty liver disease: a systematic review. *Jama* 2015;313(22):2263-73. doi: 10.1001/jama.2015.5370 [published Online First: 2015/06/10]
9. Le MH, Devaki P, Ha NB, et al. Prevalence of non-alcoholic fatty liver disease and risk factors for advanced fibrosis and mortality in the United States. *PLoS One* 2017;12(3):e0173499-e99. doi: 10.1371/journal.pone.0173499
10. Roberts EA. Pediatric nonalcoholic fatty liver disease (NAFLD): a "growing" problem? *J Hepatol* 2007;46(6):1133-42. doi: 10.1016/j.jhep.2007.03.003 [published Online First: 2007/04/21]
11. Wesolowski SR, Kasmi KCE, Jonscher KR, et al. Developmental origins of NAFLD: a womb with a clue. *Nature Reviews Gastroenterology & Hepatology* 2017;14(2):81-96. doi: 10.1038/nrgastro.2016.160
12. Zaki M, Yousef W, Kamal S, et al. Association Between Metabolic Abnormalities and Non-Alcoholic Fatty Liver in Obese Premenopausal Women. *Biomedical and Pharmacology Journal* 2018;11(2):1161-66.
13. Lucas C, Lucas G, Lucas N, et al. A systematic review of the present and future of non-alcoholic fatty liver disease. *Clinical and Experimental Hepatology* 2018;4(3):165.
14. Kim D, Touros A, Kim WR. Nonalcoholic fatty liver disease and metabolic syndrome. *Clinics in liver disease* 2018;22(1):133-40.
15. Buzzetti E, Pinzani M, Tsochatzis EA. The multiple-hit pathogenesis of non-alcoholic fatty liver disease (NAFLD). *Metabolism* 2016;65(8):1038-48.
16. Dongiovanni P, Lanti C, Riso P, et al. Nutritional therapy for nonalcoholic fatty liver disease. *The Journal of nutritional biochemistry* 2016;29:1-11.

For peer review only

17. Duarte SMB, Faintuch J, Stefano JT, et al. Hypocaloric high-protein diet improves clinical and biochemical markers in patients with nonalcoholic fatty liver disease (NAFLD). *Nutricion hospitalaria* 2014;29(1):94-101.
18. Musso G, Cassader M, Rosina F, et al. Impact of current treatments on liver disease, glucose metabolism and cardiovascular risk in non-alcoholic fatty liver disease (NAFLD): a systematic review and meta-analysis of randomised trials. *Diabetologia* 2012;55(4):885-904.
19. Vos MB. Nutrition, nonalcoholic fatty liver disease and the microbiome: recent progress in the field. *Current opinion in lipidology* 2014;25(1):61.
20. McCarthy EM, Rinella ME. The role of diet and nutrient composition in nonalcoholic fatty liver disease. *Journal of the Academy of Nutrition and Dietetics* 2012;112(3):401-09.
21. Jenkins D, Wolever T, Jenkins A, et al. The glycaemic index of foods tested in diabetic patients: a new basis for carbohydrate exchange favouring the use of legumes. *Diabetologia* 1983;24(4):257-64.
22. Association CD. 2008 clinical practice guidelines for the prevention and management of diabetes in Canada. 2008
23. Parker A, Kim Y. The Effect of Low Glycemic Index and Glycemic Load Diets on Hepatic Fat Mass, Insulin Resistance, and Blood Lipid Panels in Individuals with Nonalcoholic Fatty Liver Disease. *Metabolic syndrome and related disorders* 2019;17(8):389-96.
24. Kaur B, Ranawana V, Teh A-L, et al. The impact of a low glycemic index (GI) breakfast and snack on daily blood glucose profiles and food intake in young Chinese adult males. *Journal of clinical & translational endocrinology* 2015;2(3):92-98.
25. Bolsinger J, Landstrom M, Pronczuk A, et al. Low glycemic load diets protect against metabolic syndrome and Type 2 diabetes mellitus in the male Nile rat. *The Journal of nutritional biochemistry* 2017;42:134-48.
26. Isken F, Klaus S, Petzke K-J, et al. Impairment of fat oxidation under high-vs. low-glycemic index diet occurs before the development of an obese phenotype. *American Journal of Physiology-Endocrinology and Metabolism* 2010;298(2):E287-E95.
27. Rivera I. Impact of a low fructose, low glycemic index and low glycemic load dietary intervention on liver function, body composition and cardio metabolic risk factors in children and adolescents with nonalcoholic fatty liver disease. 2013
28. Finelli C, Tarantino G. Is there any consensus as to what diet or lifestyle approach is the right one for NAFLD patients. *J Gastrointestin Liver Dis* 2012;21(3):293-302.
29. Freidoony L, Kong ID. Practical approaches to the nutritional management of nonalcoholic fatty liver disease. *Integrative Medicine Research* 2014;3(4):192-97.
30. Valtuena S, Pellegrini N, Ardigo D, et al. Dietary glycemic index and liver steatosis. *The American journal of clinical nutrition* 2006;84(1):136-42.
31. Bawden S, Stephenson M, Falcone Y, et al. Increased liver fat and glycogen stores after consumption of high versus low glycaemic index food: A randomized crossover study. *Diabetes, Obesity and Metabolism* 2017;19(1):70-77.
32. Tilg H, Kaser A. Gut microbiome, obesity, and metabolic dysfunction. *The Journal of clinical investigation* 2011;121(6):2126-32.
33. Albenberg LG, Wu GD. Diet and the intestinal microbiome: associations, functions, and implications for health and disease. *Gastroenterology* 2014;146(6):1564-72.
34. Carmody RN, Gerber GK, Luevano Jr JM, et al. Diet dominates host genotype in shaping the murine gut microbiota. *Cell host & microbe* 2015;17(1):72-84.
35. Voreades N, Kozil A, Weir TL. Diet and the development of the human intestinal microbiome. *Frontiers in microbiology* 2014;5:494.

For peer review only

36. David LA, Maurice CF, Carmody RN, et al. Diet rapidly and reproducibly alters the human gut microbiome. *Nature* 2014;505(7484):559-63.
37. Singh RK, Chang H-W, Yan D, et al. Influence of diet on the gut microbiome and implications for human health. *Journal of translational medicine* 2017;15(1):73.
38. Power SE, O'Toole PW, Stanton C, et al. Intestinal microbiota, diet and health. *British Journal of Nutrition* 2014;111(3):387-402.
39. Wu GD, Chen J, Hoffmann C, et al. Linking long-term dietary patterns with gut microbial enterotypes. *Science* 2011;334(6052):105-08.
40. Tremaroli V, Bäckhed F. Functional interactions between the gut microbiota and host metabolism. *Nature* 2012;489(7415):242-49.
41. Salonen A, Vos WMd. Impact of Diet on Human Intestinal Microbiota and Health. *Annual Review of Food Science and Technology* 2014;5(1):239-62. doi: 10.1146/annurev-food-030212-182554
42. Bäckhed F, Ding H, Wang T, et al. The gut microbiota as an environmental factor that regulates fat storage. *Proceedings of the national academy of sciences* 2004;101(44):15718-23.
43. Cani PD, Delzenne NM. The role of the gut microbiota in energy metabolism and metabolic disease. *Curr Pharm Des* 2009;15(13):1546-58. doi: 10.2174/138161209788168164 [published Online First: 2009/05/16]
44. D'Aversa F, Tortora A, Ianiro G, et al. Gut microbiota and metabolic syndrome. *Intern Emerg Med* 2013;8 Suppl 1:S11-5. doi: 10.1007/s11739-013-0916-z [published Online First: 2013/03/08]
45. Egshatyan L, Kashtanova D, Popenko A, et al. Gut microbiota and diet in patients with different glucose tolerance. *Endocr Connect* 2016;5(1):1-9. doi: 10.1530/EC-15-0094 [published Online First: 2015/11/10]
46. Kau AL, Ahern PP, Griffin NW, et al. Human nutrition, the gut microbiome and the immune system. *Nature* 2011;474(7351):327-36. doi: 10.1038/nature10213 [published Online First: 2011/06/17]
47. Aron-Wisnewsky J, Gaborit B, Dutour A, et al. Gut microbiota and non-alcoholic fatty liver disease: new insights. *Clinical Microbiology and Infection* 2013;19(4):338-48.
48. Machado MV, Cortez-Pinto H. Diet, microbiota, obesity, and NAFLD: a dangerous quartet. *Int J Mol Sci* 2016;17(4):481.
49. Miele L, Marrone G, Lauritano C, et al. Gut-liver axis and microbiota in NAFLD: insight pathophysiology for novel therapeutic target. *Current Pharmaceutical Design* 2013;19(29):5314-24.
50. Caussy C, Alquiraish MH, Nguyen P, et al. Optimal threshold of controlled attenuation parameter with MRI-PDFF as the gold standard for the detection of hepatic steatosis. *Hepatology* 2018;67(4):1348-59.
51. McKeown NM, Day NE, Welch AA, et al. Use of biological markers to validate self-reported dietary intake in a random sample of the European Prospective Investigation into Cancer United Kingdom Norfolk cohort. *Am J Clin Nutr* 2001;74(2):188-96. doi: 10.1093/ajcn/74.2.188 [published Online First: 2001/07/27]
52. International Physical Activity Questionnaire. 2002, August [Available from: https://sites.google.com/site/theipaq/.
53. Henry C. Basal metabolic rate studies in humans: measurement and development of new equations. *Public health nutrition* 2005;8(7a):1133-52.
54. Dietary reference values for food energy and nutrients for the United Kingdom. Report of the Panel on Dietary Reference Values of the Committee on Medical Aspects of Food

For peer review only

Policy. *Rep Health Soc Subj (Lond)* 1991;41:1-210. [published Online First: 1991/01/01]

55. Henry CJK, Lightowler HJ, Strik CM, et al. Glycaemic index and glycaemic load values of commercially available products in the UK. *British journal of nutrition* 2005;94(6):922-30.
56. Mano F, Ikeda K, Joo E, et al. The effect of white rice and white bread as staple foods on gut microbiota and host metabolism. *nutrients* 2018;10(9):1323.
57. Stephenson M, Leverton E, Khoo E, et al. Variability in fasting lipid and glycogen contents in hepatic and skeletal muscle tissue in subjects with and without type 2 diabetes: a 1H and 13C MRS study. *NMR in Biomedicine* 2013;26(11):1518-26.
58. Hamilton G, Middleton MS, Bydder M, et al. Effect of PRESS and STEAM sequences on magnetic resonance spectroscopic liver fat quantification. *Journal of Magnetic Resonance Imaging: An Official Journal of the International Society for Magnetic Resonance in Medicine* 2009;30(1):145-52.
59. Szczepaniak LS, Babcock EE, Schick F, et al. Measurement of intracellular triglyceride stores by H spectroscopy: validation in vivo. *American Journal of Physiology-Endocrinology And Metabolism* 1999;276(5):E977-E89.
60. Johnson NA, Walton DW, Sachinwalla T, et al. Noninvasive assessment of hepatic lipid composition: advancing understanding and management of fatty liver disorders. *Hepatology* 2008;47(5):1513-23.
61. Bydder M, Yokoo T, Hamilton G, et al. Relaxation effects in the quantification of fat using gradient echo imaging. *Magnetic resonance imaging* 2008;26(3):347-59.
62. Karlas T, Petroff D, Garnov N, et al. Non-invasive assessment of hepatic steatosis in patients with NAFLD using controlled attenuation parameter and 1 H-MR spectroscopy. *PLoS One* 2014;9(3):e91987.
63. Eddowes PJ, Sasso M, Allison M, et al. Accuracy of FibroScan controlled attenuation parameter and liver stiffness measurement in assessing steatosis and fibrosis in patients with nonalcoholic fatty liver disease. *Gastroenterology* 2019;156(6):1717-30.
64. Imajo K, Honda Y, Kessoku T, et al. Magnetic Resonance Imaging More Accurately Classifies Steatosis and Liver Fibrosis in Non-Alcoholic Fatty Liver Disease than Transient Elastography. *Journal of Hepatology* 2016;64(2):S175-S76.
65. Chen C, Stephenson MC, Peters A, et al. 31P magnetization transfer magnetic resonance spectroscopy: Assessing the activation induced change in cerebral ATP metabolic rates at 3 T. *Magnetic Resonance in Medicine* 2018;79(1):22-30. doi: 10.1002/mrm.26663
66. Valkovič L, Chmelík M, Krššák M. In-vivo(31)P-MRS of skeletal muscle and liver: A way for non-invasive assessment of their metabolism. *Anal Biochem* 2017;529:193-215. doi: 10.1016/j.ab.2017.01.018 [published Online First: 2017/01/26]
67. Fernandes G, Tap J, Bruls T, et al. Enterotypes of the human gut microbiome. *Nature* 2011;473(7346):174180.
68. D'Argenio V, Salvatore F. The role of the gut microbiome in the healthy adult status. *Clinica chimica acta* 2015;451:97-102.
69. Farshchi HR, Taylor MA, Macdonald IA. Beneficial metabolic effects of regular meal frequency on dietary thermogenesis, insulin sensitivity, and fasting lipid profiles in healthy obese women. *The American journal of clinical nutrition* 2005;81(1):16-24.
70. Samaha FF, Iqbal N, Seshadri P, et al. A low-carbohydrate as compared with a low-fat diet in severe obesity. *New England Journal of Medicine* 2003;348(21):2074-81.
71. Morgan LM, Shi J-W, Hampton SM, et al. Effect of meal timing and glycaemic index on glucose control and insulin secretion in healthy volunteers. *British journal of nutrition* 2012;108(7):1286-91.

For peer review only - http://bmjopen.bmj.com/site/about/guidelines.xhtml

72. Taylor R. Type 2 diabetes: etiology and reversibility. *Diabetes care* 2013;36(4):1047-55.
73. Reeder SB, Sirlin CB. Quantification of liver fat with magnetic resonance imaging. *Magnetic Resonance Imaging Clinics* 2010;18(3):337-57.
74. Traussnigg S, Kienbacher C, Gajdošík M, et al. Ultra-high-field magnetic resonance spectroscopy in non-alcoholic fatty liver disease: Novel mechanistic and diagnostic insights of energy metabolism in non-alcoholic steatohepatitis and advanced fibrosis. *Liver Int* 2017;37(10):1544-53. doi: 10.1111/liv.13451 [published Online First: 2017/05/20]

For peer review only

Title: Effects of an iso-energetic low glycaemic index (GI) diet on liver fat accumulation and gut microbiota composition in patients with non- alcoholic fatty liver disease (NAFLD): An efficacy mechanism evaluation

Author: Amina Abdullah Al-Awadi[1,2]

Corresponding Author: Guruprasad Aithal[1,2] (Postal Address: E Floor, West Block Queen's Medical Centre Derby Road, Nottingham, NG7 2UH, Tel: 0115 823 1149, Email: guru.aithal@nottingham.ac.uk)

Co-Authors: Jane Grove[1,2], Moira Taylor[3], Ana Valdes[2,5], Amrita Vijay[1,5], Stephen Bawden[4], Penny Gowland[4],

AUTHORS AFFILIATIONS

1. Nottingham Digestive Diseases Centre, University of Nottingham, Nottingham, United Kingdom.

2. NIHR Nottingham Biomedical Research Centre, Nottingham University Hospitals Trust and the University of Nottingham, Nottingham, United Kingdom.

3. Department of Nutrition and Dietetics, School of Life Sciences, University of Nottingham, Nottingham, United Kingdom.

4. Sir Peter Mansfield Imaging Centre, School of Medicine, University of Nottingham, Nottingham, United Kingdom.

5. Division of Rheumatology Orthopaedics and Dermatology, School of Life Sciences, University of Nottingham, Nottingham, United Kingdom.

For peer review only

Figure 1: Schematic diagram for crossover trial of LGI diet versus HGI diet effects on liver fat content in participants with NAFLD.

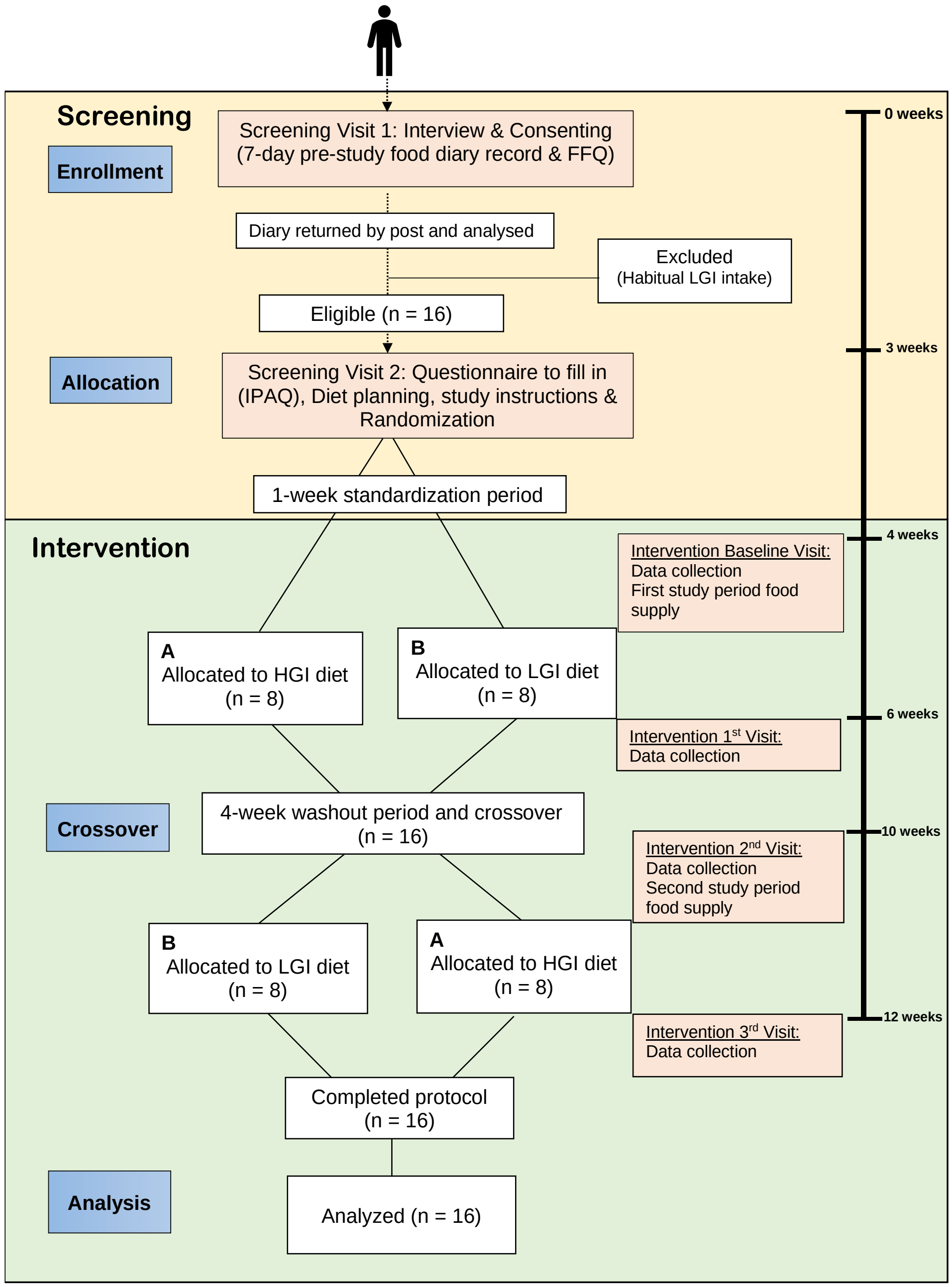

BMJ Open

Effects of an iso-energetic low glycaemic index (GI) diet on liver fat accumulation and gut microbiota composition in patients with non- alcoholic fatty liver disease (NAFLD): An efficacy mechanism evaluation

Journal:	*BMJ Open*
Manuscript ID	bmjopen-2020-045802.R1
Article Type:	Protocol
Date Submitted by the Author:	10-Mar-2021
Complete List of Authors:	Al-Awadi, Amina; University of Nottingham, Nottingham Digestive Diseases Centre<br>Grove, Jane; University of Nottingham, Nottingham Digestive Diseases Centre<br>Taylor, Moira ; University of Nottingham School of Life Sciences,<br>Valdes, A; University of Nottingham,<br>Vijay, Amrita; University of Nottingham, Nottingham Digestive Diseases Centre<br>Bawden, Stephen; University of Nottingham, Physics and Astronomy<br>Gowland, Penny; University of Nottingham, Department of Physics and Astronomy<br>Aithal, Guruprasad; Nottingham Digestive Diseases Biomedical Research Centre, Nottingham Digestive Diseases Centre
**Primary Subject Heading**:	Nutrition and metabolism
Secondary Subject Heading:	Nutrition and metabolism, Diabetes and endocrinology, Gastroenterology and hepatology
Keywords:	Hepatology < INTERNAL MEDICINE, NUTRITION & DIETETICS, Hepatobiliary disease < GASTROENTEROLOGY

SCHOLARONE™
Manuscripts

I, the Submitting Author has the right to grant and does grant on behalf of all authors of the Work (as defined in the below author licence), an exclusive licence and/or a non-exclusive licence for contributions from authors who are: i) UK Crown employees; ii) where BMJ has agreed a CC-BY licence shall apply, and/or iii) in accordance with the terms applicable for US Federal Government officers or employees acting as part of their official duties; on a worldwide, perpetual, irrevocable, royalty-free basis to BMJ Publishing Group Ltd ("BMJ") its licensees and where the relevant Journal is co-owned by BMJ to the co-owners of the Journal, to publish the Work in this journal and any other BMJ products and to exploit all rights, as set out in our licence.

The Submitting Author accepts and understands that any supply made under these terms is made by BMJ to the Submitting Author unless you are acting as an employee on behalf of your employer or a postgraduate student of an affiliated institution which is paying any applicable article publishing charge ("APC") for Open Access articles. Where the Submitting Author wishes to make the Work available on an Open Access basis (and intends to pay the relevant APC), the terms of reuse of such Open Access shall be governed by a Creative Commons licence – details of these licences and which Creative Commons licence will apply to this Work are set out in our licence referred to above.

Other than as permitted in any relevant BMJ Author's Self Archiving Policies, I confirm this Work has not been accepted for publication elsewhere, is not being considered for publication elsewhere and does not duplicate material already published. I confirm all authors consent to publication of this Work and authorise the granting of this licence.

For peer review only

Title: Effects of an iso-energetic low glycaemic index (GI) diet on liver fat accumulation and gut microbiota composition in patients with non- alcoholic fatty liver disease (NAFLD): An efficacy mechanism evaluation

Authors: Amina Al-Awadi, Jane Grove, Moira Taylor, Ana Valdes, Amrita Vijay, Stephen Bawden, Penny Gowland, Guruprasad Aithal.

Corresponding Author: Guruprasad P. Aithal**,** Nottingham Digestive Diseases Centre, School of Medicine, University of Nottingham, Nottingham UK, NG7 2UH.
Email address: mszag3@exmail.nottingham.ac.uk
Tel: +44 (0)115 823 1031

AUTHORS AFFILIATIONS

Amina A. Al-Awadi, Nottingham Digestive Diseases Centre, University of Nottingham. NIHR Nottingham Biomedical Research Centre, Nottingham University Hospitals Trust and the University of Nottingham. Nottingham, United Kingdom.

Jane I. Grove, Nottingham Digestive Diseases Centre, University of Nottingham. NIHR Nottingham Biomedical Research Centre, Nottingham University Hospitals Trust and the University of Nottingham. Nottingham, United Kingdom.

Moira A. Taylor, Department of Nutrition and Dietetics, School of Life Sciences, University of Nottingham, Nottingham, United Kingdom.

Ana M. Valdes, NIHR Nottingham Biomedical Research Centre, Nottingham University Hospitals Trust and the University of Nottingham. Division of Rheumatology Orthopaedics and Dermatology, School of Life Sciences, University of Nottingham. Nottingham, United Kingdom

Amrita Vijay, Nottingham Digestive Diseases Centre, University of Nottingham. Division of Rheumatology Orthopaedics and Dermatology, School of Life Sciences, University of Nottingham. Nottingham, United Kingdom.

Stephen J. Bawden, Sir Peter Mansfield Imaging Centre, School of Medicine, University of Nottingham, Nottingham, United Kingdom.

Penny A. Gowland, Sir Peter Mansfield Imaging Centre, School of Medicine, University of Nottingham, Nottingham, United Kingdom.

Guruprasad P. Aithal Nottingham Digestive Diseases Centre, University of Nottingham. NIHR Nottingham Biomedical Research Centre, Nottingham University Hospitals Trust and the University of Nottingham. Nottingham, United Kingdom.

Key words: Nutrition and Dietetics, Hepatology, Hepatobiliary disease, Fatty liver, glycaemic index.
Word count: 5234

For peer review only

ABSTRACT:

Introduction A low glycaemic index (LGI) diet is a proposed lifestyle intervention in non-alcoholic fatty liver diseases (NAFLD) which is designed to reduce circulating blood glucose levels, hepatic glucose influx, insulin resistance, and *de novo* lipogenesis. A significant reduction in liver fat content through following a one-week LGI diet has been reported in healthy volunteers. Changes in dietary fat and carbohydrates have also been shown to alter gut microbiota composition and lead to hepatic steatosis through the gut-liver axis. There are no available trials examining the effects of a LGI diet on liver fat accumulation in patients with NAFLD; nor has the impact of consuming a LGI diet on gut microbiota composition been studied in this population. The aim of this trial is to investigate the effects of LGI diet consumption on liver fat content and its effects on gut microbiota composition in participants with NAFLD compared with a high glycaemic index (HGI) control diet.

Methods and Analysis A 2x2 crossover randomized mechanistic dietary trial will allocate 16 participants with NAFLD to a 2-week either HGI or LGI diet followed by a 4-week wash-out period and then the LGI or HGI diet, alternative to that followed in the first 2-weeks. Baseline and post-intervention (4 visits) outcome measures will be collected to assess liver fat content (using MRI/S and CAP-Fibroscan™), gut microbiota composition (using 16S RNA analysis) and blood biomarkers including glycaemic, insulinemic, liver, lipid and hematologic profiles, gut hormones levels, and short chain fatty acids (SCFAs).

Ethics and Dissemination Study protocol has been approved by the ethics committees of The University of Nottingham and East Midlands Nottingham-2

For peer review

For peer review only - http://bmjopen.bmj.com/site/about/guidelines.xhtml

Research Ethics Committee (REC reference 19/EM/0291). Data from this trial will be used as part of a Philosophy Doctorate thesis. Publications will be in peer reviewed journals.

Trial registration number (NCT04415632, protocol v1.1, 28/10/2019)

For peer review only

For peer review only - http://bmjopen.bmj.com/site/about/guidelines.xhtml

ARTICLE SUMMARY:

Strengths

- A carefully controlled human LGI diet intervention to reduce liver fat in NAFLD using cross-over study deign.
- The study will assess associations between LGI dietary change and gut microbiota, and between the change in microbiota and liver fat levels.
- The first NAFLD patient study that explores the mechanistic effects of diet on liver fat accumulation using advanced MR protocols.
- Uses the controlled attenuation parameter (CAP™) as a biomarker to evaluate efficacy of diet intervention.

Limitations:

- Findings from small sample size may not be generalizable to all patients with NAFLD so will require replication in larger long-term randomised controlled trial.

For peer review only

INTRODUCTION

Non-alcoholic fatty liver disease (NAFLD) is a growing global health concern increasing in parallel with the worsening epidemics of obesity and diabetes[1 2]. Recent reviews indicated that one billion people worldwide have NAFLD (estimated prevalence 25.4%)[3]. NAFLD is characterized by the fat accumulation in hepatocytes exceeding 5% of liver weight and encompasses a spectrum of disease severities. Common clinical risk factors that are highly associated with NAFLD include obesity, insulin resistance, type 2 diabetes, and metabolic syndrome[3 4]. NAFLD is recognized as the hepatic manifestation of metabolic syndrome[5-7].

Western dietary habits occurring concurrently with a sedentary lifestyle and genetic factors are associated with insulin resistance, adipocyte proliferation and gut microbiota alteration which may be implicated in NAFLD[8]. Dietary habits, hormones secreted from the adipose tissue, gut microbiota composition and genetic factors are all implicated in the pathogenesis.

To date, there are no drugs with regulatory approval for treating NAFLD, and lifestyle changes including modification of dietary patterns together with increased physical activity is the initial step to treat this disease[9]. Dietary approaches beneficial in NAFLD management include reductions in saturated and trans fatty acids, total carbohydrates, and animal-based protein intake and increases in the intake of polyunsaturated fatty acids (PUFAs), monounsaturated fatty acids (MUFAs), plant- based proteins, and antioxidants[4]. Furthermore, evidence from a systematic review and meta-analysis of randomized control trials (RCT) on patients with NAFLD has shown that lifestyle-induced weight loss of ≥ 7% improved histological disease activity, as well as

For peer review only - http://bmjopen.bmj.com/site/about/guidelines.xhtml

cardiometabolic risk profile, in less than 50% of the study population (n=373 patients)[10]. A recent systematic review has concluded that Despite the abundance of interventional studies on dietary management in NAFLD, there is still a lack of evidence-based specific recommendations, other than that weight loss may benefit some; and few studies have measured liver-related outcomes[11]. Moreover, the dietary effects on liver-related outcomes that are mediated by gut microbiome have not been studied in these patients[12].

More recently, the glycaemic index (GI) and glycaemic load (GL), of diet; has been proposed as an important predictor of NAFLD progression[4 13]. The GI of a food ranks the glycaemic response under conditions in which the quantity of carbohydrate is controlled[14 15], whereas the GL additionally considers portion size[16].

NAFLD patients have been found to consume a diet high in HGI foods[4] which are correlated with insulin resistance, metabolic dysfunction[4], and liver dysfunction[17]. In addition, HGI dietary habits have been significantly associated with high-grade liver steatosis in patients with insulin resistance[18] while limiting HGI foods is beneficial in NAFLD[19 20]. Further, previous pilot study showed that only one-week of consuming HGI diet caused significant increase in hepatic stores of fat when compared with isocaloric LGI diet consumption[21]. A recent review concluded that both low GI and GL diets resulted in significant reductions in hepatic fat mass in a cohort of 269 participants[16]. However, these effects were not attributed to the LGI diet alone and were confounded with other lifestyle interventions[16].

Evidence from animal and human studies also showed that diet is the most important modifying factor in the development of human gut microbiome [22-24]. Dietary alterations

For peer review only - http://bmjopen.bmj.com/site/about/guidelines.xhtml

can rapidly change the human gut microbiota composition in relatively short time periods[25-27] and in as little as 24 hours[28-30]. Certain dietary factors which modify gut microbiota have been associated with a number of diseases including obesity[31] metabolic syndrome[22 32 33], type 2 diabetes[34], immunological dysfunctions[35]. Such dysbiosis in diet-induced NAFLD has been shown to stimulate hepatic fat disposition and promote NAFLD progression to non-alcoholic steatohepatitis (NASH)[36 37]. This is suggested to be mediated by modulation of gut permeability, dietary choline metabolism, bile acid metabolism, endogenous ethanol production and immune balance [36 37]. Gut microbiota modulated by diet can increase the energy extraction from the breakdown of non-absorbable polysaccharides with the production of monosaccharides and short chain fatty acids (SCFAs) which are substrates for hepatic *de novo* lipogenesis and carbohydrate biosynthesis[38]. Certain SCFAs have also been implicated in improving glucose and lipid metabolism beneficial to the host[39]. Therefore, there is a potential to modulate gut microbiota through dietary changes as a means of NAFLD prevention or alleviation[11 40].

We describe here our study protocol designed to assess the effects of a specific defined GI dietary manipulation[21] on liver fat content and its effects on gut microbiota composition in participants with NAFLD compared with a higher GI diet as a control diet. The diets will be adjusted to each participant to meet their energy requirements and the macronutrient composition of the diets will be carefully controlled ensuring that differences in GL are due to differences in GI.

For peer review only - http://bmjopen.bmj.com/site/about/guidelines.xhtml

OBJECTIVES

Primary Objective

1. To investigate the effects of defined LGI versus HGI diet on liver fat accumulation in participants with NAFLD following a two-week intervention period whilst controlling for the energy consumption and amount of carbohydrate intake.

Secondary Objectives

1. To investigate the effects of LGI versus HGI diet on gut microbiota composition and its correlations with liver outcomes in participants with NAFLD following a two-week intervention period.
2. To investigate the changes in blood metabolic biomarkers (glycaemic, insulinemic, liver, lipid, hematologic profiles, gut hormones, and plasma SCFAs) between LGI and HGI diet groups following a two-week intervention in participants with NAFLD.
3. To evaluate CAP™ as a tool to test the efficacy of a dietary intervention by comparing its performance characteristics with that of Magnetic Resonance Spectroscopy (MRS) as a gold standard.
4. To validate the European Prospective Investigation of Cancer food frequency questionnaire (EPIC-FFQ) in Nottingham NAFLD patients against food diary.

For peer review only - http://bmjopen.bmj.com/site/about/guidelines.xhtml

METHODS AND ANALYSIS

Trial Design

This is a randomized 2 x 2 cross-over mechanistic trial including two arms, each of two weeks duration comparing a LGI diet and a control HGI diet adapted from Bawden et al. (2017)[21] and Morgan et al. (2012)[41]. The study design is illustrated in Figure 1.

Public and patient involvement

We collaborated with the NIHR Nottingham BRC patient advisory group, and we met 7 patients who were interested in study participation. They expressed strong support for the research objectives and the recent meeting the group considered the one-week crossover intervention feasible and acceptable. Patient feedback informed the participant recruitment through information content and delivery methods. They also assisted in the study design to enhance participant compliance and commitment to the diet.

Participants and Recruitment

We aim to recruit 16 participants with NAFLD to complete both phases of 2x2 cross-over trial. The initial screening criteria for inclusion is a CAP™ score of >288dB/m; this value correlates with Magnetic Resonance Imaging (MRI)- Proton Density Fat Fraction (PDFF) estimation of > 5% fat of liver weight which indicates that they have a fatty liver[42].

NAFLD patients will be recruited either from the existing database of “The Scarred Liver Project” in the UK who have given consent to be contacted to participate in future research (approved by East Midlands-Leicester Research ethics committee ref: B/EM/0123) or from liver clinics at the Nottingham University Hospitals NHS Trust who had a Fibroscan with CAP or participants from public (advert in local and social media).

For peer review only - http://bmjopen.bmj.com/site/about/guidelines.xhtml

The eligibility criteria are summarized in Table 1. All participants will provide a written informed consent by trained research team before they enter the trial. Potential participants will be free to withdraw at any time. In the event of their withdrawal, it will be explained that their data collected so far cannot be erased and we will seek consent to use the data in the final analyses where appropriate. In the event of a participant withdrawing or being unable to continue the intervention periods after randomisation, a replacement will be recruited who will be assigned to the same diet group to ensure a 1:1 ratio for the 2 diet groups. We will continue recruiting until 16 participants have fully completed the study.

**Table 1: Eligibility Criteria**
Inclusion criteria<br>• Adult males and females aged from 18 to 65 years (balanced number).<br>• Detected NAFLD by CAP-FibroScan™ >288dB/m or by MRI-PDFF) > 5% fat of liver weight.<br>• Body mass index ≥ 25 kg/m^2<br>• Abdominal obesity (Waist circumference > 102 cm for males and > 88 cm for females)<br>• Have current moderate to high GI diet intake of ≥ 60 (Assessed from a completed 7-day food diary).<br>• Able to give informed consent.<br>• Able to undergo MRI/S and CAP-FibroScan™.
Exclusion criteria<br>• Current smokers and excessive alcohol drinkers (> 14 units/week).<br>• Perimenopausal (irregular periods) women.<br>• Participants with other liver abnormalities.<br>• Participants with history of gastrointestinal surgeries, depression, eating disorders or difficulties.<br>• Participants using pharmacologic agents for obesity or NAFLD.<br>• Participants with type 1 diabetes.<br>• Participants with type 2 diabetes on second line medications (eg GLP-1 analogues).<br>• Participation in any other trial in the last 3 months.<br>• Participants on any special diets (e.g. vegetarians).<br>• Intolerance to foods included in the diet plan.

For peer review only

Screening process:

Participants will be invited to attend an initial screening visit (Figure 1). At this visit, participants will be consented and asked to complete a pre-screening questionnaire (including taking height, weight, and blood pressure measurements) to establish whether they meet the study criteria. Participants will be asked to fill out a 7-day food records and post it after one week of their initial screening visit. This is to avoid giving patients with NAFLD, who might be at risk of developing diabetes, a much higher HGI diet than they habitually consume. Using 7-day food records, we will characterise the baseline habitual GI diet intake of all potential volunteers at screening period. Participants also will be required to complete a food frequency questionnaire (FFQ) for validation against the 7-day food records. We will use EPIC-FFQ that was validated for use in Norfolk cohort[43]. After completion, participant's eligibility will be reassessed based on analysis of diet information using Nutritics software (Nutritics Ltd, Dublin, Ireland, 2018). Those with a habitual LGI diet intake (GI < 60) will be excluded from the study. Only patients who have a moderate to HGI diet intake (GI ≥ 60) will be invited to attend the next screening preparation visit.

Then at next screening visit (visit 2), the participants will be asked to fill out International Physical Activity Questionnaire (IPAQ)[44]. A trained dietitian will instruct participants on how to measure study food provided using measuring jug and spoons and how to fill in study diet compliance sheets. They will be instructed on stool collection and pre-intervention visits requirements. Participants will be asked to avoid eating probiotics and/or prebiotics supplements or drinks for one week before the intervention period (standardization week). All instructions will be provided to each participant in a form of Home Guide Booklet.

For peer review only - http://bmjopen.bmj.com/site/about/guidelines.xhtml

Each eligible participant will be assigned randomly to undergo either HGI diet (HGI group) or LGI group first and then crossover to either HGI or LGI diets.

Randomisation and Blinding

Enrolled participants who meet the eligibility criteria will be randomized to one of two groups which will dictate the order in which they receive the LGI or HGI diet. They will then receive the second diet depending upon which diet they have first received. The random allocation sequence (AB or BA) will be generated by one of the trial co-ordinators; who will have no contact with the participants, using block randomization by impartial biostatistics. The online randomisation package (http://randomization.com) will be used to allocate the sequences of the diet groups to the participants in equal numbers. Once the random number is generated, both trial investigators and participants are aware of the diet sequence (i.e., whether the participant is going to start with LGI diet; A or HGI diet; B). The diets then will be delivered to the participant according to the sequence. The trial outcome measures will be determined by blinded researcher using codes on samples that will be sent for 16S ribosomal ribonucleic acid (rRNA), blood marker and MRI/CAP-FibroScan™ analyses. The blinded allocation will be provided to the study statistician at the end of the study and once the analysis has been completed, the study statistician will be provided with the un-blinded allocation.

Study protocol:

Following the screening phase and standardization week, each participant will experience two, two-week intervention phases, with a four-week washout period between interventions. Before and after each intervention period they will attend the

For peer review only - http://bmjopen.bmj.com/site/about/guidelines.xhtml

Imaging Centre (Sir Peter Mansfield Imaging Centre) for MRI scan measurements and then will attend the Biomedical Research Centre for Fibroscan, and blood and stool samples collection. During each intervention phase each participant will be required to consume all the food that has been provided, fill in dietary compliance sheets and complete visual analogue scales (VAS) relating to subjective appetite ratings.

Design of the dietary interventions.

Energy expenditure will be estimated for each participant using the individual's basal metabolic rate (BMR) calculated from Henry-modified Schofield formula[45] and multiplied by an activity factor based on the IPAQ score physical activity levels (PALs). A menu, consisting of meals for 3 days personalised for energy requirement, will be devised (these meals are repeated over 2 weeks) by adjusting a template according to their estimated energy requirement in order to minimise weight change during the intervention phase. The diet consists of normal food closely resembling that described by Bawden *et al*, (2017) study[21]. A diet template of 2000 kcal/day with energy proportions distributed as 55% from carbohydrates, 15% from protein and 30% from fat[46] are designed and food quantities will be modified to supply the energy requirements for each participant. Three-day cycle menu plans are designed for each diet (LGI/ HGI). Table 2 shows example template menus for both HGI (a) and LGI (b) diets.

For peer review only - http://bmjopen.bmj.com/site/about/guidelines.xhtml

Table 2 Illustration of HGI (a) and LGI (b) diet templates for use in the study.

HGI Diet Sample (a)

	Weight (g)	Energy (Kcal)	Carbohydrates (g)	Protein (g)	Fat (g)	Fibre (g)	GI	GL
Breakfast								
Sultana Bran Cereal	90	268	58	6.6	1.3	10.1	90	52.2
Skimmed Milk	180	63	8.3	6.3	0.54	0	48	4
Roasted Peanuts	15	90	2.3	3.7	7.4	1.3	14	0.32
Glucose	10	40	10	0	0	0	100	10
Water	170	0	0	0	0	0	0	0
Meal Total		461	78	16.6	9.2	11.4	85.3	66.5
Lunch								
Margarine	25	102	0.13	0.05	11.3	0	0	0
Fruit loaf	144	395	76	11.4	5.2	4.6	90	68.4
Roasted turkey slice	42	47	0.67	10	0.5	0.12	0	0
Cheddar cheese slice	25	104	0.03	6.4	8.7	0	0	0
Strawberry yogurt	125	88	16.3	5.6	0	0.38	85	13.855
Meal Total		735	93	33.4	25.7	5.1	88.44	82.255
Dinner								
Cottage Pie (ready meal)	400	441	44	18	21.6	4.8	66	29.04
Low fat Strawberry yogurt	125	88	16.3	5.6	0	0.38	85	13.855
Water	170	0	0	0	0	0	0	0
Glucose	10	40	10	0	0	0	100	10
Mars bar	58	237	37	2.4	8.9	0.87	65	24.05
Meal Total		806	107	26	30.5	6	72.69	76.954
Menu Total		2002 Kcal	278g (55.5%)	76g (15.2%)	65g (29.3%)	22g	82.1	225.706

LGI Diet Sample (b)

	Weight (g)	Energy (Kcal)	Carbohydrates (g)	Protein (g)	Fat (g)	Fibre (g)	GI	GL
Breakfast								
All Bran Cereal	90	239	42	11.2	3.1	22.1	43	18.06
Semi-Skimmed Milk	160	76	7.2	5.6	2.7	0	25	1.8
Orange juice	250	96	22	1.5	0.25	0	53	11.66
Meal Total		410	71	18.2	6	22.1	44.39	31.52
Lunch								
Pumpernickel bread	180	408	74	15.7	5.6	11.7	41	30.34
Black cherry yogurt	150	209	24.2	4.8	10.4	0.75	17	4.114
Soft cheese	51	129	1.5	2.7	12.4	0	34	0.51
Meal Total		745	99	23.2	28.4	12.5	35.32	34.96
Dinner								
Lasagne (ready meal)	290	413	42	21.5	17.7	4.9	36	15.12
Apple	174	92	20	1	0.87	2.1	38	7.6
Semi-skimmed milk	150	71	6.8	5.3	2.6	0	25	1.7
Fruit &nuts mix	60	267	24.6	8.4	15	3.9	15	3.69
Meal Total		843	93	36.2	36.1	10.9	30.22	28.11
Menu Total		1999 Kcal	264g (52.7%)	78g (15.5%)	71g (31.8%)	46g	36.64	94.594

For peer review only - http://bmjopen.bmj.com/site/about/guidelines.xhtml

The GI values of commercial food items are generated from that reported by Henry *et al*. (2005)[47], as well as Nutritics software databases (Nutritics Ltd, Dublin, Ireland, 2018). The dietary GI of the menu is calculated from GI proportional contribution to total carbohydrate using the equation: $GI = \sum(C_{food}/C_{total}) \times GI_{food}$; where C_{food} is the amount of carbohydrate contained in each digested food (g), C_{total} is the total carbohydrate in the meal and GI_{food} is the reported GI of the food[21]. The GL of the diet is calculated based on the dietary GI and the amount of carbohydrate provided (the same in both diets) using the following equation: $GL = (GI_{food} \times C_{food}) / 100$; where GI_{food} is the GI of each digested food and C_{food} is the amount of carbohydrate in the food[21]. Food composition analysis and evaluation were performed using Nutritics software (Nutritics Ltd, Dublin, Ireland, 2018).

Participants will be provided with the food at the start of each two-week diet period and will be clearly instructed on safe food storage and reheating. They will be required to complete dietary compliance sheet on a daily basis. Instructions on dietary compliance will be given at each study period and the dietary compliance records will be reviewed regularly during the intervention to assess their compliance. For the first one week prior to the intervention (one-week standardization), and during the four-week washout period, participants will be asked to avoid eating probiotics, yogurt and cultured milk drink; if any, to standardize their gut microbiota and avoid any carry-over effects as shown in previous studies[48 49]. Meal-timing will be standardised among all participants as much as possible during the intervention as well as during the standardisation period.

For peer review only - http://bmjopen.bmj.com/site/about/guidelines.xhtml

Data Collection and Analyses of outcome measures:

The timing and frequency of study measurements are depicted in Figure 1. Participants will be asked to come fasted (a minimum of 8 hours) for each laboratory visit (pre and post each intervention period) and the following measurements will be made:

1. *Anthropometric measurements:* Participant height (cm), weight (kg), waist circumference (cm), body mass index (kg/m^2) and body composition with bladder voided using Bio-electrical Impedance analysis (BIA - ACC appliance model BIA00A; BioTekna, Marcon VE, Italy).

2. *Liver fat assessment:* Hepatic fat fraction and comparison lipid composition will be measured using a Philips 3T Acheiva MRI scanner with 32 channel receive XL torso coil and advanced lipid composition analysis on a Philips 7T Acheiva scanner with 8 channel multi transmit body coil. MRS will be acquired from a 20x20x20mm voxel within the lower right lobe of the liver using Stimulated Echo Acquisition Mode (STEAM) localization to assess hepatic fat fraction and composition. Spectra will be acquired over four echo times (TE 20, 30, 40, 80) to correct for T_2 relaxation over 5 breath-holds. Water will be used as a reference peak, and water suppression used to analyse signal from lipids. Spectra will be post processed (phase corrected, line-broadened, frequency aligned and averaged) as described previously[50], and then fitted according to known lipid signal positions[51]. T_2 corrected PDFF will be calculated as PDFF = FAT / (FAT+WATER) and converted to true mass fat fraction using known tissue values[52]. Lipid composition will be estimated using individual

For peer review only - http://bmjopen.bmj.com/site/about/guidelines.xhtml

peak amplitudes[53].Subcutaneous and visceral fat volumes will be determined using gradient echo Magnetic Resonance (MR) imaging[54].

Liver fat levels will also be assessed with the CAP™ scored using Fibroscan™, which has previously been evaluated against MRS[55] as well as liver biopsy[56] in cross sectional cohorts. Although MRI-PDFF more accurately classifies steatosis[57], portability, accessibility and costs favour CAP™ associated with transient elastography. However, CAP™ has not been evaluated as a marker of efficacy of intervention. For the purpose of validation, participants will undergo a CAP-Fibroscan™ at each intervention visit to quantify their liver fat levels.

3. *Hepatic ATP flux:* Rate of mitochondrial ATP turnover in the liver will be assessed using a Philips 3T MRI scanner with a Philips Phosphorous (31P) single loop surface coil. 31P MRS will be acquired from the liver using a 60mm thick slice to avoid contamination from abdominal muscle. A progressive saturation scan protocol (5 spectra acquired with varying length pre-saturation pulses) will be used to determine apparent T1[58]. Two 31P MR spectra will then be acquired with full saturation of gamma ATP (-2.5ppm) and mirrored downfield of the inorganic phosphate peak (~14ppm) respectively. An in-house fitting program will be used for post-processing spectra and measuring the saturation transfer to the inorganic phosphate peak and used to determine hepatic ATP flux rate constant. ATP concentration will also be determined from spectra and used to calculate final ATP flux rate values[59].

4. *Metabolic Biomarkers:* Blood samples (not more than 80 mL at each visit) will be taken from fasted participants (12-hours fast) for analyses of metabolic analytes

For peer review only - http://bmjopen.bmj.com/site/about/guidelines.xhtml

such as: glycaemic, insulinemic, liver, lipid, hematologic profiles, gut hormones (ghrelin, leptin, glucagon-like peptide-1 (GLP-1), glucagon) and plasma SCFAs. Although GLP-1 levels increase in response to meal ingestion, levels can be directly affected by presence of SCFAs, produced from fermentation of certain carbohydrates which may have been recently consumed, confounding the results. Instead, only fasting levels will be measured as short-term diets containing different carbohydrates types and different GI content have been found to affect fasting GLP-1 levels[60]. In addition, circulating plasma SCFAs levels will be measured as it has been shown to be strongly related to metabolic health[61]. Measurement of plasma SCFAs levels also overcomes the challenges associated with accurate quantification of volatile SCFAs molecules in fecal samples[62].

All biochemical parameters concentrations will be assessed by a central laboratory that uses standard methods. Standard operating procedures (SOPs) will be followed for all laboratory assays to ensure the high quality and reliability of our generated data. Routine blood tests will be analysed by the accredited laboratories. Samples will be stored in -80˚C freezers until the study ends, under yearly service contract and monitoring systems. Analyses will be done according to assay manufacturer advice and completed by technicians and research staff who are formally trained or outsourced to collaborators or external suppliers where necessary. The insulin resistance will be estimated using homeostatic model assessment of insulin resistance (HOMA-IR). HOMA-IR is calculated according to the equation [HOMA-IR = (FPI× FPG)/22.5]; where FPI is fasting plasma insulin, and FPG is fasting plasma glucose[42].

For peer review only - http://bmjopen.bmj.com/site/about/guidelines.xhtml

6. *Gut Microbiota Composition*: Each participant will be asked to collect fresh stool sample in a standard laboratory container and bring it to the research centre with an ice pack for storage at -80°C. Stool samples will be processed using a standard commercial kit for Deoxyribonucleic acid (DNA)extraction[63]. Then, the gut microbiota composition will be assessed using 16S sequencing of rRNA. DNA will be diluted to 20ng/µl for 16S rRNA amplification and sequencing. Water negative controls will be included from extraction, through polymerase chain reaction (PCR) to sequencing and select samples were sequenced in duplicate for quality control. The V4 region of the 16S rRNA gene will be amplified using universal primers 355F (CCAGACTCCTACGGGAGGCAGC) and 806R (GGACTACHVGGGTWTCTAAT). Amplified DNA will be sequenced on the MiSeq platform (Illumina). Read filtering and clustering will carried out using the MYcrobiota pipeline56. Briefly, chimeric sequences will be filtered using the VSEARCH algorithm within Mothur, and reads will be clustered into operational taxonomic units (OTUs) using closed-reference clustering against the SILVA database v132 based on a 97% similarity. Diversity metrics (Shannon index, observed OTUs and Unweighted UniFrac) will be calculated by rarefying the OTU table down to 7000 sequences per sample 50 times and taking the average. These analyses will be carried out in QIIME 2 (v2018.11)[64 65].

7. *Subjective Sensation Assessments:* VAS scores will be used to assess the participant's subjective appetite (hunger and fullness) during the intervention. Participants will be provided with a booklet to record their subjective appetite before and after each meal during the intervention at selected days.

For peer review only - http://bmjopen.bmj.com/site/about/guidelines.xhtml

For peer review only

Sample Size Calculation and Justification:

The sample size is calculated using nQuery based on previous completed study by Bawden *et al*, (2017)[21]. Based on their pilot data, baseline liver fat fractions of both diet groups were 2.4 ± 1-1.2%, and after 1 week were HGI (High GI) = 4.7 ± 2 % and LGI = 1.6 ± 0.7%. Using a two-group t-test (Crossover ANOVA) between the post-diet values with a 0.05 two-sided significance level, this produces a sample size of n=8 in each diet sequence (a total sample size of 16). This sample size has an 80% power to detect a difference in means of 3.1 (the difference between a Treatment 1, μ_1, of 4.7 and a Treatment 2, μ_2, of 1.6) with the assumption that the Crossover ANOVA sqrt (MSE) is 2.828 (the Standard deviation of differences, σ, is 4). Assuming that 40% of people would drop out from cross-over study design, it is expected that at least 24 participants would need to be recruited.

The effect from the pilot study by Bawden, *et al*, (2017) pilot study (n=7) was an average 1.6% ± 0.7% decrease in liver fat fraction after following a one-week LGI diet ($p < 0.05$). Given that baseline liver fat fraction was 2.4%, in order to see such change with 80% power ($p< 0.05$) we have doubled the participant numbers to n=16 using nQuery software to detect statistically significant differences[21]. Our group has recently completed a study (ClinicalTrials.gov Identifier: NCT03844165) in which 84 participants with NAFLD followed either a LGI food enriched diet or their normal diet. Patients who changed their diet were found to have a significant decrease in liver fat after 16 weeks (from 20.4% to 18.8%) and had a statistically significant decrease in fasting glucose levels after the diet compared to the control group patients following their normal diet. This provides strong support to our hypothesis and demonstrates that a simple dietary change can have measurable impact on NAFLD symptoms in a

For peer review only - http://bmjopen.bmj.com/site/about/guidelines.xhtml

short period. In another previous randomized crossover trial, metabolic changes were seen within two-weeks in a dietary intervention with 10 participants[66]. Based on these different approaches, we conclude that a sample size of 16 at the end of the study is sufficient. Assuming a 33% dropout rate we estimate that it will be necessary to enrol n=24 NAFLD participants to achieve n=16 completing the two diet-interventions. This dropout rate has been reported in other dietary interventional studies[67].

Statistical Analysis Plan

Baseline characteristics of participants, outcome measures at baseline (liver fat content, blood biomarkers and gut microbial composition) at each period time-point will be summarized by diet group using descriptive statistics. The differences between the two interventions (LGI and HGI diet) will be tested by matched-pair Student's t-test. Two-way repeated measures ANOVA test will be used to evaluate the effects of diets and time sequences on liver fat content, as well as on gut microbial composition and blood biomarkers and/or interactions with liver fat content, gut microbial composition and blood biomarkers. Data will be presented as mean± standard deviation and the significance of difference will be set at $p < 0.05$. Any changes in the planned statistical methods will be documented in the trial report. Data input, cleaning and analysis will be conducted using Stata software package. All analyses will be conducted on University of Nottingham computers/laptops that are regularly backed up to University of Nottingham servers.

Microbiome analyses: OTUs with a relative abundance of <0.1% in every sample will be removed, and relative OTU abundances will be inverse normal transformed before further analysis. Associations between response to LGI diet and OTU abundance at

For peer review only - http://bmjopen.bmj.com/site/about/guidelines.xhtml

genus level will be predicted using a general linear model adjusted for age, gender, and BMI. OTUs will be assumed to be significantly associated with response to the dietary intervention with a value <0.05 after adjusting for false discovery rate (FDR).

DATA MANAGEMENT

Trial data will be managed by study coordinator (AA) under the supervision of chief investigator (GA) and study statistician (LB). The study coordinator shall carry out monitoring of trial data as an ongoing activity and perform annual site system audit. Trial data and evidence of monitoring and systems audits will be made available for inspection by REC as required. The Sponsor has the right to take advice from the Trial Steering Committee and Data Monitoring Committee as appropriate for study discontinuation. All trial staff and investigators will endeavour to protect the rights of the trial's participants to privacy and informed consent, and will adhere to the Data Protection Act, 2018. Access to the data will be limited to the trial staff and investigators and relevant regulatory authorities and will be restricted by user identifiers and passwords (using a one-way encryption method). Computer held data including the trial database will be held securely and password protected. All data will be stored on a secure dedicated web server. Electronic data will be backed up every 24 hours to both local and remote media in encrypted format.

In compliance with the ICH/GCP guidelines, regulations and in accordance with the University of Nottingham Research Code of Conduct and Research Ethics, the Chief or local Principal Investigator will maintain all records and documents regarding the conduct of the study. These will be retained for at least 7 years or for longer if required. The Trial Master File and trial documents held by the Chief Investigator on behalf of

For peer review only - http://bmjopen.bmj.com/site/about/guidelines.xhtml

the Sponsor shall be finally archived at secure archive facilities at the University of Nottingham. This archive shall include all trial databases and associated meta-data encryption codes.

Ethics and dissemination

Ethical approval was first obtained from the University of Nottingham, Medical School Research Ethics Committee. Then additional ethical approval was obtained from East Midlands Nottingham 2 Research Ethics Committee (The Old Chapel, Royal Standard Place, Nottingham). The trial is registered within ClinicalTrials.gov (NCT04415632, protocol v1.1, 28/10/2019). Within a five-year timeframe, the final anonymised trial data will be uploaded to the https://rdmc.nottingham.ac.uk public repository after consistency and quality have been verified by the project team and publication of the results. Data from this trial are intended to be presented at local and international conferences including those attended by clinicians and dietitians and will also be used as part of a Philosophy Doctorate thesis. Publications will be in peer reviewed journals. No participant will be identified in any of these publications.

For peer review only

DISCUSSION AND RESEARCH IMPLICATIONS:

Building on previous findings from Morgan *et al*[41] and Bawden *et al*,[21], this study will investigate the effects of LGI versus HGI diets in participants diagnosed with NAFLD, over a two-week period. Whilst previous studies showed a significant impact of LGI on liver fat reduction and other metabolic biomarkers, questions remain on the effect in individuals with NAFLD and the longer-term impact. Due to the carefully controlled nature of the intervention, the sample size can be reduced, and the research used to

For peer review only - http://bmjopen.bmj.com/site/about/guidelines.xhtml

probe more specifically into the direct effects of varying the type of carbohydrate intake. Many studies have shown that energy reduction has a profound effect on liver fat and metabolic health[68], but these diets have been found to be unsustainable in a community setting[10]. Proof of the direct impact of varying a specific characteristic of one macronutrient without reducing the overall intake of the macronutrient consumed, or the overall energy consumed would provide a strong basis for further investigations on the sustainability of such diets.

This study takes advantage of advanced MR protocols which provide a powerful method for non-invasively assessing the effects of lifestyle interventions on metabolic health alongside other physiological measures. Biopsy measurements are uncomfortable for the patient carrying a number of significant risks and would be ethically inappropriate in otherwise healthy individuals. In addition, biopsy only provides a small sample from one region of the liver. MRI and MRS have been well validated[52 69] and allows for repeated tissue specific measurements of fat fraction with minimal risk to study volunteers, thus enabling robust longitudinal studies on the impact of nutrition on metabolic health and liver disease. Additionally, recent research has highlighted the potential significance of lipid composition in metabolic disorders and novel methodologies for measuring this using MRI and MRS have been developed[53]. These advanced techniques will be used to assess not only lipid accumulation but also the effect on saturated, unsaturated, and polyunsaturated fat stores in the liver. Subcutaneous and visceral fat volumes will also be obtained alongside hepatic fat fraction during the scan session giving a more complete picture of abdominal lipid deposition.

For peer review only - http://bmjopen.bmj.com/site/about/guidelines.xhtml

^{31}P MRS measurements of hepatic ATP flux provides a novel and exploratory assessment of the impact of lifestyle interventions on metabolism. Previous studies have shown the correlation of metabolic disorders such as NASH with impaired ATP turnover in the liver[70]. Using advanced in-vivo saturation transfer MR protocols, this study will monitor these effects during an intervention and provide the first in-human study to explore mechanistic effects of diet on liver lipid accumulation. This will give a foundation for future research projects and help to advance the effort against the growing global pandemic of NAFLD and related disorders.

Outcomes from this study will provide evidence on the effects of a LGI diet consumption on measures of NAFLD and will serve as a complementary tool for reducing hepatic fat accumulation in NAFLD patients. It will also be the first to provide nutritional-related information about the effects of LGI diet on gut microbiota composition in this medical condition. Accordingly, trial findings will open the way for academic beneficiaries (including dietitians, hepatologists, diabetologists) to develop a novel strategy for NAFLD monitoring through understanding the mechanism behind the LGI dietary-related effects of specific gut microbiota and hepatic fat accumulation.

In addition, the validation findings for the use of CAP-Fibroscan™ as a new tool for assessing hepatic fat levels in dietary interventions has the potential to enhance diagnostic and research capacity. Furthermore, the validation of Norfolk-EPIC FFQ in Nottingham population will permit its use in larger population-based studies.

If this study successfully reproduces the reduction in hepatic fat levels through LGI diet reported in healthy volunteers[21] in a NAFLD patient cohort, this will be a key step towards developing a new option for dietary intervention. The inclusion of LGI foods

For peer review only - http://bmjopen.bmj.com/site/about/guidelines.xhtml

as a recommendation for NAFLD patients may have substantial beneficial impacts on society as well. It will first enhance the quality of life, health and well-being of NAFLD patients by reducing the burden of NAFLD progression to advanced liver diseases and more likely to be practical and thriving in the long term as it is the first non-calorie restriction approach. Second, it will contribute towards evidence-based policy making in GI food labelling and therapeutic dietary recommendations and will contribute to increase public awareness of dietary issues. The potential medical costs for a patient with NAFLD per year is between €354 to €1163 in Europe[3]. Thus, preventing NAFLD progression will reduce the burden on health care systems and clinical services as NAFLD patients have high-risk liver-related morbidities and metabolic comorbidities. Also, it may urge the food industries and services to adopt labelling GI values in nutrition facts labels of food products and to reformulate high GI foods to make them lower.

For peer review only

For peer review only - http://bmjopen.bmj.com/site/about/guidelines.xhtml

AUTHORS CONTRIBUTIONS

Amina Al-Awadi, Guruprasad Aithal, Jane Grove, Moira Taylor, Ana Valdes, and Stephen Bawden conceived the study and designed the protocol. Moira Taylor and Amina Al-Awadi contributed to diet planning. Stephen Bawden and Penny Gowland involved in the discussion of initial study design and subsequent outline and contributed to the planning of MRI/S data collection and analysis. Amina Al-Awadi drafted the manuscript and all authors revised the manuscript critically.

FUNDING This study is funded by University of Nottingham studentship to Amina Al-Awadi and National Institute for Health Research (NIHR) Nottingham Digestive Diseases Biomedical Research Unit and NIHR Nottingham Biomedical Research Centre [BRC-1215-20003] at the Nottingham University Hospitals NHS Trust and University of Nottingham. The study sponsor and funder will have no role in the design, collection, management, analysis, and interpretation of data; writing of the report; and the decision to submit the report for publication. The study sponsor and funder will have no ultimate authority over any of these activities.

COMPETING INTERESTS None declared

Ethics Approval East Midlands Nottingham 2 Research Ethics Committee (The Old Chapel, Royal Standard Place, Nottingham), REC reference 19/EM/0291.

Acknowledgements:

We thank Lu Ban for her assistance in the calculation of study sample size and acquisition of data. We also thank Andy Wragg and the NIHR Nottingham Biomedical Research Centre (BRC) Digestive Diseases Patient Advisory Group

For peer review only

for their support in developing and design of the study. We are also grateful for support from liver and gastrointestinal disorder research team and for advice from Liz Simpson, Prarthana Thiagarajan, and Sally Cordon. The views expressed are those of the authors and not necessarily those of the NHS, the NIHR or the Department of Health.

Legends: Figure 1: Schematic diagram for crossover trial of LGI diet versus HGI diet effects on liver fat content in participants with NAFLD.

For peer review only

For peer review only - http://bmjopen.bmj.com/site/about/guidelines.xhtml

REFERENCES

1. Del Chierico F, Gnani D, Vernocchi P, et al. Meta-omic platforms to assist in the understanding of NAFLD gut microbiota alterations: tools and applications. *Int J Mol Sci* 2014;15(1):684-711. doi: 10.3390/ijms15010684
2. Machado MV, Cortez-Pinto H. Diet, Microbiota, Obesity, and NAFLD: A Dangerous Quartet. *Int J Mol Sci* 2016;17(4):481-81. doi: 10.3390/ijms17040481
3. Younossi ZM, Henry L, Bush H, et al. Clinical and Economic Burden of Nonalcoholic Fatty Liver Disease and Nonalcoholic Steatohepatitis. *Clinics in Liver Disease* 2018;22(1):1-10. doi: https://doi.org/10.1016/j.cld.2017.08.001
4. Perumpail BJ, Cholankeril R, Yoo ER, et al. An Overview of Dietary Interventions and Strategies to Optimize the Management of Non-Alcoholic Fatty Liver Disease. *Diseases* 2017;5(4) doi: 10.3390/diseases5040023 [published Online First: 2017/10/27]
5. Zaki M, Yousef W, Kamal S, et al. Association Between Metabolic Abnormalities and Non-Alcoholic Fatty Liver in Obese Premenopausal Women. *Biomedical and Pharmacology Journal* 2018;11(2):1161-66.
6. Lucas C, Lucas G, Lucas N, et al. A systematic review of the present and future of non-alcoholic fatty liver disease. *Clinical and Experimental Hepatology* 2018;4(3):165.
7. Kim D, Touros A, Kim WR. Nonalcoholic fatty liver disease and metabolic syndrome. *Clinics in liver disease* 2018;22(1):133-40.
8. Buzzetti E, Pinzani M, Tsochatzis EA. The multiple-hit pathogenesis of non-alcoholic fatty liver disease (NAFLD). *Metabolism* 2016;65(8):1038-48.
9. Dongiovanni P, Lanti C, Riso P, et al. Nutritional therapy for nonalcoholic fatty liver disease. *The Journal of nutritional biochemistry* 2016;29:1-11.
10. Musso G, Cassader M, Rosina F, et al. Impact of current treatments on liver disease, glucose metabolism and cardiovascular risk in non-alcoholic fatty liver disease (NAFLD): a systematic review and meta-analysis of randomised trials. *Diabetologia* 2012;55(4):885-904.
11. Conlon BA, Beasley JM, Aebersold K, et al. Nutritional management of insulin resistance in nonalcoholic fatty liver disease (NAFLD). *Nutrients* 2013;5(10):4093-114. doi: 10.3390/nu5104093 [published Online First: 2013/10/25]
12. Vos MB. Nutrition, nonalcoholic fatty liver disease and the microbiome: recent progress in the field. *Current opinion in lipidology* 2014;25(1):61.
13. McCarthy EM, Rinella ME. The role of diet and nutrient composition in nonalcoholic fatty liver disease. *Journal of the Academy of Nutrition and Dietetics* 2012;112(3):401-09.
14. Jenkins D, Wolever T, Jenkins A, et al. The glycaemic index of foods tested in diabetic patients: a new basis for carbohydrate exchange favouring the use of legumes. *Diabetologia* 1983;24(4):257-64.
15. Thompson D, Capes S, Feig D, et al. Canadian Diabetes Association 2008 clinical practice guidelines for the prevention and management of diabetes in Canada. *Can J Diabetes* 2008;32:S168-S80.
16. Parker A, Kim Y. The Effect of Low Glycemic Index and Glycemic Load Diets on Hepatic Fat Mass, Insulin Resistance, and Blood Lipid Panels in Individuals with Nonalcoholic Fatty Liver Disease. *Metabolic syndrome and related disorders* 2019;17(8):389-96.
17. Rivera I. Impact of a low fructose, low glycemic index and low glycemic load dietary intervention on liver function, body composition and cardio metabolic risk factors in children and adolescents with nonalcoholic fatty liver disease. 2013
18. Valtuena S, Pellegrini N, Ardigo D, et al. Dietary glycemic index and liver steatosis. *The American journal of clinical nutrition* 2006;84(1):136-42.

For peer review only

19. Finelli C, Tarantino G. Is there any consensus as to what diet or lifestyle approach is the right one for NAFLD patients. *J Gastrointestin Liver Dis* 2012;21(3):293-302.
20. Freidoony L, Kong ID. Practical approaches to the nutritional management of nonalcoholic fatty liver disease. *Integrative Medicine Research* 2014;3(4):192-97.
21. Bawden S, Stephenson M, Falcone Y, et al. Increased liver fat and glycogen stores after consumption of high versus low glycaemic index food: A randomized crossover study. *Diabetes, Obesity and Metabolism* 2017;19(1):70-77.
22. Tilg H, Kaser A. Gut microbiome, obesity, and metabolic dysfunction. *The Journal of clinical investigation* 2011;121(6):2126-32.
23. Albenberg LG, Wu GD. Diet and the intestinal microbiome: associations, functions, and implications for health and disease. *Gastroenterology* 2014;146(6):1564-72.
24. Carmody RN, Gerber GK, Luevano Jr JM, et al. Diet dominates host genotype in shaping the murine gut microbiota. *Cell host & microbe* 2015;17(1):72-84.
25. Deehan EC, Yang C, Perez-Muñoz ME, et al. Precision Microbiome Modulation with Discrete Dietary Fiber Structures Directs Short-Chain Fatty Acid Production. *Cell Host Microbe* 2020;27(3):389-404.e6. doi: 10.1016/j.chom.2020.01.006 [published Online First: 2020/02/01]
26. Valdes AM, Walter J, Segal E, et al. Role of the gut microbiota in nutrition and health. *Bmj* 2018;361:k2179. doi: 10.1136/bmj.k2179 [published Online First: 2018/06/15]
27. Wu GD, Compher C, Chen EZ, et al. Comparative metabolomics in vegans and omnivores reveal constraints on diet-dependent gut microbiota metabolite production. *Gut* 2016;65(1):63-72. doi: 10.1136/gutjnl-2014-308209 [published Online First: 2014/11/29]
28. Voreades N, Kozil A, Weir TL. Diet and the development of the human intestinal microbiome. *Frontiers in microbiology* 2014;5:494.
29. David LA, Maurice CF, Carmody RN, et al. Diet rapidly and reproducibly alters the human gut microbiome. *Nature* 2014;505(7484):559-63.
30. Singh RK, Chang H-W, Yan D, et al. Influence of diet on the gut microbiome and implications for human health. *Journal of translational medicine* 2017;15(1):73.
31. Bäckhed F, Ding H, Wang T, et al. The gut microbiota as an environmental factor that regulates fat storage. *Proceedings of the national academy of sciences* 2004;101(44):15718-23.
32. Cani PD, Delzenne NM. The role of the gut microbiota in energy metabolism and metabolic disease. *Curr Pharm Des* 2009;15(13):1546-58. doi: 10.2174/138161209788168164 [published Online First: 2009/05/16]
33. D'Aversa F, Tortora A, Ianiro G, et al. Gut microbiota and metabolic syndrome. *Intern Emerg Med* 2013;8 Suppl 1:S11-5. doi: 10.1007/s11739-013-0916-z [published Online First: 2013/03/08]
34. Egshatyan L, Kashtanova D, Popenko A, et al. Gut microbiota and diet in patients with different glucose tolerance. *Endocr Connect* 2016;5(1):1-9. doi: 10.1530/EC-15-0094 [published Online First: 2015/11/10]
35. Kau AL, Ahern PP, Griffin NW, et al. Human nutrition, the gut microbiome and the immune system. *Nature* 2011;474(7351):327-36. doi: 10.1038/nature10213 [published Online First: 2011/06/17]
36. Aron-Wisnewsky J, Gaborit B, Dutour A, et al. Gut microbiota and non-alcoholic fatty liver disease: new insights. *Clinical Microbiology and Infection* 2013;19(4):338-48.
37. Machado MV, Cortez-Pinto H. Diet, microbiota, obesity, and NAFLD: a dangerous quartet. *Int J Mol Sci* 2016;17(4):481.

For peer review only - http://bmjopen.bmj.com/site/about/guidelines.xhtml

38. Miele L, Marrone G, Lauritano C, et al. Gut-liver axis and microbiota in NAFLD: insight pathophysiology for novel therapeutic target. *Current Pharmaceutical Design* 2013;19(29):5314-24.
39. Koh A, De Vadder F, Kovatcheva-Datchary P, et al. From Dietary Fiber to Host Physiology: Short-Chain Fatty Acids as Key Bacterial Metabolites. *Cell* 2016;165(6):1332-45. doi: 10.1016/j.cell.2016.05.041 [published Online First: 2016/06/04]
40. Wesolowski SR, Kasmi KCE, Jonscher KR, et al. Developmental origins of NAFLD: a womb with a clue. *Nature Reviews Gastroenterology & Hepatology* 2017;14(2):81-96. doi: 10.1038/nrgastro.2016.160
41. Morgan LM, Shi J-W, Hampton SM, et al. Effect of meal timing and glycaemic index on glucose control and insulin secretion in healthy volunteers. *British journal of nutrition* 2012;108(7):1286-91.
42. Caussy C, Alquiraish MH, Nguyen P, et al. Optimal threshold of controlled attenuation parameter with MRI-PDFF as the gold standard for the detection of hepatic steatosis. *Hepatology* 2018;67(4):1348-59.
43. McKeown NM, Day NE, Welch AA, et al. Use of biological markers to validate self-reported dietary intake in a random sample of the European Prospective Investigation into Cancer United Kingdom Norfolk cohort. *Am J Clin Nutr* 2001;74(2):188-96. doi: 10.1093/ajcn/74.2.188 [published Online First: 2001/07/27]
44. International Physical Activity Questionnaire. 2002, August [Available from: https://sites.google.com/site/theipaq/.
45. Henry C. Basal metabolic rate studies in humans: measurement and development of new equations. *Public health nutrition* 2005;8(7a):1133-52.
46. Dietary reference values for food energy and nutrients for the United Kingdom. Report of the Panel on Dietary Reference Values of the Committee on Medical Aspects of Food Policy. *Rep Health Soc Subj (Lond)* 1991;41:1-210. [published Online First: 1991/01/01]
47. Henry CJK, Lightowler HJ, Strik CM, et al. Glycaemic index and glycaemic load values of commercially available products in the UK. *British journal of nutrition* 2005;94(6):922-30.
48. Mano F, Ikeda K, Joo E, et al. The effect of white rice and white bread as staple foods on gut microbiota and host metabolism. *nutrients* 2018;10(9):1323.
49. Farup PG, Jacobsen M, Ligaarden SC, et al. Probiotics, symptoms, and gut microbiota: what are the relations? A randomized controlled trial in subjects with irritable bowel syndrome. *Gastroenterol Res Pract* 2012;2012:214102. doi: 10.1155/2012/214102 [published Online First: 2012/08/18]
50. Stephenson M, Leverton E, Khoo E, et al. Variability in fasting lipid and glycogen contents in hepatic and skeletal muscle tissue in subjects with and without type 2 diabetes: a 1H and 13C MRS study. *NMR in Biomedicine* 2013;26(11):1518-26.
51. Hamilton G, Middleton MS, Bydder M, et al. Effect of PRESS and STEAM sequences on magnetic resonance spectroscopic liver fat quantification. *Journal of Magnetic Resonance Imaging: An Official Journal of the International Society for Magnetic Resonance in Medicine* 2009;30(1):145-52.
52. Szczepaniak LS, Babcock EE, Schick F, et al. Measurement of intracellular triglyceride stores by H spectroscopy: validation in vivo. *American Journal of Physiology-Endocrinology And Metabolism* 1999;276(5):E977-E89.

For peer review only - http://bmjopen.bmj.com/site/about/guidelines.xhtml

53. Johnson NA, Walton DW, Sachinwalla T, et al. Noninvasive assessment of hepatic lipid composition: advancing understanding and management of fatty liver disorders. *Hepatology* 2008;47(5):1513-23.
54. Bydder M, Yokoo T, Hamilton G, et al. Relaxation effects in the quantification of fat using gradient echo imaging. *Magnetic resonance imaging* 2008;26(3):347-59.
55. Karlas T, Petroff D, Garnov N, et al. Non-invasive assessment of hepatic steatosis in patients with NAFLD using controlled attenuation parameter and 1 H-MR spectroscopy. *PLoS One* 2014;9(3):e91987.
56. Eddowes PJ, Sasso M, Allison M, et al. Accuracy of FibroScan controlled attenuation parameter and liver stiffness measurement in assessing steatosis and fibrosis in patients with nonalcoholic fatty liver disease. *Gastroenterology* 2019;156(6):1717-30.
57. Imajo K, Honda Y, Kessoku T, et al. Magnetic Resonance Imaging More Accurately Classifies Steatosis and Liver Fibrosis in Non-Alcoholic Fatty Liver Disease than Transient Elastography. *Journal of Hepatology* 2016;64(2):S175-S76.
58. Chen C, Stephenson MC, Peters A, et al. 31P magnetization transfer magnetic resonance spectroscopy: Assessing the activation induced change in cerebral ATP metabolic rates at 3 T. *Magnetic Resonance in Medicine* 2018;79(1):22-30. doi: 10.1002/mrm.26663
59. Valkovič L, Chmelík M, Krššák M. In-vivo(31)P-MRS of skeletal muscle and liver: A way for non-invasive assessment of their metabolism. *Anal Biochem* 2017;529:193-215. doi: 10.1016/j.ab.2017.01.018 [published Online First: 2017/01/26]
60. Keller J, Kahlhöfer J, Peter A, et al. Effects of Low versus High Glycemic Index Sugar-Sweetened Beverages on Postprandial Vasodilatation and Inactivity-Induced Impairment of Glucose Metabolism in Healthy Men. *Nutrients* 2016;8(12):802. doi: 10.3390/nu8120802
61. Müller M, Hernández MAG, Goossens GH, et al. Circulating but not faecal short-chain fatty acids are related to insulin sensitivity, lipolysis and GLP-1 concentrations in humans. *Scientific Reports* 2019;9(1):12515. doi: 10.1038/s41598-019-48775-0
62. Liebisch G, Ecker J, Roth S, et al. Quantification of Fecal Short Chain Fatty Acids by Liquid Chromatography Tandem Mass Spectrometry-Investigation of Pre-Analytic Stability. *Biomolecules* 2019;9(4) doi: 10.3390/biom9040121 [published Online First: 2019/03/31]
63. Astbury S, Atallah E, Vijay A, et al. Lower gut microbiome diversity and higher abundance of proinflammatory genus Collinsella are associated with biopsy-proven nonalcoholic steatohepatitis. *Gut Microbes* 2020;11(3):569-80. doi: 10.1080/19490976.2019.1681861 [published Online First: 2019/11/08]
64. Fernandes G, Tap J, Bruls T, et al. Enterotypes of the human gut microbiome. *Nature* 2011;473(7346):174180.
65. D'Argenio V, Salvatore F. The role of the gut microbiome in the healthy adult status. *Clinica chimica acta* 2015;451:97-102.
66. Farshchi HR, Taylor MA, Macdonald IA. Beneficial metabolic effects of regular meal frequency on dietary thermogenesis, insulin sensitivity, and fasting lipid profiles in healthy obese women. *The American journal of clinical nutrition* 2005;81(1):16-24.
67. Samaha FF, Iqbal N, Seshadri P, et al. A low-carbohydrate as compared with a low-fat diet in severe obesity. *New England Journal of Medicine* 2003;348(21):2074-81.
68. Taylor R. Type 2 diabetes: etiology and reversibility. *Diabetes care* 2013;36(4):1047-55.
69. Reeder SB, Sirlin CB. Quantification of liver fat with magnetic resonance imaging. *Magnetic Resonance Imaging Clinics* 2010;18(3):337-57.
70. Traussnigg S, Kienbacher C, Gajdošík M, et al. Ultra-high-field magnetic resonance spectroscopy in non-alcoholic fatty liver disease: Novel mechanistic and diagnostic

For peer review only - http://bmjopen.bmj.com/site/about/guidelines.xhtml

insights of energy metabolism in non-alcoholic steatohepatitis and advanced fibrosis. *Liver Int* 2017;37(10):1544-53. doi: 10.1111/liv.13451 [published Online First: 2017/05/20]

For peer review only

For peer review only - http://bmjopen.bmj.com/site/about/guidelines.xhtml

For peer review only

For peer review only - http://bmjopen.bmj.com/site/about/guidelines.xhtml

Title: Effects of an iso-energetic low glycaemic index (GI) diet on liver fat accumulation and gut microbiota composition in patients with non- alcoholic fatty liver disease (NAFLD): An efficacy mechanism evaluation

Author: Amina Abdullah Al-Awadi[1,2]

Corresponding Author: Guruprasad Aithal[1,2] (Postal Address: E Floor, West Block Queen's Medical Centre Derby Road, Nottingham, NG7 2UH, Tel: 0115 823 1149, Email: guru.aithal@nottingham.ac.uk)

Co-Authors: Jane Grove[1,2], Moira Taylor[3], Ana Valdes[2,5], Amrita Vijay[1,5], Stephen Bawden[4], Penny Gowland[4],

AUTHORS AFFILIATIONS

1. Nottingham Digestive Diseases Centre, University of Nottingham, Nottingham, United Kingdom.

2. NIHR Nottingham Biomedical Research Centre, Nottingham University Hospitals Trust and the University of Nottingham, Nottingham, United Kingdom.

3. Department of Nutrition and Dietetics, School of Life Sciences, University of Nottingham, Nottingham, United Kingdom.

4. Sir Peter Mansfield Imaging Centre, School of Medicine, University of Nottingham, Nottingham, United Kingdom.

5. Division of Rheumatology Orthopaedics and Dermatology, School of Life Sciences, University of Nottingham, Nottingham, United Kingdom.

For peer review only

Figure 1: Schematic diagram for crossover trial of LGI diet versus HGI diet effects on liver fat content in participants with NAFLD.

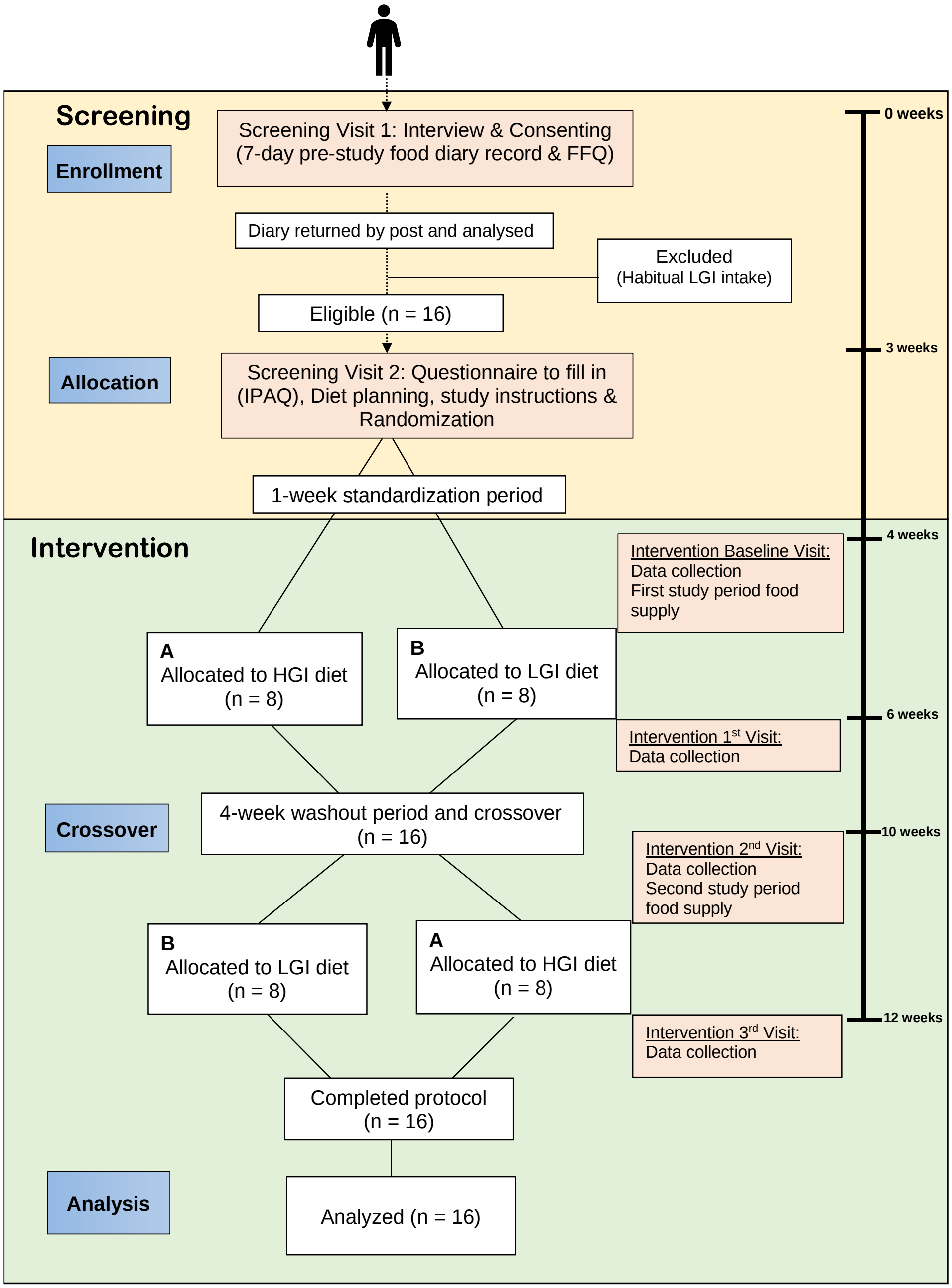

STANDARD PROTOCOL ITEMS: RECOMMENDATIONS FOR INTERVENTIONAL TRIALS

SPIRIT 2013 Checklist: Recommended items to address in a clinical trial protocol and related documents*

Section/item	Item No	Description	Addressed on page number
**Administrative information**			
Title	1	Descriptive title identifying the study design, population, interventions, and, if applicable, trial acronym	___ 1__________
Trial registration	2a	Trial identifier and registry name. If not yet registered, name of intended registry	____3_________ _
	2b	All items from the World Health Organization Trial Registration Data Set	____9,10_______
Protocol version	3	Date and version identifier	__3 &23 ________
Funding	4	Sources and types of financial, material, and other support	__27__________
Roles and responsibilities	5a	Names, affiliations, and roles of protocol contributors	__1 & 27______
	5b	Name and contact information for the trial sponsor	__27_________
	5c	Role of study sponsor and funders, if any, in study design; collection, management, analysis, and interpretation of data; writing of the report; and the decision to submit the report for publication, including whether they will have ultimate authority over any of these activities	__27__________

For peer review only

	5d	Composition, roles, and responsibilities of the coordinating centre, steering committee, endpoint adjudication committee, data management team, and other individuals or groups overseeing the trial, if applicable (see Item 21a for data monitoring committee)	___22,23 ________
**Introduction**			
Background and rationale	6a	Description of research question and justification for undertaking the trial, including summary of relevant studies (published and unpublished) examining benefits and harms for each intervention	_5,6,7________
	6b	Explanation for choice of comparators	_5,6,7_________
Objectives	7	Specific objectives or hypotheses	__8___________
Trial design	8	Description of trial design including type of trial (eg, parallel group, crossover, factorial, single group), allocation ratio, and framework (eg, superiority, equivalence, noninferiority, exploratory)	__9,10_________
**Methods: Participants, interventions, and outcomes**			
Study setting	9	Description of study settings (eg, community clinic, academic hospital) and list of countries where data will be collected. Reference to where list of study sites can be obtained	___9,10________ _
Eligibility criteria	10	Inclusion and exclusion criteria for participants. If applicable, eligibility criteria for study centres and individuals who will perform the interventions (eg, surgeons, psychotherapists)	___10_________
Interventions	11a	Interventions for each group with sufficient detail to allow replication, including how and when they will be administered	_11,12,13,14,15 __
	11b	Criteria for discontinuing or modifying allocated interventions for a given trial participant (eg, drug dose change in response to harms, participant request, or improving/worsening disease)	_____10________
	11c	Strategies to improve adherence to intervention protocols, and any procedures for monitoring adherence (eg, drug tablet return, laboratory tests)	_____15________

	11d	Relevant concomitant care and interventions that are permitted or prohibited during the trial	____11,15______
Outcomes	12	Primary, secondary, and other outcomes, including the specific measurement variable (eg, systolic blood pressure), analysis metric (eg, change from baseline, final value, time to event), method of aggregation (eg, median, proportion), and time point for each outcome. Explanation of the clinical relevance of chosen efficacy and harm outcomes is strongly recommended	___16________
Participant timeline	13	Time schedule of enrolment, interventions (including any run-ins and washouts), assessments, and visits for participants. A schematic diagram is highly recommended (see Figure)	__12,13, Figure 1
Sample size	14	Estimated number of participants needed to achieve study objectives and how it was determined, including clinical and statistical assumptions supporting any sample size calculations	___20,21_____
Recruitment	15	Strategies for achieving adequate participant enrolment to reach target sample size	_9,10,20,21_____

Methods: Assignment of interventions (for controlled trials)

Allocation:

Sequence generation	16a	Method of generating the allocation sequence (eg, computer-generated random numbers), and list of any factors for stratification. To reduce predictability of a random sequence, details of any planned restriction (eg, blocking) should be provided in a separate document that is unavailable to those who enrol participants or assign interventions	___12________
Allocation concealment mechanism	16b	Mechanism of implementing the allocation sequence (eg, central telephone; sequentially numbered, opaque, sealed envelopes), describing any steps to conceal the sequence until interventions are assigned	_____12_______
Implementation	16c	Who will generate the allocation sequence, who will enrol participants, and who will assign participants to interventions	___12________
Blinding (masking)	17a	Who will be blinded after assignment to interventions (eg, trial participants, care providers, outcome assessors, data analysts), and how	___12________

	17b	If blinded, circumstances under which unblinding is permissible, and procedure for revealing a participant's allocated intervention during the trial	____12________

Methods: Data collection, management, and analysis

Data collection methods	18a	Plans for assessment and collection of outcome, baseline, and other trial data, including any related processes to promote data quality (eg, duplicate measurements, training of assessors) and a description of study instruments (eg, questionnaires, laboratory tests) along with their reliability and validity, if known. Reference to where data collection forms can be found, if not in the protocol	____16_______
	18b	Plans to promote participant retention and complete follow-up, including list of any outcome data to be collected for participants who discontinue or deviate from intervention protocols	____10_______
Data management	19	Plans for data entry, coding, security, and storage, including any related processes to promote data quality (eg, double data entry; range checks for data values). Reference to where details of data management procedures can be found, if not in the protocol	____13_______
Statistical methods	20a	Statistical methods for analysing primary and secondary outcomes. Reference to where other details of the statistical analysis plan can be found, if not in the protocol	_____21,22____
	20b	Methods for any additional analyses (eg, subgroup and adjusted analyses)	____21,22_____
	20c	Definition of analysis population relating to protocol non-adherence (eg, as randomised analysis), and any statistical methods to handle missing data (eg, multiple imputation)	____21,22____

Methods: Monitoring

Data monitoring	21a	Composition of data monitoring committee (DMC); summary of its role and reporting structure; statement of whether it is independent from the sponsor and competing interests; and reference to where further details about its charter can be found, if not in the protocol. Alternatively, an explanation of why a DMC is not needed	_____23______
	21b	Description of any interim analyses and stopping guidelines, including who will have access to these interim results and make the final decision to terminate the trial	_____N/A______

Harms	22	Plans for collecting, assessing, reporting, and managing solicited and spontaneously reported adverse events and other unintended effects of trial interventions or trial conduct	______23______
Auditing	23	Frequency and procedures for auditing trial conduct, if any, and whether the process will be independent from investigators and the sponsor	_____23,25______
**Ethics and dissemination**			
Research ethics approval	24	Plans for seeking research ethics committee/institutional review board (REC/IRB) approval	_____25______
Protocol amendments	25	Plans for communicating important protocol modifications (eg, changes to eligibility criteria, outcomes, analyses) to relevant parties (eg, investigators, REC/IRBs, trial participants, trial registries, journals, regulators)	_____25_______
Consent or assent	26a	Who will obtain informed consent or assent from potential trial participants or authorised surrogates, and how (see Item 32)	_____10_______
	26b	Additional consent provisions for collection and use of participant data and biological specimens in ancillary studies, if applicable	_____N/A_______
Confidentiality	27	How personal information about potential and enrolled participants will be collected, shared, and maintained in order to protect confidentiality before, during, and after the trial	_____25_______
Declaration of interests	28	Financial and other competing interests for principal investigators for the overall trial and each study site	_____26_______
Access to data	29	Statement of who will have access to the final trial dataset, and disclosure of contractual agreements that limit such access for investigators	_____25_______
Ancillary and post-trial care	30	Provisions, if any, for ancillary and post-trial care, and for compensation to those who suffer harm from trial participation	_____N/A_______
Dissemination policy	31a	Plans for investigators and sponsor to communicate trial results to participants, healthcare professionals, the public, and other relevant groups (eg, via publication, reporting in results databases, or other data sharing arrangements), including any publication restrictions	______25_______
	31b	Authorship eligibility guidelines and any intended use of professional writers	_____25_______

For peer review only

	31c	Plans, if any, for granting public access to the full protocol, participant-level dataset, and statistical code	____25_______
**Appendices**			
Informed consent materials	32	Model consent form and other related documentation given to participants and authorised surrogates	____S1,S2____ __
Biological specimens	33	Plans for collection, laboratory evaluation, and storage of biological specimens for genetic or molecular analysis in the current trial and for future use in ancillary studies, if applicable	____S1______

*It is strongly recommended that this checklist be read in conjunction with the SPIRIT 2013 Explanation & Elaboration for important clarification on the items. Amendments to the protocol should be tracked and dated. The SPIRIT checklist is copyrighted by the SPIRIT Group under the Creative Commons "Attribution-NonCommercial-NoDerivs 3.0 Unported" license.

For peer review only

BMJ Open

Effects of an iso-energetic low glycaemic index (GI) diet on liver fat accumulation and gut microbiota composition in patients with non- alcoholic fatty liver disease (NAFLD): A study protocol of an efficacy mechanism evaluation

Journal:	*BMJ Open*
Manuscript ID	bmjopen-2020-045802.R2
Article Type:	Protocol
Date Submitted by the Author:	17-Aug-2021
Complete List of Authors:	Al-Awadi, Amina; University of Nottingham, Nottingham Digestive Diseases Centre<br>Grove, Jane; University of Nottingham, Nottingham Digestive Diseases Centre<br>Taylor, Moira ; University of Nottingham School of Life Sciences,<br>Valdes, A; University of Nottingham,<br>Vijay, Amrita; University of Nottingham, Nottingham Digestive Diseases Centre<br>Bawden, Stephen; University of Nottingham, Physics and Astronomy<br>Gowland, Penny; University of Nottingham, Department of Physics and Astronomy<br>Aithal, Guruprasad; Nottingham Digestive Diseases Biomedical Research Centre, Nottingham Digestive Diseases Centre
**Primary Subject Heading**:	Nutrition and metabolism
Secondary Subject Heading:	Nutrition and metabolism, Diabetes and endocrinology, Gastroenterology and hepatology
Keywords:	Hepatology < INTERNAL MEDICINE, NUTRITION & DIETETICS, Hepatobiliary disease < GASTROENTEROLOGY

SCHOLARONE™ Manuscripts

I, the Submitting Author has the right to grant and does grant on behalf of all authors of the Work (as defined in the below author licence), an exclusive licence and/or a non-exclusive licence for contributions from authors who are: i) UK Crown employees; ii) where BMJ has agreed a CC-BY licence shall apply, and/or iii) in accordance with the terms applicable for US Federal Government officers or employees acting as part of their official duties; on a worldwide, perpetual, irrevocable, royalty-free basis to BMJ Publishing Group Ltd ("BMJ") its licensees and where the relevant Journal is co-owned by BMJ to the co-owners of the Journal, to publish the Work in this journal and any other BMJ products and to exploit all rights, as set out in our licence.

The Submitting Author accepts and understands that any supply made under these terms is made by BMJ to the Submitting Author unless you are acting as an employee on behalf of your employer or a postgraduate student of an affiliated institution which is paying any applicable article publishing charge ("APC") for Open Access articles. Where the Submitting Author wishes to make the Work available on an Open Access basis (and intends to pay the relevant APC), the terms of reuse of such Open Access shall be governed by a Creative Commons licence – details of these licences and which Creative Commons licence will apply to this Work are set out in our licence referred to above.

Other than as permitted in any relevant BMJ Author's Self Archiving Policies, I confirm this Work has not been accepted for publication elsewhere, is not being considered for publication elsewhere and does not duplicate material already published. I confirm all authors consent to publication of this Work and authorise the granting of this licence.

For peer review only

Title: Effects of an iso-energetic low glycaemic index (GI) diet on liver fat accumulation and gut microbiota composition in patients with non- alcoholic fatty liver disease (NAFLD): A study protocol of an efficacy mechanism evaluation

Authors: Amina Al-Awadi, Jane Grove, Moira Taylor, Ana Valdes, Amrita Vijay, Stephen Bawden, Penny Gowland, Guruprasad Aithal.

Corresponding Author: Guruprasad P. Aithal**,** Nottingham Digestive Diseases Centre, School of Medicine, University of Nottingham, Nottingham UK, NG7 2UH.
Email address: mszag3@exmail.nottingham.ac.uk
Tel: +44 (0)115 823 1031

AUTHORS AFFILIATIONS

Amina A. Al-Awadi, Nottingham Digestive Diseases Centre, University of Nottingham. NIHR Nottingham Biomedical Research Centre, Nottingham University Hospitals Trust and the University of Nottingham. Nottingham, United Kingdom.

Jane I. Grove, Nottingham Digestive Diseases Centre, University of Nottingham. NIHR Nottingham Biomedical Research Centre, Nottingham University Hospitals Trust and the University of Nottingham. Nottingham, United Kingdom.

Moira A. Taylor, Department of Nutrition and Dietetics, School of Life Sciences, University of Nottingham, Nottingham, United Kingdom.

Ana M. Valdes, NIHR Nottingham Biomedical Research Centre, Nottingham University Hospitals Trust and the University of Nottingham. Division of Rheumatology Orthopaedics and Dermatology, School of Life Sciences, University of Nottingham. Nottingham, United Kingdom

Amrita Vijay, Nottingham Digestive Diseases Centre, University of Nottingham. Division of Rheumatology Orthopaedics and Dermatology, School of Life Sciences, University of Nottingham. Nottingham, United Kingdom.

Stephen J. Bawden, Sir Peter Mansfield Imaging Centre, School of Physics and Astronomy, University of Nottingham, Nottingham, United Kingdom.

Penny A. Gowland, Sir Peter Mansfield Imaging Centre, School of Physics and Astronomy, University of Nottingham, Nottingham, United Kingdom.

Guruprasad P. Aithal, Nottingham Digestive Diseases Centre, University of Nottingham. NIHR Nottingham Biomedical Research Centre, Nottingham University Hospitals Trust and the University of Nottingham. Nottingham, United Kingdom.

Key words: Nutrition and Dietetics, Hepatology, Hepatobiliary disease, Fatty liver, glycaemic index.
Word count: 5234

For peer review only

ABSTRACT:

Introduction A low glycaemic index (LGI) diet is a proposed lifestyle intervention in non-alcoholic fatty liver diseases (NAFLD) which is designed to reduce circulating blood glucose levels, hepatic glucose influx, insulin resistance, and *de novo* lipogenesis. A significant reduction in liver fat content through following a one-week LGI diet has been reported in healthy volunteers. Changes in dietary fat and carbohydrates have also been shown to alter gut microbiota composition and lead to hepatic steatosis through the gut-liver axis. There are no available trials examining the effects of a LGI diet on liver fat accumulation in patients with NAFLD; nor has the impact of consuming a LGI diet on gut microbiota composition been studied in this population. The aim of this trial is to investigate the effects of LGI diet consumption on liver fat content and its effects on gut microbiota composition in participants with NAFLD compared with a high glycaemic index (HGI) control diet.

Methods and Analysis A 2x2 crossover randomized mechanistic dietary trial will allocate 16 participants with NAFLD to a 2-week either HGI or LGI diet followed by a 4-week wash-out period and then the LGI or HGI diet, alternative to that followed in the first 2-weeks. Baseline and post-intervention (4 visits) outcome measures will be collected to assess liver fat content (using MRI/S and CAP-Fibroscan™), gut microbiota composition (using 16S RNA analysis) and blood biomarkers including glycaemic, insulinemic, liver, lipid and hematologic profiles, gut hormones levels, and short chain fatty acids (SCFAs).

Ethics and Dissemination Study protocol has been approved by the ethics committees of The University of Nottingham and East Midlands Nottingham-2 Research Ethics

For peer review

Committee (REC reference 19/EM/0291). Data from this trial will be used as part of a Philosophy Doctorate thesis. Publications will be in peer reviewed journals.

Trial registration number (NCT04415632, protocol v1.1, 28/10/2019)

For peer review only

ARTICLE SUMMARY:

Strengths

- A carefully controlled human LGI diet intervention to reduce liver fat in NAFLD using cross-over study deign.
- The first NAFLD patient study that explores the mechanistic effects of dietary glycaemic index on liver fat accumulation using advanced MR protocols.
- Uses the controlled attenuation parameter (CAP™) as a biomarker to evaluate efficacy of diet intervention.

Limitations:

- Findings from small sample size may not be generalizable to all patients with NAFLD so will require replication in larger long-term randomised controlled trial.
- The 2 week time frame of the intervention, especially in light of isoenergetic diet, may not be sufficient to observe significant changes in liver fat.

For peer review only

INTRODUCTION

Non-alcoholic fatty liver disease (NAFLD) is a growing global health concern increasing in parallel with the worsening epidemics of obesity and diabetes[1 2]. Recent reviews indicated that one billion people worldwide have NAFLD (estimated prevalence 25.4%)[3]. NAFLD is characterized by the fat accumulation in hepatocytes exceeding 5% of liver weight and encompasses a spectrum of disease severities. Common clinical risk factors that are highly associated with NAFLD include obesity, insulin resistance, type 2 diabetes, and metabolic syndrome[3 4]. NAFLD is recognized as the hepatic manifestation of metabolic syndrome[5-7].

Western dietary habits occurring concurrently with a sedentary lifestyle and genetic factors are associated with insulin resistance, adipocyte proliferation and gut microbiota alteration which may be implicated in NAFLD[8]. Dietary habits, hormones secreted from the adipose tissue, gut microbiota composition and genetic factors are all implicated in the pathogenesis.

To date, there are no drugs with regulatory approval for treating NAFLD, and lifestyle changes including modification of dietary patterns together with increased physical activity is the initial step to treat this disease[9]. Dietary approaches beneficial in NAFLD management include reductions in saturated and trans fatty acids, total carbohydrates, and animal-based protein intake and increases in the intake of polyunsaturated fatty acids (PUFAs), monounsaturated fatty acids (MUFAs), plant- based proteins, and antioxidants[4]. Furthermore, evidence from a systematic review and meta-analysis of randomized control trials (RCT) on patients with NAFLD has shown that lifestyle-induced weight loss of ≥ 7%

For peer review only

improved histological disease activity, as well as cardiometabolic risk profile, in less than 50% of the study population (n=373 patients)[10]. A recent systematic review has concluded that Despite the abundance of interventional studies on dietary management in NAFLD, there is still a lack of evidence-based specific recommendations, other than that weight loss may benefit some; and few studies have measured liver-related outcomes[11]. Moreover, the dietary effects on liver-related outcomes that are mediated by gut microbiome have not been studied in these patients[12].

More recently, the glycaemic index (GI) and glycaemic load (GL), of diet; has been proposed as an important predictor of NAFLD progression[4 13]. The GI of a food ranks the glycaemic response under conditions in which the quantity of carbohydrate is controlled[14 15], whereas the GL additionally considers portion size[16].

NAFLD patients have been found to consume a diet high in HGI foods[4] which are correlated with insulin resistance, metabolic dysfunction[4], and liver dysfunction[17]. In addition, HGI dietary habits have been significantly associated with high-grade liver steatosis in patients with insulin resistance[18] while limiting HGI foods is beneficial in NAFLD[19 20]. Further, previous pilot study showed that only one-week of consuming HGI diet caused significant increase in hepatic stores of fat when compared with isocaloric LGI diet consumption[21]. A recent review concluded that both low GI and GL diets resulted in significant reductions in hepatic fat mass in a cohort of 269 participants[16]. However, these effects were not attributed to the LGI diet alone and were confounded with other lifestyle interventions[16].

For peer review only

Evidence from animal and human studies also showed that diet is the most important modifying factor in the development of human gut microbiome [22-24]. Dietary alterations can rapidly change the human gut microbiota composition in relatively short time periods[25-27] and in as little as 24 hours[28-30]. Certain dietary factors which modify gut microbiota have been associated with a number of diseases including obesity[31] metabolic syndrome[22 32 33], type 2 diabetes[34], immunological dysfunctions[35]. Such dysbiosis in diet-induced NAFLD has been shown to stimulate hepatic fat disposition and promote NAFLD progression to non-alcoholic steatohepatitis (NASH)[36 37]. This is suggested to be mediated by modulation of gut permeability, dietary choline metabolism, bile acid metabolism, endogenous ethanol production and immune balance [36 37]. Gut microbiota modulated by diet can increase the energy extraction from the breakdown of non-absorbable polysaccharides with the production of monosaccharides and short chain fatty acids (SCFAs) which are substrates for hepatic *de novo* lipogenesis and carbohydrate biosynthesis[38]. Certain SCFAs have also been implicated in improving glucose and lipid metabolism beneficial to the host[39]. Therefore, there is a potential to modulate gut microbiota through dietary changes as a means of NAFLD prevention or alleviation[11 40].

We describe here our study protocol designed to assess the effects of a specific defined GI dietary manipulation[21] on liver fat content and its effects on gut microbiota composition in participants with NAFLD compared with a higher GI diet as a control diet. The diets will be adjusted to each participant to meet their energy requirements and the macronutrient composition of the diets will be carefully controlled ensuring that differences in GL are due to differences in GI.

For peer review only

OBJECTIVES

Primary Objective

1. To investigate the effects of defined LGI versus HGI diet on liver fat accumulation in participants with NAFLD following a two-week intervention period whilst controlling for the energy consumption and amount of carbohydrate intake.

Secondary Objectives

1. To investigate the effects of LGI versus HGI diet on gut microbiota composition and its correlations with liver outcomes in participants with NAFLD following a two-week intervention period.
2. To investigate the changes in blood metabolic biomarkers (glycaemic, insulinemic, liver, lipid, hematologic profiles, gut hormones, and plasma SCFAs) between LGI and HGI diet groups following a two-week intervention in participants with NAFLD.
3. To evaluate CAP™ as a tool to test the efficacy of a dietary intervention by comparing its performance characteristics with that of Magnetic Resonance Spectroscopy (MRS) as a gold standard.
4. To validate the European Prospective Investigation of Cancer food frequency questionnaire (EPIC-FFQ) in Nottingham NAFLD patients against food diary.

For peer review only

METHODS AND ANALYSIS

Trial Design

This is a randomized 2 x 2 cross-over mechanistic trial including two arms, each of two weeks duration comparing a LGI diet and a control HGI diet based on that defined by Bawden et al. (2017)[21] and Morgan et al. (2012)[41]. The study design is illustrated in Figure 1.

Public and patient involvement

We collaborated with the NIHR Nottingham BRC patient advisory group, and we met 7 patients who were interested in study participation. They expressed strong support for the research objectives and the recent meeting the group considered the one-week crossover intervention feasible and acceptable. Patient feedback informed the participant recruitment through information content and delivery methods. They also assisted in the study design to enhance participant compliance and commitment to the diet.

Participants and Recruitment

We aim to recruit 16 participants with NAFLD to complete both phases of 2x2 cross-over trial. The initial screening criteria for inclusion is a CAP™ score of >288dB/m; this value correlates with Magnetic Resonance Imaging (MRI)- Proton Density Fat Fraction (PDFF) estimation of > 5% fat of liver weight which indicates that they have a fatty liver[42].

NAFLD patients will be recruited either from the existing database of “The Scarred Liver Project” in the UK who have given consent to be contacted to participate in future research

For peer review only

(approved by East Midlands-Leicester Research ethics committee ref: B/EM/0123) or from liver clinics at the Nottingham University Hospitals NHS Trust who had a Fibroscan with CAP or participants from public (advert in local and social media). The eligibility criteria are summarized in Table 1. All participants will provide a written informed consent by trained research team before they enter the trial. Potential participants will be free to withdraw at any time. In the event of their withdrawal, it will be explained that their data collected so far cannot be erased and we will seek consent to use the data in the final analyses where appropriate. In the event of a participant withdrawing or being unable to continue the intervention periods after randomisation, a replacement will be recruited who will be assigned to the same diet group to ensure a 1:1 ratio for the 2 diet groups. We will continue recruiting until 16 participants have fully completed the study.

**Table 1: Eligibility Criteria**
Inclusion criteria<br>• Adult males and females aged from 18 to 65 years (balanced number).<br>• Detected NAFLD by CAP-FibroScan™ >288dB/m or by MRI-PDFF) > 5% fat of liver weight.<br>• Body mass index ≥ 25 kg/m^2<br>• Abdominal obesity (Waist circumference > 102 cm for males and > 88 cm for females)<br>• Have current moderate to high GI diet intake of ≥ 60 (Assessed from a completed 7-day food diary).<br>• Able to give informed consent.<br>• Able to undergo MRI/S and CAP-FibroScan™.
Exclusion criteria<br>• Current smokers and excessive alcohol drinkers (> 14 units/week).<br>• Perimenopausal (irregular periods) women.<br>• Participants with other liver abnormalities.<br>• Participants with history of gastrointestinal surgeries, depression, eating disorders or difficulties.<br>• Participants using pharmacologic agents for obesity or NAFLD.<br>• Participants with type 1 diabetes.<br>• Participants with type 2 diabetes on second line medications (eg GLP-1 analogues).<br>• Participation in any other trial in the last 3 months.<br>• Participants on any special diets (e.g. vegetarians).<br>• Intolerance to foods included in the diet plan.

For peer review only

Screening process:

Participants will be invited to attend an initial screening visit (Figure 1). At this visit, participants will be consented and asked to complete a pre-screening questionnaire (including taking height, weight, and blood pressure measurements) to establish whether they meet the study criteria. Participants will be asked to fill out a 7-day food records and post it after one week of their initial screening visit. This is to avoid giving patients with NAFLD, who might be at risk of developing diabetes, a much higher HGI diet than they habitually consume. Using 7-day food records, we will characterise the baseline habitual GI diet intake of all potential volunteers at screening period. Participants also will be required to complete a food frequency questionnaire (FFQ) for validation against the 7-day food records. We will use EPIC-FFQ that was validated for use in Norfolk cohort[43]. After completion, participant's eligibility will be reassessed based on analysis of diet information using Nutritics software (Nutritics Ltd, Dublin, Ireland, 2018). Those with a habitual LGI diet intake (GI < 60) will be excluded from the study. Only patients who have a moderate to HGI diet intake (GI ≥ 60) will be invited to attend the next screening preparation visit.

Then at next screening visit (visit 2), the participants will be asked to fill out International Physical Activity Questionnaire (IPAQ)[44]. A trained dietitian will instruct participants on how to measure study food provided using measuring jug and spoons and how to fill in study diet compliance sheets. They will be instructed on stool collection and pre-

For peer review only

intervention visits requirements. Participants will be asked to avoid eating probiotics and/or prebiotics supplements or drinks for one week before the intervention period (standardization week). All instructions will be provided to each participant in a form of Home Guide Booklet.

Each eligible participant will be assigned randomly to undergo either HGI diet (HGI group) or LGI group first and then crossover to either HGI or LGI diets.

Randomisation and Blinding

Enrolled participants who meet the eligibility criteria will be randomized to one of two groups which will dictate the order in which they receive the LGI or HGI diet. They will then receive the second diet depending upon which diet they have first received. The random allocation sequence (AB or BA) will be generated by one of the trial co-ordinators; who will have no contact with the participants, using block randomization by impartial biostatistics. The online randomisation package (http://randomization.com) will be used to allocate the sequences of the diet groups to the participants in equal numbers. Once the random number is generated, both trial investigators and participants are aware of the diet sequence (i.e., whether the participant is going to start with LGI diet; A or HGI diet; B). The diets then will be delivered to the participant according to the sequence. The trial outcome measures will be determined by blinded researcher using codes on samples that will be sent for 16S ribosomal ribonucleic acid (rRNA), blood marker and MRI/CAP-FibroScan™ analyses. The blinded allocation will be provided to the study statistician at the end of the study and once the analysis has been completed, the study statistician will be provided with the un-blinded allocation.

For peer review only

Study protocol:

Following the screening phase and standardization week, each participant will experience two, two-week intervention phases, with a four-week washout period between interventions. Before and after each intervention period they will attend the Imaging Centre (Sir Peter Mansfield Imaging Centre) for MRI scan measurements and then will attend the Biomedical Research Centre for Fibroscan, and blood and stool samples collection. During each intervention phase each participant will be required

to consume all the food that has been provided, fill in dietary compliance sheets and complete visual analogue scales (VAS) relating to subjective appetite ratings.

Design of the dietary interventions.

Energy expenditure will be estimated for each participant using the_individual's basal metabolic rate (BMR) calculated from Henry-modified Schofield formula[45] and multiplied by an activity factor based on the IPAQ score physical activity levels (PALs). A menu, consisting of meals for 3 days personalised for energy requirement, will be devised (these meals are repeated over 2 weeks) by adjusting a template according to their estimated energy requirement in order to minimise weight change during the intervention phase. The diet consists of normal food closely resembling that described by Bawden *et al*, (2017) study[21]. A diet template of 2000 kcal/day with energy proportions distributed as 55% from carbohydrates, 15% from protein and 30% from fat[46] are designed and food quantities will be modified to supply the energy requirements for each participant. Three-day cycle menu plans are designed for each diet (LGI/ HGI). Table 2 shows example template menus for both HGI (a) and LGI (b) diets.

For peer review only

Table 2 Illustration of HGI (a) and LGI (b) diet templates for use in the study.

HGI Diet Sample (a)	Weight (g)	Energy (Kcal)	Carbohydrates (g)	Protein (g)	Fat (g)	Fibre (g)	GI	GL
Breakfast								
Sultana Bran Cereal	90	268	58	6.6	1.3	10.1	90	52.2
Skimmed Milk	180	63	8.3	6.3	0.54	0	48	4
Roasted Peanuts	15	90	2.3	3.7	7.4	1.3	14	0.32
Glucose	10	40	10	0	0	0	100	10
Water	170	0	0	0	0	0	0	0
Meal Total		461	78	16.6	9.2	11.4	85.3	66.5
Lunch								
Margarine	25	102	0.13	0.05	11.3	0	0	0
Fruit loaf	144	395	76	11.4	5.2	4.6	90	68.4
Roasted turkey slice	42	47	0.67	10	0.5	0.12	0	0
Cheddar cheese slice	25	104	0.03	6.4	8.7	0	0	0
Strawberry yogurt	125	88	16.3	5.6	0	0.38	85	13.855
Meal Total		735	93	33.4	25.7	5.1	88.44	82.255
Dinner								
Cottage Pie (ready meal)	400	441	44	18	21.6	4.8	66	29.04
Low fat Strawberry yogurt	125	88	16.3	5.6	0	0.38	85	13.855
Water	170	0	0	0	0	0	0	0
Glucose	10	40	10	0	0	0	100	10
Mars bar	58	237	37	2.4	8.9	0.87	65	24.05
Meal Total		806	107	26	30.5	6	72.69	76.954
Menu Total		2002 Kcal	278g (55.5%)	76g (15.2%)	65g (29.3%)	22g	82.1	225.706

For peer review only

LGI Diet Sample (b)

	Weight (g)	Energy (Kcal)	Carbohydrates (g)	Protein (g)	Fat (g)	Fibre (g)	GI	GL
Breakfast								
All Bran Cereal	90	239	42	11.2	3.1	22.1	43	18.06
Semi-Skimmed Milk	160	76	7.2	5.6	2.7	0	25	1.8
Orange juice	250	96	22	1.5	0.25	0	53	11.66
Meal Total		410	71	18.2	6	22.1	44.39	31.52
Lunch								
Pumpernickel bread	180	408	74	15.7	5.6	11.7	41	30.34
Black cherry yogurt	150	209	24.2	4.8	10.4	0.75	17	4.114
Soft cheese	51	129	1.5	2.7	12.4	0	34	0.51
Meal Total		745	99	23.2	28.4	12.5	35.32	34.96
Dinner								
Lasagne (ready meal)	290	413	42	21.5	17.7	4.9	36	15.12
Apple	174	92	20	1	0.87	2.1	38	7.6
Semi-skimmed milk	150	71	6.8	5.3	2.6	0	25	1.7
Fruit &nuts mix	60	267	24.6	8.4	15	3.9	15	3.69
Meal Total		843	93	36.2	36.1	10.9	30.22	28.11
Menu Total		1999 Kcal	264g (52.7%)	78g (15.5%)	71g (31.8%)	46g	36.64	94.594

The GI values of commercial food items are generated from that reported by Henry *et al*. (2005)[47], as well as Nutritics software databases (Nutritics Ltd, Dublin, Ireland, 2018). The dietary GI of the menu is calculated from GI proportional contribution to total carbohydrate using the equation: $GI = \sum(C_{food}/C_{total}) \times GI_{food}$; where C_{food} is the amount of carbohydrate contained in each digested food (g), C_{total} is the total carbohydrate in the meal and GI_{food} is the reported GI of the food[21]. The GL of the diet is calculated based on the dietary GI and the amount of carbohydrate provided (the same in both diets) using the following equation: $GL = (GI_{food} \times C_{food}) / 100$; where GI_{food} is the GI of each digested food and C_{food} is the amount of carbohydrate in the food[21]. Food composition analysis and evaluation were performed using Nutritics software (Nutritics Ltd, Dublin, Ireland, 2018).

For peer review only

Participants will be provided with the food at the start of each two-week diet period and will be clearly instructed on safe food storage and reheating. They will be required to complete dietary compliance sheet on a daily basis. Instructions on dietary compliance will be given at each study period and the dietary compliance records will be reviewed regularly during the intervention to assess their compliance. For the first one week prior to the intervention (one-week standardization), and during the four-week washout period, participants will be asked to avoid eating probiotics, yogurt and cultured milk drink; if any, to standardize their gut microbiota and avoid any carry-over effects as shown in previous studies[48 49]. Meal-timing will be standardised among all participants as much as possible during the intervention as well as during the standardisation period.

Data Collection and Analyses of outcome measures:

The timing and frequency of study measurements are depicted in Figure 1. Participants will be asked to come fasted (a minimum of 8 hours) for each laboratory visit (pre and post each intervention period) and the following measurements will be made:

1. *Anthropometric measurements:* Participant height (cm), weight (kg), waist circumference (cm), body mass index (kg/m^2) and body composition with bladder voided using Bio-electrical Impedance analysis (BIA - ACC appliance model BIA00A; BioTekna, Marcon VE, Italy).

2. *Liver fat assessment:* Hepatic fat fraction and comparison lipid composition will be measured using a Philips 3T Acheiva MRI scanner with 32 channel receive XL torso coil

For peer review only

and advanced lipid composition analysis on a Philips 7T Acheiva scanner with 8 channel multi transmit body coil. MRS will be acquired from a 20x20x20mm voxel within the lower right lobe of the liver using Stimulated Echo Acquisition Mode (STEAM) localization to assess hepatic fat fraction and composition. Spectra will be acquired over four echo times (TE 20, 30, 40, 80) to correct for T_2 relaxation over 5 breath-holds. Water will be used as a reference peak, and water suppression used to analyse signal from lipids. Spectra will be post processed (phase corrected, line-broadened, frequency aligned and averaged) as described previously[50], and then fitted according to known lipid signal positions[51]. T_2 corrected PDFF will be calculated as PDFF = FAT / (FAT+WATER) and converted to true mass fat fraction using known tissue values[52]. Lipid composition will be estimated using individual peak amplitudes[53].Subcutaneous and visceral fat volumes will be determined using gradient echo Magnetic Resonance (MR) imaging[54].

Liver fat levels will also be assessed with the CAP™ scored using Fibroscan™, which has previously been evaluated against MRS[55] as well as liver biopsy[56] in cross sectional cohorts. Although MRI-PDFF more accurately classifies steatosis[57], portability, accessibility and costs favour CAP™ associated with transient elastography. However, CAP™ has not been evaluated as a marker of efficacy of intervention. For the purpose of validation, participants will undergo a CAP-Fibroscan™ at each intervention visit to quantify their liver fat levels.

3. *Hepatic ATP flux:* Rate of mitochondrial ATP turnover in the liver will be assessed using a Philips 3T MRI scanner with a Philips Phosphorous (31P) single loop surface coil. 31P MRS will be acquired from the liver using a 60mm thick slice to avoid contamination from abdominal muscle. A progressive saturation scan protocol (5 spectra acquired with varying length pre-saturation pulses) will be used to determine apparent T1[58]. Two 31P MR spectra will then be acquired with full saturation of gamma ATP (-2.5ppm) and mirrored downfield of the inorganic phosphate peak (~14ppm) respectively. An in-house fitting program will be used for post-processing spectra and measuring the saturation transfer to the inorganic phosphate peak and used to determine hepatic ATP flux rate constant. ATP concentration will also be determined from spectra and used to calculate final ATP flux rate values[59].

4. *Metabolic Biomarkers:* Blood samples (not more than 80 mL at each visit) will be taken from fasted participants (12-hours fast) for analyses of metabolic analytes such as: glycaemic, insulinemic, liver, lipid, hematologic profiles, gut hormones (ghrelin, leptin, glucagon-like peptide-1 (GLP-1), glucagon) and plasma SCFAs. Although GLP-1 levels increase in response to meal ingestion, levels can be directly affected by presence of SCFAs, produced from fermentation of certain carbohydrates which may have been recently consumed, confounding the results. Instead, only fasting levels will be measured as short-term diets containing different carbohydrates types and different GI content have been found to affect fasting GLP-1 levels[60]. In addition, circulating plasma SCFAs levels will be measured as it has been shown to be strongly related to metabolic health[61]. Measurement of plasma SCFAs levels also overcomes the

For peer review only

challenges associated with accurate quantification of volatile SCFAs molecules in fecal samples[62].

All biochemical parameters concentrations will be assessed by a central laboratory that uses standard methods. Standard operating procedures (SOPs) will be followed for all laboratory assays to ensure the high quality and reliability of our generated data. Routine blood tests will be analysed by the accredited laboratories. Samples will be stored in -80˚C freezers until the study ends, under yearly service contract and monitoring systems. Analyses will be done according to assay manufacturer advice and completed by technicians and research staff who are formally trained or outsourced to collaborators or external suppliers where necessary. The insulin resistance will be estimated using homeostatic model assessment of insulin resistance (HOMA-IR). HOMA-IR is calculated according to the equation [HOMA-IR = (FPI× FPG)/22.5]; where FPI is fasting plasma insulin, and FPG is fasting plasma glucose[42].

6. *Gut Microbiota Composition*: Each participant will be asked to collect fresh stool sample in a standard laboratory container and bring it to the research centre with an ice pack for storage at -80°C. Stool samples will be processed using a standard commercial kit for Deoxyribonucleic acid (DNA)extraction[63]. Then, the gut microbiota composition will be assessed using 16S sequencing of rRNA. DNA will be diluted to 20ng/µl for 16S rRNA amplification and sequencing. Water negative controls will be included from extraction, through polymerase chain reaction (PCR) to sequencing and select samples were sequenced in duplicate for quality control. The V4 region of the 16S rRNA gene will be amplified using universal primers 355F (CCAGACTCCTACGGGAGGCAGC) and 806R (GGACTACHVGGGTWTCTAAT). Amplified DNA will be sequenced on the MiSeq platform (Illumina). Read filtering and

For peer review only

clustering will carried out using the MYcrobiota pipeline56. Briefly, chimeric sequences will be filtered using the VSEARCH algorithm within Mothur, and reads will be clustered into operational taxonomic units (OTUs) using closed-reference clustering against the SILVA database v132 based on a 97% similarity. Diversity metrics (Shannon index, observed OTUs and Unweighted UniFrac) will be calculated by rarefying the OTU table down to 7000 sequences per sample 50 times and taking the average. These analyses will be carried out in QIIME 2 (v2018.11)[64 65].

7. *Subjective Sensation Assessments:* VAS scores will be used to assess the participant's subjective appetite (hunger and fullness) during the intervention. Participants will be provided with a booklet to record their subjective appetite before and after each meal during the intervention at selected days.

Sample Size Calculation and Justification:

The sample size is calculated using nQuery based on previous completed study by Bawden *et al*, (2017)[21]. Based on their pilot data, baseline liver fat fractions of both diet groups were 2.4 ± 1-1.2%, and after 1 week were HGI (High GI) = 4.7 ± 2 % and LGI = 1.6 ± 0.7%. Using a two-group t-test (Crossover ANOVA) between the post- diet values with a 0.05 two-sided significance level, this produces a sample size of n=8 in each diet sequence (a total sample size of 16). This sample size has an 80% power to detect a difference in means of 3.1 (the difference between a Treatment 1, μ_1, of 4.7 and a Treatment 2, μ_2, of 1.6) with the assumption that the Crossover ANOVA sqrt (MSE) is

For peer review only

2.828 (the Standard deviation of differences, σ, is 4). Assuming that 40% of people would drop out from cross-over study design, it is expected that at least 24 participants would need to be recruited.

The effect from the pilot study by Bawden, *et al*, (2017) pilot study (n=7) was an average 1.6% ± 0.7% decrease in liver fat fraction after following a one-week LGI diet ($p < 0.05$). Given that baseline liver fat fraction was 2.4%, in order to see such change with 80% power ($p< 0.05$) we have doubled the participant numbers to n=16 using nQuery software to detect statistically significant differences[21]. Our group has recently completed a study (ClinicalTrials.gov Identifier: NCT03844165) in which 84 participants with NAFLD followed either a LGI food enriched diet or their normal diet. Patients who changed their diet were found to have a significant decrease in liver fat after 16 weeks (from 20.4% to 18.8%) and had a statistically significant decrease in fasting glucose levels after the diet compared to the control group patients following their normal diet. This provides strong support to our hypothesis and demonstrates that a simple dietary change can have measurable impact on NAFLD symptoms in a short period. In another previous randomized crossover trial, metabolic changes were seen within two-weeks in a dietary intervention with 10 participants[66]. Based on these different approaches, we conclude that a sample size of 16 at the end of the study is sufficient. Assuming a 33% dropout rate we estimate that it will be necessary to enrol n=24 NAFLD participants to achieve n=16 completing the two diet-interventions. This dropout rate has been reported in other dietary interventional studies[67].

Statistical Analysis Plan

For peer review only - http://bmjopen.bmj.com/site/about/guidelines.xhtml

Baseline characteristics of participants, outcome measures at baseline (liver fat content, blood biomarkers and gut microbial composition) at each period time-point will be summarized by diet group using descriptive statistics. The differences between the two interventions (LGI and HGI diet) will be tested by matched-pair Student's t-test. Two-way repeated measures ANOVA test will be used to evaluate the effects of diets and time sequences on liver fat content, as well as on gut microbial composition and blood biomarkers and/or interactions with liver fat content, gut microbial composition and blood biomarkers. Data will be presented as mean± standard deviation and the significance of difference will be set at $p < 0.05$. Any changes in the planned statistical methods will be documented in the trial report. Data input, cleaning and analysis will be conducted using Stata software package. All analyses will be conducted on University of Nottingham computers/laptops that are regularly backed up to University of Nottingham servers.

Microbiome analyses: OTUs with a relative abundance of <0.1% in every sample will be removed, and relative OTU abundances will be inverse normal transformed before further analysis. Associations between response to LGI diet and OTU abundance at genus level will be predicted using a general linear model adjusted for age, gender, and BMI. OTUs will be assumed to be significantly associated with response to the dietary intervention with a value <0.05 after adjusting for false discovery rate (FDR).

DATA MANAGEMENT

Trial data will be managed by study coordinator (AA) under the supervision of chief investigator (GA) and study statistician (LB). The study coordinator shall carry out monitoring of trial data as

For peer review only - http://bmjopen.bmj.com/site/about/guidelines.xhtml

an ongoing activity and perform annual site system audit. Trial data and evidence of monitoring and systems audits will be made available for inspection by REC as required. The Sponsor has the right to take advice from the Trial Steering Committee and Data Monitoring Committee as appropriate for study discontinuation. All trial staff and investigators will endeavour to protect the rights of the trial's participants to privacy and informed consent, and will adhere to the Data Protection Act, 2018. Access to the data will be limited to the trial staff and investigators and relevant regulatory authorities and will be restricted by user identifiers and passwords (using a one-way encryption method). Computer held data including the trial database will be held securely and password protected. All data will be stored on a secure dedicated web server. Electronic data will be backed up every 24 hours to both local and remote media in encrypted format.

In compliance with the ICH/GCP guidelines, regulations and in accordance with the University of Nottingham Research Code of Conduct and Research Ethics, the Chief or local Principal Investigator will maintain all records and documents regarding the conduct of the study. These will be retained for at least 7 years or for longer if required. The Trial Master File and trial documents held by the Chief Investigator on behalf of the Sponsor shall be finally archived at secure archive facilities at the University of Nottingham. This archive shall include all trial databases and associated meta-data encryption codes.

Ethics and dissemination

Ethical approval was first obtained from the University of Nottingham, Medical School Research Ethics Committee. Then additional ethical approval was obtained from East Midlands Nottingham 2 Research Ethics Committee (The Old Chapel, Royal Standard Place, Nottingham). The trial is

For peer review only

registered within ClinicalTrials.gov (NCT04415632, protocol v1.1, 28/10/2019). Within a five-year timeframe, the final anonymised trial data will be uploaded to the https://rdmc.nottingham.ac.uk public repository after consistency and quality have been verified by the project team and publication of the results. Data from this trial are intended to be presented at local and international conferences including those attended by clinicians and dietitians and will also be used as part of a Philosophy Doctorate thesis. Publications will be in peer reviewed journals. No participant will be identified in any of these publications.

DISCUSSION AND RESEARCH IMPLICATIONS:

Building on previous findings from Morgan *et al*[41] and Bawden *et al*,[21], this study will investigate the effects of LGI versus HGI diets in participants diagnosed with NAFLD, over a two-week period. Whilst previous studies showed a significant impact of LGI on liver fat reduction and other metabolic biomarkers, questions remain on the effect in individuals with NAFLD and the longer-term impact. Due to the carefully controlled nature of the intervention, the sample size can be reduced, and the research used to probe more specifically into the direct effects of varying the type of carbohydrate intake. Many studies have shown that energy reduction has a profound effect on liver fat and metabolic health[68], but these diets have been found to be unsustainable in a community setting[10]. Proof of the direct impact of varying a specific characteristic of one macronutrient without reducing the overall intake of the macronutrient consumed, or the overall energy consumed would provide a strong basis for further investigations on the sustainability of such diets.

For peer review only

This study takes advantage of advanced MR protocols which provide a powerful method for non-invasively assessing the effects of lifestyle interventions on metabolic health alongside other physiological measures. Biopsy measurements are uncomfortable for the patient carrying a number of significant risks and would be ethically inappropriate in otherwise healthy individuals. In addition, biopsy only provides a small sample from one region of the liver. MRI and MRS have been well validated[52 69] and allows for repeated tissue specific measurements of fat fraction with minimal risk to study volunteers, thus enabling robust longitudinal studies on the impact of nutrition on metabolic health and liver disease. Additionally, recent research has highlighted the potential significance of lipid composition in metabolic disorders and novel methodologies for measuring this using MRI and MRS have been developed[53]. These advanced techniques will be used to assess not only lipid accumulation but also the effect on saturated, unsaturated, and polyunsaturated fat stores in the liver. Subcutaneous and visceral fat volumes will also be obtained alongside hepatic fat fraction during the scan session giving a more complete picture of abdominal lipid deposition.

^{31}P MRS measurements of hepatic ATP flux provides a novel and exploratory assessment of the impact of lifestyle interventions on metabolism. Previous studies have shown the correlation of metabolic disorders such as NASH with impaired ATP turnover in the liver[70]. Using advanced in-vivo saturation transfer MR protocols, this study will monitor these effects during an intervention and provide the first in-human study to explore mechanistic effects of diet on liver lipid accumulation. This will give a foundation for future research projects and help to advance the effort against the growing global pandemic of NAFLD and related disorders.

For peer review only

Outcomes from this study will provide evidence on the effects of a LGI diet consumption on measures of NAFLD and will serve as a complementary tool for reducing hepatic fat accumulation in NAFLD patients. It will also be the first to provide nutritional-related information about the effects of LGI diet on gut microbiota composition in this medical condition. Accordingly, trial findings will open the way for academic beneficiaries (including dietitians, hepatologists, diabetologists) to develop a novel strategy for NAFLD monitoring through understanding the mechanism behind the LGI dietary-related effects of specific gut microbiota and hepatic fat accumulation.

In addition, the validation findings for the use of CAP-Fibroscan™ as a new tool for assessing hepatic fat levels in dietary interventions has the potential to enhance diagnostic and research capacity. Furthermore, the validation of Norfolk-EPIC FFQ in Nottingham population will permit its use in larger population-based studies.

If this study successfully reproduces the reduction in hepatic fat levels through LGI diet reported in healthy volunteers[21] in a NAFLD patient cohort, this will be a key step towards developing a new option for dietary intervention. The inclusion of LGI foods as a recommendation for NAFLD patients may have substantial beneficial impacts on society as well. It will first enhance the quality of life, health and well-being of NAFLD patients by reducing the burden of NAFLD progression to advanced liver diseases and more likely to be practical and thriving in the long term as it is the first non-calorie restriction approach. Second, it will contribute towards evidence-based policy making in GI food labelling and therapeutic dietary recommendations and will contribute to increase public awareness of dietary issues. The potential medical costs for a patient with NAFLD per year is between €354 to €1163 in Europe[3]. Thus, preventing NAFLD progression will reduce

For peer review only

the burden on health care systems and clinical services as NAFLD patients have high-risk liver-related morbidities and metabolic comorbidities. Also, it may urge the food industries and services to adopt labelling GI values in nutrition facts labels of food products and to reformulate high GI foods to make them lower. However, this study protocol is subject to certain limitations. First, the 2 week time frame of the intervention, especially in light of isoenergetic diet, may not be long enough to observe significant changes in liver fat and microbiome. Second, findings from small sample size may not be generalizable to all patients with NAFLD so will require replication in larger long-term randomised controlled trial.

AUTHORS CONTRIBUTIONS

Amina Al-Awadi, Guruprasad Aithal, Jane Grove, Moira Taylor, Ana Valdes, Amrita Vijay, and Stephen Bawden conceived the study and designed the protocol. Moira Taylor and Amina Al-Awadi contributed to diet planning. Stephen Bawden and Penny Gowland involved in the discussion of initial study design and subsequent outline and contributed to the planning of MRI/S data collection and analysis. Amina Al-Awadi drafted the manuscript and all authors revised the manuscript critically.

FUNDING This study is funded by University of Nottingham studentship to Amina Al-Awadi and National Institute for Health Research (NIHR) Nottingham Digestive Diseases Biomedical Research Unit and NIHR Nottingham Biomedical Research Centre [BRC-1215-20003] at the Nottingham University Hospitals NHS Trust and University of Nottingham. The study sponsor and funder will have no role in the design, collection,

For peer review only

management, analysis, and interpretation of data; writing of the report; and the decision to submit the report for publication. The study sponsor and funder will have no ultimate authority over any of these activities.

COMPETING INTERESTS None declared

Ethics Approval East Midlands Nottingham 2 Research Ethics Committee (The Old Chapel, Royal Standard Place, Nottingham), REC reference 19/EM/0291.

Acknowledgements:

We thank Lu Ban for her assistance in the calculation of study sample size and acquisition of data. We also thank Andy Wragg and the NIHR Nottingham Biomedical Research Centre (BRC) Digestive Diseases Patient Advisory Group for their support in developing and design of the study. We are also grateful for support from liver and gastrointestinal disorder research team and for advice from Liz Simpson, Prarthana Thiagarajan, and Sally Cordon. The views expressed are those of the authors and not necessarily those of the NHS, the NIHR or the Department of Health.

Legends: Figure 1: Schematic diagram for crossover trial of LGI diet versus HGI diet effects on liver fat content in participants with NAFLD.

REFERENCES

1. Del Chierico F, Gnani D, Vernocchi P, et al. Meta-omic platforms to assist in the understanding of NAFLD gut microbiota alterations: tools and applications. *Int J Mol Sci* 2014;15(1):684-711. doi: 10.3390/ijms15010684
2. Machado MV, Cortez-Pinto H. Diet, Microbiota, Obesity, and NAFLD: A Dangerous Quartet. *Int J Mol Sci* 2016;17(4):481-81. doi: 10.3390/ijms17040481

3. Younossi ZM, Henry L, Bush H, et al. Clinical and Economic Burden of Nonalcoholic Fatty Liver Disease and Nonalcoholic Steatohepatitis. *Clinics in Liver Disease* 2018;22(1):1-10. doi: https://doi.org/10.1016/j.cld.2017.08.001
4. Perumpail BJ, Cholankeril R, Yoo ER, et al. An Overview of Dietary Interventions and Strategies to Optimize the Management of Non-Alcoholic Fatty Liver Disease. *Diseases* 2017;5(4) doi: 10.3390/diseases5040023 [published Online First: 2017/10/27]
5. Zaki M, Yousef W, Kamal S, et al. Association Between Metabolic Abnormalities and Non-Alcoholic Fatty Liver in Obese Premenopausal Women. *Biomedical and Pharmacology Journal* 2018;11(2):1161-66.
6. Lucas C, Lucas G, Lucas N, et al. A systematic review of the present and future of non-alcoholic fatty liver disease. *Clinical and Experimental Hepatology* 2018;4(3):165.
7. Kim D, Touros A, Kim WR. Nonalcoholic fatty liver disease and metabolic syndrome. *Clinics in liver disease* 2018;22(1):133-40.
8. Buzzetti E, Pinzani M, Tsochatzis EA. The multiple-hit pathogenesis of non-alcoholic fatty liver disease (NAFLD). *Metabolism* 2016;65(8):1038-48.
9. Dongiovanni P, Lanti C, Riso P, et al. Nutritional therapy for nonalcoholic fatty liver disease. *The Journal of nutritional biochemistry* 2016;29:1-11.
10. Musso G, Cassader M, Rosina F, et al. Impact of current treatments on liver disease, glucose metabolism and cardiovascular risk in non-alcoholic fatty liver disease (NAFLD): a systematic review and meta-analysis of randomised trials. *Diabetologia* 2012;55(4):885-904.
11. Conlon BA, Beasley JM, Aebersold K, et al. Nutritional management of insulin resistance in nonalcoholic fatty liver disease (NAFLD). *Nutrients* 2013;5(10):4093-114. doi: 10.3390/nu5104093 [published Online First: 2013/10/25]
12. Vos MB. Nutrition, nonalcoholic fatty liver disease and the microbiome: recent progress in the field. *Current opinion in lipidology* 2014;25(1):61.
13. McCarthy EM, Rinella ME. The role of diet and nutrient composition in nonalcoholic fatty liver disease. *Journal of the Academy of Nutrition and Dietetics* 2012;112(3):401-09.
14. Jenkins D, Wolever T, Jenkins A, et al. The glycaemic index of foods tested in diabetic patients: a new basis for carbohydrate exchange favouring the use of legumes. *Diabetologia* 1983;24(4):257-64.
15. Thompson D, Capes S, Feig D, et al. Canadian Diabetes Association 2008 clinical practice guidelines for the prevention and management of diabetes in Canada. *Can J Diabetes* 2008;32:S168-S80.
16. Parker A, Kim Y. The Effect of Low Glycemic Index and Glycemic Load Diets on Hepatic Fat Mass, Insulin Resistance, and Blood Lipid Panels in Individuals with Nonalcoholic Fatty Liver Disease. *Metabolic syndrome and related disorders* 2019;17(8):389-96.
17. Rivera I. Impact of a low fructose, low glycemic index and low glycemic load dietary intervention on liver function, body composition and cardio metabolic risk factors in children and adolescents with nonalcoholic fatty liver disease. 2013
18. Valtuena S, Pellegrini N, Ardigo D, et al. Dietary glycemic index and liver steatosis. *The American journal of clinical nutrition* 2006;84(1):136-42.
19. Finelli C, Tarantino G. Is there any consensus as to what diet or lifestyle approach is the right one for NAFLD patients. *J Gastrointestin Liver Dis* 2012;21(3):293-302.
20. Freidoony L, Kong ID. Practical approaches to the nutritional management of nonalcoholic fatty liver disease. *Integrative Medicine Research* 2014;3(4):192-97.

For peer review only

21. Bawden S, Stephenson M, Falcone Y, et al. Increased liver fat and glycogen stores after consumption of high versus low glycaemic index food: A randomized crossover study. *Diabetes, Obesity and Metabolism* 2017;19(1):70-77.
22. Tilg H, Kaser A. Gut microbiome, obesity, and metabolic dysfunction. *The Journal of clinical investigation* 2011;121(6):2126-32.
23. Albenberg LG, Wu GD. Diet and the intestinal microbiome: associations, functions, and implications for health and disease. *Gastroenterology* 2014;146(6):1564-72.
24. Carmody RN, Gerber GK, Luevano Jr JM, et al. Diet dominates host genotype in shaping the murine gut microbiota. *Cell host & microbe* 2015;17(1):72-84.
25. Deehan EC, Yang C, Perez-Muñoz ME, et al. Precision Microbiome Modulation with Discrete Dietary Fiber Structures Directs Short-Chain Fatty Acid Production. *Cell Host Microbe* 2020;27(3):389-404.e6. doi: 10.1016/j.chom.2020.01.006 [published Online First: 2020/02/01]
26. Valdes AM, Walter J, Segal E, et al. Role of the gut microbiota in nutrition and health. *Bmj* 2018;361:k2179. doi: 10.1136/bmj.k2179 [published Online First: 2018/06/15]
27. Wu GD, Compher C, Chen EZ, et al. Comparative metabolomics in vegans and omnivores reveal constraints on diet-dependent gut microbiota metabolite production. *Gut* 2016;65(1):63-72. doi: 10.1136/gutjnl-2014-308209 [published Online First: 2014/11/29]
28. Voreades N, Kozil A, Weir TL. Diet and the development of the human intestinal microbiome. *Frontiers in microbiology* 2014;5:494.
29. David LA, Maurice CF, Carmody RN, et al. Diet rapidly and reproducibly alters the human gut microbiome. *Nature* 2014;505(7484):559-63.
30. Singh RK, Chang H-W, Yan D, et al. Influence of diet on the gut microbiome and implications for human health. *Journal of translational medicine* 2017;15(1):73.
31. Bäckhed F, Ding H, Wang T, et al. The gut microbiota as an environmental factor that regulates fat storage. *Proceedings of the national academy of sciences* 2004;101(44):15718-23.
32. Cani PD, Delzenne NM. The role of the gut microbiota in energy metabolism and metabolic disease. *Curr Pharm Des* 2009;15(13):1546-58. doi: 10.2174/138161209788168164 [published Online First: 2009/05/16]
33. D'Aversa F, Tortora A, Ianiro G, et al. Gut microbiota and metabolic syndrome. *Intern Emerg Med* 2013;8 Suppl 1:S11-5. doi: 10.1007/s11739-013-0916-z [published Online First: 2013/03/08]
34. Egshatyan L, Kashtanova D, Popenko A, et al. Gut microbiota and diet in patients with different glucose tolerance. *Endocr Connect* 2016;5(1):1-9. doi: 10.1530/EC-15-0094 [published Online First: 2015/11/10]
35. Kau AL, Ahern PP, Griffin NW, et al. Human nutrition, the gut microbiome and the immune system. *Nature* 2011;474(7351):327-36. doi: 10.1038/nature10213 [published Online First: 2011/06/17]
36. Aron-Wisnewsky J, Gaborit B, Dutour A, et al. Gut microbiota and non-alcoholic fatty liver disease: new insights. *Clinical Microbiology and Infection* 2013;19(4):338-48.
37. Machado MV, Cortez-Pinto H. Diet, microbiota, obesity, and NAFLD: a dangerous quartet. *Int J Mol Sci* 2016;17(4):481.
38. Miele L, Marrone G, Lauritano C, et al. Gut-liver axis and microbiota in NAFLD: insight pathophysiology for novel therapeutic target. *Current Pharmaceutical Design* 2013;19(29):5314-24.

For peer review only

39. Koh A, De Vadder F, Kovatcheva-Datchary P, et al. From Dietary Fiber to Host Physiology: Short-Chain Fatty Acids as Key Bacterial Metabolites. *Cell* 2016;165(6):1332-45. doi: 10.1016/j.cell.2016.05.041 [published Online First: 2016/06/04]
40. Wesolowski SR, Kasmi KCE, Jonscher KR, et al. Developmental origins of NAFLD: a womb with a clue. *Nature Reviews Gastroenterology & Hepatology* 2017;14(2):81-96. doi: 10.1038/nrgastro.2016.160
41. Morgan LM, Shi J-W, Hampton SM, et al. Effect of meal timing and glycaemic index on glucose control and insulin secretion in healthy volunteers. *British journal of nutrition* 2012;108(7):1286-91.
42. Caussy C, Alquiraish MH, Nguyen P, et al. Optimal threshold of controlled attenuation parameter with MRI-PDFF as the gold standard for the detection of hepatic steatosis. *Hepatology* 2018;67(4):1348-59.
43. McKeown NM, Day NE, Welch AA, et al. Use of biological markers to validate self-reported dietary intake in a random sample of the European Prospective Investigation into Cancer United Kingdom Norfolk cohort. *Am J Clin Nutr* 2001;74(2):188-96. doi: 10.1093/ajcn/74.2.188 [published Online First: 2001/07/27]
44. International Physical Activity Questionnaire. 2002, August [Available from: https://sites.google.com/site/theipaq/.
45. Henry C. Basal metabolic rate studies in humans: measurement and development of new equations. *Public health nutrition* 2005;8(7a):1133-52.
46. Dietary reference values for food energy and nutrients for the United Kingdom. Report of the Panel on Dietary Reference Values of the Committee on Medical Aspects of Food Policy. *Rep Health Soc Subj (Lond)* 1991;41:1-210. [published Online First: 1991/01/01]
47. Henry CJK, Lightowler HJ, Strik CM, et al. Glycaemic index and glycaemic load values of commercially available products in the UK. *British journal of nutrition* 2005;94(6):922-30.
48. Mano F, Ikeda K, Joo E, et al. The effect of white rice and white bread as staple foods on gut microbiota and host metabolism. *nutrients* 2018;10(9):1323.
49. Farup PG, Jacobsen M, Ligaarden SC, et al. Probiotics, symptoms, and gut microbiota: what are the relations? A randomized controlled trial in subjects with irritable bowel syndrome. *Gastroenterol Res Pract* 2012;2012:214102. doi: 10.1155/2012/214102 [published Online First: 2012/08/18]
50. Stephenson M, Leverton E, Khoo E, et al. Variability in fasting lipid and glycogen contents in hepatic and skeletal muscle tissue in subjects with and without type 2 diabetes: a 1H and 13C MRS study. *NMR in Biomedicine* 2013;26(11):1518-26.
51. Hamilton G, Middleton MS, Bydder M, et al. Effect of PRESS and STEAM sequences on magnetic resonance spectroscopic liver fat quantification. *Journal of Magnetic Resonance Imaging: An Official Journal of the International Society for Magnetic Resonance in Medicine* 2009;30(1):145-52.
52. Szczepaniak LS, Babcock EE, Schick F, et al. Measurement of intracellular triglyceride stores by H spectroscopy: validation in vivo. *American Journal of Physiology-Endocrinology And Metabolism* 1999;276(5):E977-E89.
53. Johnson NA, Walton DW, Sachinwalla T, et al. Noninvasive assessment of hepatic lipid composition: advancing understanding and management of fatty liver disorders. *Hepatology* 2008;47(5):1513-23.

For peer review only

54. Bydder M, Yokoo T, Hamilton G, et al. Relaxation effects in the quantification of fat using gradient echo imaging. *Magnetic resonance imaging* 2008;26(3):347-59.
55. Karlas T, Petroff D, Garnov N, et al. Non-invasive assessment of hepatic steatosis in patients with NAFLD using controlled attenuation parameter and 1 H-MR spectroscopy. *PLoS One* 2014;9(3):e91987.
56. Eddowes PJ, Sasso M, Allison M, et al. Accuracy of FibroScan controlled attenuation parameter and liver stiffness measurement in assessing steatosis and fibrosis in patients with nonalcoholic fatty liver disease. *Gastroenterology* 2019;156(6):1717-30.
57. Imajo K, Honda Y, Kessoku T, et al. Magnetic Resonance Imaging More Accurately Classifies Steatosis and Liver Fibrosis in Non-Alcoholic Fatty Liver Disease than Transient Elastography. *Journal of Hepatology* 2016;64(2):S175-S76.
58. Chen C, Stephenson MC, Peters A, et al. 31P magnetization transfer magnetic resonance spectroscopy: Assessing the activation induced change in cerebral ATP metabolic rates at 3 T. *Magnetic Resonance in Medicine* 2018;79(1):22-30. doi: 10.1002/mrm.26663
59. Valkovič L, Chmelík M, Krššák M. In-vivo(31)P-MRS of skeletal muscle and liver: A way for non-invasive assessment of their metabolism. *Anal Biochem* 2017;529:193-215. doi: 10.1016/j.ab.2017.01.018 [published Online First: 2017/01/26]
60. Keller J, Kahlhöfer J, Peter A, et al. Effects of Low versus High Glycemic Index Sugar-Sweetened Beverages on Postprandial Vasodilatation and Inactivity-Induced Impairment of Glucose Metabolism in Healthy Men. *Nutrients* 2016;8(12):802. doi: 10.3390/nu8120802
61. Müller M, Hernández MAG, Goossens GH, et al. Circulating but not faecal short-chain fatty acids are related to insulin sensitivity, lipolysis and GLP-1 concentrations in humans. *Scientific Reports* 2019;9(1):12515. doi: 10.1038/s41598-019-48775-0
62. Liebisch G, Ecker J, Roth S, et al. Quantification of Fecal Short Chain Fatty Acids by Liquid Chromatography Tandem Mass Spectrometry-Investigation of Pre-Analytic Stability. *Biomolecules* 2019;9(4) doi: 10.3390/biom9040121 [published Online First: 2019/03/31]
63. Astbury S, Atallah E, Vijay A, et al. Lower gut microbiome diversity and higher abundance of proinflammatory genus Collinsella are associated with biopsy-proven nonalcoholic steatohepatitis. *Gut Microbes* 2020;11(3):569-80. doi: 10.1080/19490976.2019.1681861 [published Online First: 2019/11/08]
64. Fernandes G, Tap J, Bruls T, et al. Enterotypes of the human gut microbiome. *Nature* 2011;473(7346):174180.
65. D'Argenio V, Salvatore F. The role of the gut microbiome in the healthy adult status. *Clinica chimica acta* 2015;451:97-102.
66. Farshchi HR, Taylor MA, Macdonald IA. Beneficial metabolic effects of regular meal frequency on dietary thermogenesis, insulin sensitivity, and fasting lipid profiles in healthy obese women. *The American journal of clinical nutrition* 2005;81(1):16-24.
67. Samaha FF, Iqbal N, Seshadri P, et al. A low-carbohydrate as compared with a low-fat diet in severe obesity. *New England Journal of Medicine* 2003;348(21):2074-81.
68. Taylor R. Type 2 diabetes: etiology and reversibility. *Diabetes care* 2013;36(4):1047-55.
69. Reeder SB, Sirlin CB. Quantification of liver fat with magnetic resonance imaging. *Magnetic Resonance Imaging Clinics* 2010;18(3):337-57.
70. Traussnigg S, Kienbacher C, Gajdošík M, et al. Ultra-high-field magnetic resonance spectroscopy in non-alcoholic fatty liver disease: Novel mechanistic and diagnostic

For peer review only

insights of energy metabolism in non-alcoholic steatohepatitis and advanced fibrosis. *Liver Int* 2017;37(10):1544-53. doi: 10.1111/liv.13451 [published Online First: 2017/05/20]

For peer review only

Title: Effects of an iso-energetic low glycaemic index (GI) diet on liver fat accumulation and gut microbiota composition in patients with non- alcoholic fatty liver disease (NAFLD): An efficacy mechanism evaluation

Figure 1: Schematic diagram for crossover trial of LGI diet versus HGI diet effects onliver fat content in participants with NAFLD.

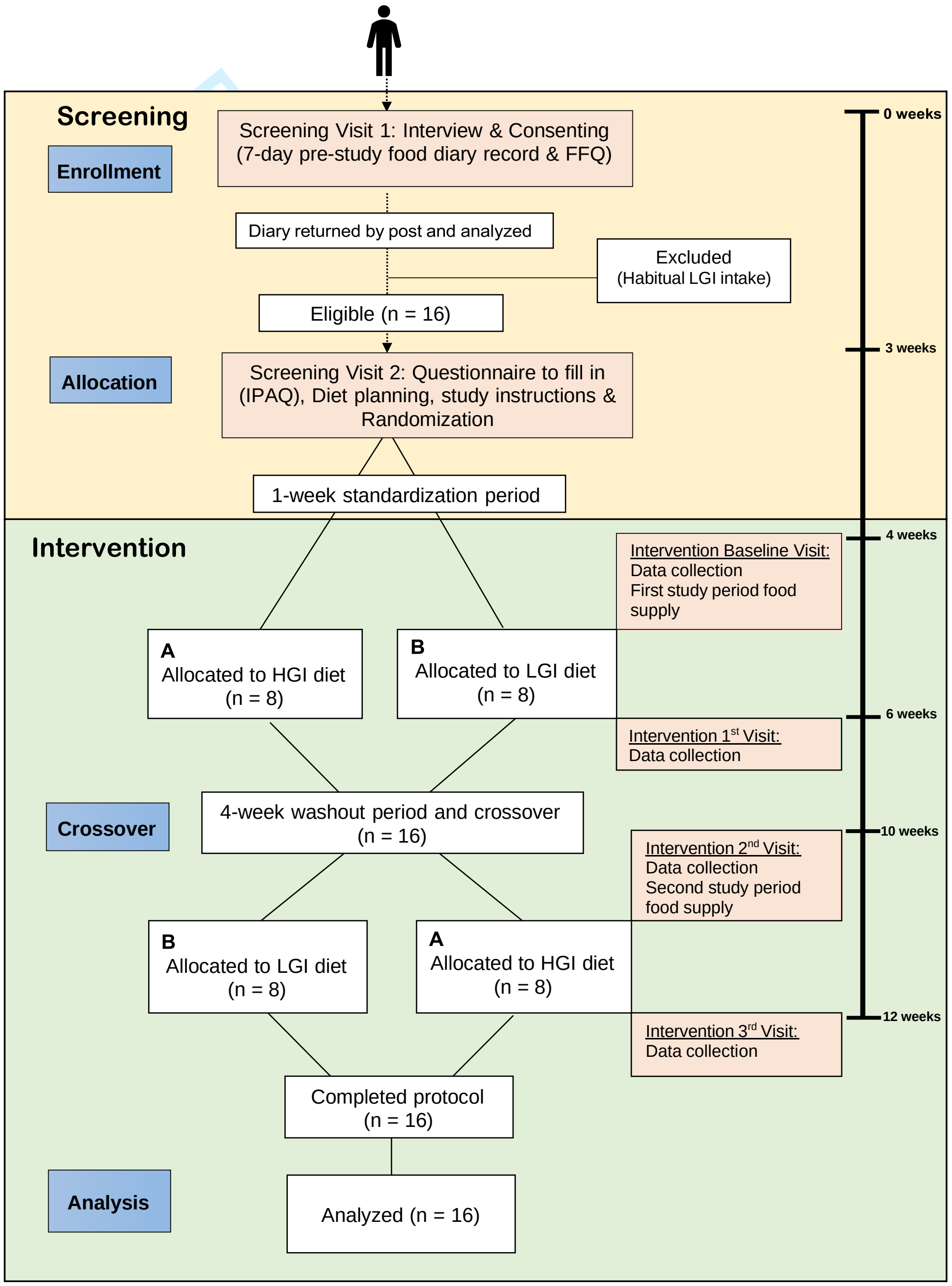

SPIRIT

STANDARD PROTOCOL ITEMS: RECOMMENDATIONS FOR INTERVENTIONAL TRIALS

For peer review only

SPIRIT 2013 Checklist: Recommended items to address in a clinical trial protocol and related documents*

Section/item	Item No	Description	Addressed on page number
**Administrative information**			
Title	1	Descriptive title identifying the study design, population, interventions, and, if applicable, trial acronym	___ 1__________
Trial registration	2a	Trial identifier and registry name. If not yet registered, name of intended registry	____3_________ _
	2b	All items from the World Health Organization Trial Registration Data Set	____9,10_______
Protocol version	3	Date and version identifier	__3 &23 ________
Funding	4	Sources and types of financial, material, and other support	__27_________
Roles and responsibilities	5a	Names, affiliations, and roles of protocol contributors	__1 & 27______
	5b	Name and contact information for the trial sponsor	__27________
	5c	Role of study sponsor and funders, if any, in study design; collection, management, analysis, and interpretation of data; writing of the report; and the decision to submit the report for publication, including whether they will have ultimate authority over any of these activities	__27_________

	5d	Composition, roles, and responsibilities of the coordinating centre, steering committee, endpoint adjudication committee, data management team, and other individuals or groups overseeing the trial, if applicable (see Item 21a for data monitoring committee)	___22,23 ________
**Introduction**			
Background and rationale	6a	Description of research question and justification for undertaking the trial, including summary of relevant studies (published and unpublished) examining benefits and harms for each intervention	_5,6,7________
	6b	Explanation for choice of comparators	_5,6,7_________
Objectives	7	Specific objectives or hypotheses	__8___________
Trial design	8	Description of trial design including type of trial (eg, parallel group, crossover, factorial, single group), allocation ratio, and framework (eg, superiority, equivalence, noninferiority, exploratory)	__9,10_________
**Methods: Participants, interventions, and outcomes**			
Study setting	9	Description of study settings (eg, community clinic, academic hospital) and list of countries where data will be collected. Reference to where list of study sites can be obtained	___9,10________ _
Eligibility criteria	10	Inclusion and exclusion criteria for participants. If applicable, eligibility criteria for study centres and individuals who will perform the interventions (eg, surgeons, psychotherapists)	___10_________
Interventions	11a	Interventions for each group with sufficient detail to allow replication, including how and when they will be administered	_11,12,13,14,15 __
	11b	Criteria for discontinuing or modifying allocated interventions for a given trial participant (eg, drug dose change in response to harms, participant request, or improving/worsening disease)	_____10________
	11c	Strategies to improve adherence to intervention protocols, and any procedures for monitoring adherence (eg, drug tablet return, laboratory tests)	_____15________

For peer review only - http://bmjopen.bmj.com/site/about/guidelines.xhtml

	11d	Relevant concomitant care and interventions that are permitted or prohibited during the trial	____11,15______
Outcomes	12	Primary, secondary, and other outcomes, including the specific measurement variable (eg, systolic blood pressure), analysis metric (eg, change from baseline, final value, time to event), method of aggregation (eg, median, proportion), and time point for each outcome. Explanation of the clinical relevance of chosen efficacy and harm outcomes is strongly recommended	___16________
Participant timeline	13	Time schedule of enrolment, interventions (including any run-ins and washouts), assessments, and visits for participants. A schematic diagram is highly recommended (see Figure)	__12,13, Figure 1
Sample size	14	Estimated number of participants needed to achieve study objectives and how it was determined, including clinical and statistical assumptions supporting any sample size calculations	___20,21_____
Recruitment	15	Strategies for achieving adequate participant enrolment to reach target sample size	_9,10,20,21_____

Methods: Assignment of interventions (for controlled trials)

Allocation:

Sequence generation	16a	Method of generating the allocation sequence (eg, computer-generated random numbers), and list of any factors for stratification. To reduce predictability of a random sequence, details of any planned restriction (eg, blocking) should be provided in a separate document that is unavailable to those who enrol participants or assign interventions	___12________
Allocation concealment mechanism	16b	Mechanism of implementing the allocation sequence (eg, central telephone; sequentially numbered, opaque, sealed envelopes), describing any steps to conceal the sequence until interventions are assigned	_____12_______
Implementation	16c	Who will generate the allocation sequence, who will enrol participants, and who will assign participants to interventions	___12________
Blinding (masking)	17a	Who will be blinded after assignment to interventions (eg, trial participants, care providers, outcome assessors, data analysts), and how	___12________

	17b	If blinded, circumstances under which unblinding is permissible, and procedure for revealing a participant's allocated intervention during the trial	____12________

Methods: Data collection, management, and analysis

Data collection methods	18a	Plans for assessment and collection of outcome, baseline, and other trial data, including any related processes to promote data quality (eg, duplicate measurements, training of assessors) and a description of study instruments (eg, questionnaires, laboratory tests) along with their reliability and validity, if known. Reference to where data collection forms can be found, if not in the protocol	____16_______
	18b	Plans to promote participant retention and complete follow-up, including list of any outcome data to be collected for participants who discontinue or deviate from intervention protocols	____10_______
Data management	19	Plans for data entry, coding, security, and storage, including any related processes to promote data quality (eg, double data entry; range checks for data values). Reference to where details of data management procedures can be found, if not in the protocol	____13_______
Statistical methods	20a	Statistical methods for analysing primary and secondary outcomes. Reference to where other details of the statistical analysis plan can be found, if not in the protocol	_____21,22____
	20b	Methods for any additional analyses (eg, subgroup and adjusted analyses)	____21,22_____
	20c	Definition of analysis population relating to protocol non-adherence (eg, as randomised analysis), and any statistical methods to handle missing data (eg, multiple imputation)	____21,22____

Methods: Monitoring

Data monitoring	21a	Composition of data monitoring committee (DMC); summary of its role and reporting structure; statement of whether it is independent from the sponsor and competing interests; and reference to where further details about its charter can be found, if not in the protocol. Alternatively, an explanation of why a DMC is not needed	_____23______
	21b	Description of any interim analyses and stopping guidelines, including who will have access to these interim results and make the final decision to terminate the trial	_____N/A______

Harms	22	Plans for collecting, assessing, reporting, and managing solicited and spontaneously reported adverse events and other unintended effects of trial interventions or trial conduct	______23______
Auditing	23	Frequency and procedures for auditing trial conduct, if any, and whether the process will be independent from investigators and the sponsor	_____23,25______
**Ethics and dissemination**			
Research ethics approval	24	Plans for seeking research ethics committee/institutional review board (REC/IRB) approval	_____25______
Protocol amendments	25	Plans for communicating important protocol modifications (eg, changes to eligibility criteria, outcomes, analyses) to relevant parties (eg, investigators, REC/IRBs, trial participants, trial registries, journals, regulators)	_____25_______
Consent or assent	26a	Who will obtain informed consent or assent from potential trial participants or authorised surrogates, and how (see Item 32)	_____10_______
	26b	Additional consent provisions for collection and use of participant data and biological specimens in ancillary studies, if applicable	_____N/A_______
Confidentiality	27	How personal information about potential and enrolled participants will be collected, shared, and maintained in order to protect confidentiality before, during, and after the trial	_____25_______
Declaration of interests	28	Financial and other competing interests for principal investigators for the overall trial and each study site	_____26_______
Access to data	29	Statement of who will have access to the final trial dataset, and disclosure of contractual agreements that limit such access for investigators	_____25_______
Ancillary and post-trial care	30	Provisions, if any, for ancillary and post-trial care, and for compensation to those who suffer harm from trial participation	_____N/A_______
Dissemination policy	31a	Plans for investigators and sponsor to communicate trial results to participants, healthcare professionals, the public, and other relevant groups (eg, via publication, reporting in results databases, or other data sharing arrangements), including any publication restrictions	______25_______
	31b	Authorship eligibility guidelines and any intended use of professional writers	_____25_______

For peer review only

	31c	Plans, if any, for granting public access to the full protocol, participant-level dataset, and statistical code	____25_______
**Appendices**			
Informed consent materials	32	Model consent form and other related documentation given to participants and authorised surrogates	____S1,S2____ __
Biological specimens	33	Plans for collection, laboratory evaluation, and storage of biological specimens for genetic or molecular analysis in the current trial and for future use in ancillary studies, if applicable	____S1______

*It is strongly recommended that this checklist be read in conjunction with the SPIRIT 2013 Explanation & Elaboration for important clarification on the items. Amendments to the protocol should be tracked and dated. The SPIRIT checklist is copyrighted by the SPIRIT Group under the Creative Commons "Attribution-NonCommercial-NoDerivs 3.0 Unported" license.

For peer review only
